# A Deeper Insight into the Tick Salivary Protein Families under the Light of Alphafold2 and Dali: Introducing the TickSialoFam 2.0 Database

**DOI:** 10.3390/ijms232415613

**Published:** 2022-12-09

**Authors:** Ben J. Mans, John F. Andersen, José M. C. Ribeiro

**Affiliations:** 1 Epidemiology, Parasites and Vectors, Agricultural Research Council—Onderstepoort Veterinary Research, Onderstepoort, Pretoria 0110, South Africa; 2 Department of Life and Consumer Sciences, University of South Africa, Pretoria 0002, South Africa; 3 Department of Veterinary and Tropical Diseases, University of Pretoria, Pretoria 0002, South Africa; 4 Laboratory of Malaria and Vector Research, National Institute of Allergy and Infectious Diseases, Rockville, MD 20852, USA

**Keywords:** medical entomology, tick, salivary glands, structure, classification

## Abstract

Hard ticks feed for several days or weeks on their hosts and their saliva contains thousands of polypeptides belonging to dozens of families, as identified by salivary transcriptomes. Comparison of the coding sequences to protein databases helps to identify putative secreted proteins and their potential functions, directing and focusing future studies, usually done with recombinant proteins that are tested in different bioassays. However, many families of putative secreted peptides have a unique character, not providing significant matches to known sequences. The availability of the Alphafold2 program, which provides in silico predictions of the 3D polypeptide structure, coupled with the Dali program which uses the atomic coordinates of a structural model to search the Protein Data Bank (PDB) allows another layer of investigation to annotate and ascribe a functional role to proteins having so far being characterized as “unique”. In this study, we analyzed the classification of tick salivary proteins under the light of the Alphafold2/Dali programs, detecting novel protein families and gaining new insights relating the structure and function of tick salivary proteins.

## 1. Introduction

The saliva of bloodsucking animals contains a vast array of compounds that inhibit their hosts’ hemostasis (which consists of platelet or thrombocyte aggregation, blood clotting and vasoconstriction) and can also have immunomodulatory properties and counteract their hosts’ tissue repair mechanisms [1]. Hard ticks feed for several days or weeks on their hosts and their saliva contains thousands of polypeptides belonging to dozens of families, as identified by salivary transcriptomes [2]. Bioinformatic methods help to catalog these families, after identification of signal peptides indicative of secretion [3], identification of transmembrane domains [4] help to exclude transcripts that might code for proteins with a signal peptide domain, but the product may be a membrane protein having extracellular domains. Comparison of the coding sequences to protein databases through the blastp tool, or to motif databases using the rpsblast tool against the Conserved Domains Database (CDD), Pfam, Smart and Kog databases helps to identify putative secreted proteins and their potential functions, directing and focusing future studies, usually done with recombinant proteins that are tested in different bioassays. However, many families of putative secreted peptides have a unique character, not providing significant matches to known sequences. Recently, a database named TickSialoFam (TSFam) classified tick salivary proteins based on psiblast-generated position-specific scoring matrices (PSSM) [5], listing 136 groups of tick salivary proteins, from which only 24 had at least one member biochemically characterized. Of these 136 groups, 63 are tick specific, or “unique” as indicated by their primary structure having no significant matches to known proteins.

The availability in 2021 of the Alphafold2 program [6], which provides in silico predictions of the 3D polypeptide structure, coupled with the Dali program [7], which uses the atomic coordinates of a structural model to search the Protein Data Bank (PDB) [8] allowing another layer of investigation to annotate and ascribe a functional role to proteins having so far being characterized as “unique”. In this study, we analyzed the TSFam database classification under the light of the Alphafold2/Dali programs, having made changes in the names and putative function of 15 protein families, and updating the TSFam database to a 2.0 version.

## 2. Results

The Alphafold2/Dali analysis of the tick salivary proteins allowed for changes in our previous classification of these proteins as proposed in the TickSialoFam database [5]. New protein groups were created, such as metalloproteoid, while some protein groups of unknown function or structure were incorporated into previously known groups, and the predicted structures of formerly known groups are here described, together with evolutionary considerations.

### 2.1. The Metalloproteoid Group

#### 2.1.1. The 28 kDa Protein Family—Changed to New Group Metalloproteoid, Metastriate

**Figure 1:**
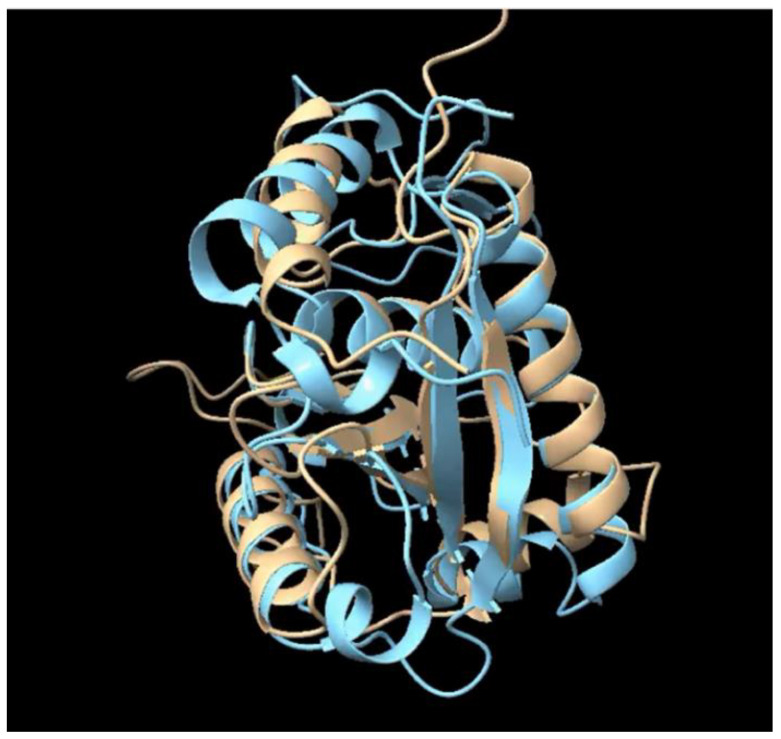
Metalloproteoid JAA55829.1 predicted Alphafold structure (Brown color) superimposed to the metalloprotease leucurolysin-a (PDB 4q1l-A) (Blue color).

The AlphaFold2-Dali (AF-Dali) pipeline identified 58 sequences of the TSFam protein family named “28 kDa”, which appears exclusively in the Metastriata, as producing significant matches (Z values ranging from 7.4 to 14.8) to zinc metalloproteases (spreadsheet S1). Alignment of 28 kDa sequences with their Dali matching metalloproteases, mostly from snake venom, indicate only 2.5% identity and 16.7% similarity (Figure S1). Notice that the zinc-binding histidine motif HEXXHXXGXXH (shown by a rectangle on Figure S1) required for enzymatic activity by the metalloproteases [9] is absent in the tick proteins. Even so, the predicted tertiary structure of the tick proteins matches well with all the beta sheets and most of the alpha helices of the metalloproteases (Figure 1 and movie on Figure S2). We thus rename the 28 kDa family as the Metalloproteoid family, subfamily 28 kDa. The substrate specificity of snake metalloproteases covers a wide range, including coagulation factors, platelet membrane receptors or von Willebrand factor [10]. It is possible that tick metalloproteoids function by binding to specific host proteins without hydrolyzing them, but possibly inhibiting their function, thus acting as kratagonists.

#### 2.1.2. Amb-25-357 Changed to Metalloproteoid, Amblyomma Koch, 1844 (Acari: Ixodidae)

The Alphafold2 predictions of the “unknown” protein family restricted to the *Amblyomma* named Amb-25-357 were matched, with Z values above 14, to metalloproteases (Spreadsheet S1 and Figure S3). Similar to the 28 kDa family above, the alignment of members of this family with metalloproteases shows the absence of the histidine rich domain indicative of zinc ion binding. This family is thus reclassified to the Metalloproteoid group, subgroup *Amblyomma*.

#### 2.1.3. Lipocalin P32 Antigen and Lipocalin, 26 kDa_a—Changed to Metalloproteoid, Subfamilies P32 and 26 kDa_a

The TSFam 1.0 group Lipocalins, sub-groups P32 antigen and 26 kDa_a revealed by the AF-Dali pipeline to be members of the Metalloproteoid group as their AF structure predictions matches metalloproteases with Z values ranging from 10 to 15 for the proteins having MW larger than 17 kDa. All sequences from these groups originated from the *Ixodes* Latreille, 1795 genus.

### 2.2. Reevaluation of the Metalloprotease Family

Having discovered the Metalloproteoid family (see above), we decided to reanalyze the Metalloprotease family identified by the TSFam1.0 database, in particular to certify whether each sequence had the Zn binding domain. We thus searched the motif H-E-x(2)-H-x(2)-G-x(2)-H using the tool ps_scan.pl [11] and discovered that the TSFam1.0 PSMM models 30-49, 30-561, 30-93, 35-121, 35-167, 35-414, 35-415, 35-768, 35-77 and 50-341 were identifying Metalloproteoid sequences. Figure S4 displays three tick protein sequences previously classified as metalloproteases aligned to three snake metalloproteases. Notice the lack of the Zn binding domain on the tick proteins, now classified as members of the Metalloproteoid family.

### 2.3. New Lipocalin Acquisitions

#### Hyp 94 Protein Family—Changed to Lipocalin, Hyp94

The Hyp 94 protein family, which appears exclusive to the genus *Amblyomma*, showed Dali similarities of AF-predicted structures to tick lipocalin proteins named Jacalin and female-specific histamine binding protein [12,13] with Z values ranging from 12 to 15. Thus, it is being reclassified within the Lipocalin group, subgroup Hyp 94. A 3D structure comparison is shown in Figure S5.

### 2.4. New Niemann-Pick Acquisitions

#### 2.4.1. Group 17.7 kDa—Changed to Niemann-Pick, Argasidae

This family of proteins, apparently exclusive to soft ticks, presents AF-Dali similarities with Z values above 12 to Niemann-Pick proteins [14] having a ML domain [15] and accordingly its group is added to the previously existing group “Niemann Pick” forming a new subgroup “argasidae”. These proteins recognize lipids, including pathogen motifs. If secreted in tick saliva they may act as kratagonists. It is interesting that the primary sequence of these proteins does not provide for matches to the pfam02221, E1_DerP2_DerF2, ML domain when searched with the rpsblast tool. However, there is a striking match of the 3D structure between the 17.7 kDa family members and the crystal structure of canonical ML domain containing proteins (movie on Figure S6).

#### 2.4.2. Lipocalin Metastriate—Changed to Niemann-Pick, Metastriate

Similar to the 17.7 kDa group, the Lipocalin Metastriate subgroup was transferred to the Niemann-Pick group.

#### 2.4.3. Cytotoxin Sequences Changed to Niemann-Pick, Der f 7 Antigen and Sulfotransferase Groups

The TSFam1.0 database identified 137 sequences as belonging to the Cytotoxin group. Following AF-DALI results, 94 of these sequences matched the Der f 7 antigen and 32 matched bacterial toxin-like proteins. Two proteins matched protein tyrosine sulfotransferases and 9 did not provide significant structural matches. The two sequences matching sulfotransferases matched by blastp sequences annotated as sulfotransferases had CDD matches to sulfotransferases, and accordingly they were wrongly included in the TSFam Cytotoxin group. The description of the 2 families that were newly identified as characterized by the AF-Dali pipeline follow below.

### 2.5. Cytotoxin

The AF-DALI pipeline identified 32 sequences from ticks of the genus *Ixodes* that matched, with Z values ranging from 10 to 16, bacterial proteins with pdb accessions 2d42-A (NON-TOXIC CRYSTAL PROTEIN), 4rhz-A (CRY23AA1), 3zjx-A (EPSILON-TOXIN), 2ztb-B (CRYSTAL PROTEIN),7ml9-A (INSECTICIDAL PROTEIN), 6lh8-A (AEROLYSIN-LIKE PROTEIN) and 1w3a-A (HEMOLYTIC LECTIN LSLA). These proteins are related to bacterial pore-forming proteins. The presence of these proteins in ticks suggest they were acquired by horizontal transfer, as was the case of the DAE antimicrobial proteins. Figure S7A shows the AF predicted structure for *Ixodes ricinus* (Linnaeus, 1758) JAB71021.1. and in 7B shows the comparison of JAB71021.1 with the dimeric bacterial protein PDB:2d42. Similarly, the DAP-36 protein identified as a *Dermacentor andersoni* Stiles, 1908 immunosuppressive protein [16] matched bacterial toxins. Accordingly, its classification changed from Group DAP-36 to Group Cytotoxin, subgroup DAP-36.

### 2.6. Der f 7 Allergen/JHBP

Protein sequences from both prostriate and metastriate ticks were found matching the structure of Der f 7 allergen with Z scores above 10, and also matching, usually with smaller Z scores, structures annotated as Juvenile Hormone Binding Proteins (JHBP) (Figure S8). The former group Mys-30-94 was identified as a member of the Der f 7 allergen, as were the former group OneOfEach, which were reclassified accordingly. The Der f7 antigen is an allergenic protein derived from the mite *Dermatophagoides farina* Hughes, 1961 (Acari: Pyroglyphidae). While the function of Der f 7 allergen is unknown, JHBP are well known carriers of the lipidic juvenile hormone found in arthropods. It is possible this family acts as lipid kratagonists.

### 2.7. Additional Changes

Following AF-DALI analysis, the following TSFam motifs were changed as indicated below:

40-800, Evasin → changed to Kunitz
40-584, Evasin → changed to acid tail
35-53, Kunitz → changed to Lipocalin
Rapp-25-325 → changed to cystatin
25-159 Cytochrome_P450 → changed to Cytochrome_B2

### 2.8. The Search for Salivary Disintegrins

Disintegrin was the name given to soluble snake venom toxins consisting of peptides varying in length from 49-84 amino acids which were able to prevent platelet aggregation by inhibiting the cross-linking of activated platelets by fibrinogen, which binds to the platelet integrin α_IIb_β_3_ (fibrinogen receptor) [17]. These inhibitors contain an amino acid triad (usually KGD or RGD) flanked, a few amino acids up or down the triad, by cysteines that form a bridge and stabilizes the hairpin structure. The environment around the disintegrin loop has an important role in the curvature and flexibility of this loop, determining the integrin specificity: some RGD disintegrins do interact with α_3_β_1_, α_6_β_1_, and α_7_β_1_ thus interfering with laminin-based cell adhesion while the RGD in jarastatin targets α_M_ß_2_ in neutrophils. KTS and RTS disintegrins, on the other hand, are selective inhibitors of the α_1_β_1_ integrin, a receptor for collagen IV. Later it was shown that snake disintegrins were derived from limited proteolysis of proteases of the ADAM (A Disintegrin And Metalloprotease) and ADAMTS (A Disintegrin And Metalloprotease with Thrombospondin motifs), which are widely found in both invertebrates and vertebrates [9,10,18,19,20].

Saliva of ticks contain disintegrins inhibiting platelet aggregation [21], as well inhibitors of neutrophil aggregation, which were identified by their similarity to ADAMTS metalloproteases [22]. Saliva of sand flies also contain a typical disintegrin [23]. Interestingly, saliva of a horse fly contains a protein named Tablysin-15 which is a member of the widespread antigen 5 family [24] that, however, acquired an RGD domain resulting in a high affinity binding for platelet α_IIb_β_3_ and endothelial cell α_V_β_3_, but not for α_5_β_1_ or α_2_β_1_ integrins [25] thus showing that the disintegrin function can be found in non-canonical protein families. To predict a disintegrin function on a protein it is important that the disintegrin triad is shown as a hairpin protruding from the remaining protein backbone. We thus used ps_scan to scan our tick salivary protein database (Spreadsheet S1) against a disintegrin motifs database (Supplementary File S1), resulting in 804 of the 15,797 sequences, or 5%, displaying at least one disintegrin motif. These results are mapped to column AM of Spreadsheet S1. After searching for the hallmark loop on 100 of these 804 candidate sequences, we found 18 that were strong candidates for further testing of disintegrin activity, eight of which are shown in Figure S9, belonging to different tick salivary families. A complete description of all the predicted disintegrin sequences is out of the scope of this work.

### 2.9. Secreted cyP450 Enzymes

Cytochrome P450 enzymes are ubiquitous heme-containing proteins that catalyze oxidative reactions in steroids, fatty acids, prostaglandins, leukotrienes, biogenic amines, pheromones and plant metabolites [26]. In eukaryotes these enzymes are mostly found bound to membranes. However, we found many tick proteins from salivary transcriptomes that present a clear signal peptide indicative of secretion, no transmembrane domains (outside the signal peptide) or GPI anchors. Because most reactions catalyzed by p450 enzymes involves the oxidative transfer of electrons from the porphyrinic iron to the substrate, a mechanism to regenerate the reduced iron is needed, and this task is associated with the p450 reductase or cytochrome b5 enzymes, which rely on cellular reserves of NADPH. This poses a problem for extracellular p450 enzymes, as they would not be able to access their iron reducing partners [2]. However, p450 enzymes can participate in peroxidase reactions. For example, prostaglandin H_2_ is converted to thromboxane and prostacyclin by two P450s [27,28]. These unusual P450 reactions do not need electrons or O_2_ [29].

The alignment of tick salivary secreted proteins of the p450 family with matching sequences from the PDB database produces the phylogenetic tree depicted in Figure S10, which shows several clusters of sequences, including the large cluster I. The ClustalX alignment of cluster I (Supplementary Figure S11A,B) shows the conserved motif FxxGx(H/R)xCxG associated with the prosthetic heme group (marked by the pink amino acids in Figure S11B). The absolutely conserved cysteine is the proximal ligand to the heme iron. This sulfur is the origin of the characteristic name-giving 450 nm Soret absorbance observed [30]. An additional conserved motif, [AG]-G-x-[ED]-T (indicated by green amino acids in Figure S11B), contains the highly conserved threonine preceded by an acidic residue which is positioned in the active site and believed to be involved in catalysis [30].

To the extent that these peptides coding for P450 enzymes are secreted in saliva, they may function to convert platelet derived PGH_2_ to prostacyclin or PGE_2_, which are inhibitors of platelet aggregation and vasodilators, or they can act solely as kratagonists of lipidic agonists of hemostasis. These possibilities remain untested.

### 2.10. The Salp15/Ixostatin Group

Ixostatin was the name given to a group of cysteine-rich protein sequences found in the sialotranscriptome of *Ixodes pacificus* Cooley and Kohls, 1943 [31] displaying remarkable similarities to the cysteine-rich domain of ADAMST-4 (aggrecanase). Due to their similarities to these domains, they could be involved in disruption of platelet aggregation or neutrophil function, cell–matrix interactions, or inhibition of angiogenesis [31]. A few proteins of this group were characterized functionally. Two recombinant proteins from *Ixodes scapularis* Say, 1821 of the Ixostatin group, namely ISL929 (CAX36743.1) and ISL1373 (CAX36742.1), were shown to reduce expression of β_2_ integrins and impair adherence of polymorphonuclear leukocytes [22]. The ixostatin protein from *I. scapularis* named Salp15 (AAK97817.1) was shown to inhibit CD4+ T cell activation. Repression of calcium fluxes triggered by TCR ligation resulted in lower production of interleukin 2. Salp15 also inhibited the development of CD4+ T cell-mediated immune responses in vivo [32].

Psiblast search of the NR database starting with members of this group, for example, CAX36743.1, finds only tick sequences in the initial blastp, but in subsequent iterations it identifies metalloproteases from insects, without converging to a unique group of sequences. Using more limited psiblast parameters (-j 2 and -h 1e-15) when starting the search with typical members of the group, we created 41 models that identified 632 sequences as belonging to the Salp15/Ixostatin group, out of a total of 15,796 sequences. The vast majority are found in the *Ixodes* genus, but a few sequences are also found in metastriate ticks. No member of this group was found in the Argasidae. Alignment and phylogenetic analysis of selected 197 protein sequences of the Salp15/Ixostatin group shows a shallow distribution of branches, most with low bootstrap support (Figure S12). This is indicative of a scenario of fast evolution with possible events of recombination. The genes coding for these proteins have been proposed to be evolving under positive selection pressure [33]. Alignment of 73 sequences most similar to Salp15 (AAK97817.1), all from the *Ixodes* genus (Figure S13), shows seven conserved cysteines within 19% identities in 109 sites of the mature proteins. Alphafold2 predictions for 80 peptide sequences of the Salp15 family containing seven cysteines shows, on the monomeric prediction mode, three disulfide bonds with the structure |C:1 C:3 |C:2 C:5 |C:4 C:6| (Figure S14). The most carboxyterminal cysteine remains in the reduced state. The NMR structure of a monomeric Salp15 has been recently published [34] and compared to the Alphafold2 prediction. The authors concluded that “the global fold of Salp15 likely consists of a disordered N-terminal region and a globular domain with an α-helix, a ß-sheet, and regions with non-regular structure. Only C135, the C-terminal residue, is reduced, according to its 13C chemical shifts”. Perhaps the free carboxyterminal cysteine could be joined with another in a dimer structure. Submission of these sequences to Alphafold2 in dimer and tetramer mode failed to recover multimers. The disordered N terminal region may impair folding predictions.

Alignment of 43 sequences most similar to Ixostatins ISL929 (CAX36743.1) and ISL1373 (CAX36742.1) reveals 8 conserved cysteines (Figure S15). Alphafold2 structure predictions in the monomeric mode indicate that six of the cysteines are involved in disulfide bonds as A:2 A:4 |A:3 A:6 |A:5 A:7 (Figure S16A), with the first and last cysteines remaining in the reduced state. However, dimer predictions (Figure S16B) assign these residues to be linked as A:1|B:8 and A:8|B:1 in 38 of these 43 sequences, suggesting that a dimer conformation is more probable in this subgroup of the Salp15/Ixostatin group.

The most cys rich subgroup of the Salp15/Ixostatin group, named 20-Cys has 20 or 21 cysteines (Alignment on Figure S17). The Alphafold2 predictions (Figure S18 and spreadsheet S1) indicates the 20 cysteines to be connected as |A:1 A:3 |A:2 A:5 |A:4 A:15 |A:6 A:8 |A:7 A:10 |A:9 A:20 |A:11 A:13 |A:12 A:17 |A:14 A:16 |A:18 A:19. Within the sequences containing 21 cysteines, the most carboxy terminal cysteine remains reduced. Alphafold2 predictions in the multimer mode do not predict these unpaired cysteines to be involved in disulfide bridges.

### 2.11. The 10 k Da-WC Group

The 10 kDa-WC family is a large secretory family thus far restricted to the genus *Ixodes* [35]. It is named based on the WC motif found in the C-terminal end of the protein. PSI-BLAST analysis of the non-redundant database retrieves 107 sequences after an exhaustive search, while the current supplemental spreadsheet S1 contains 353 proteins annotated for this family. At sequence level, the family is characterized by 7 conserved cysteines (although this may vary for individual family members) (Figure S19). Not all members possess the WC motif as can be seen for some sequences. The sequences in the alignment show 27–88% sequence similarity (median 40%). However, a stable compact fold is predicted for all sequences composed of N- and C-terminal interacting alpha-helices packed against a central three-stranded anti-parallel beta-sheet (Figure S21). All modelled structures show 3 intact disulfide bonds with a disulfide bond pattern of C1–C6, C2–C4, C3–C5 (Figure S20). Interestingly, the cysteine involved in the WC motif sits at the C-terminal end and does not have a disulfide bond partner. Its solvent exposure suggests that this cysteine is reactive and would either form dimers with itself or other members of this family or other proteins. A homodimer model predicted by AlphaFold2 result in disulfide bond formation between the respective C7 cysteines with stacking of the tryptophans, suggesting that this may indeed be the natural quaternary conformation of family members that possess the WC motif. The dimer is formed by a two- fold rotation of the alpha helices to form a cleft that may be involved in binding host target proteins or even ligands (Figure S21). The possibility also exists that family members may form heterodimers thereby increasing the potential functional repertoire of this family. Structural alignment of the different monomer models indicates RMSD values that range from 0.59–1.97 Å ± 0.34 Å for alignment of the secondary structure elements. The Dali search algorithm found for the best hit a Z-score of 4.3 and RMSD of 2.6 Å over 90% of the sequence alignment. This match is for a domain of unknown function (DUF2470) found in glutamyl-tRNA reductase binding protein and heme utilization gene Z (HugZ), both belonging to the Heme-binding protein family found in bacteria [36,37]. DUF2470 is involved in binding to glutamyl-tRNA reductase and partially shields the heme-binding pocket for the heme-binding proteins. Potential functions for this family in ticks may include anti-microbial activity by targeting heme-degradation by bacterial heme oxygenases [38]. The binding cleft formed by the dimers may also be functional in a range of other binding activities.

### 2.12. The 5.3 kDa Family

The 5.3 kDa family is restricted to hard ticks with extensive expansions in the genus *Ixodes* [38]. An exhaustive PSI-BLAST search of the non-redundant database retrieves 35 sequences that all belong to *I. scapularis* or *I. ricinus*. The current supplemental spreadsheet S1 report 137 sequences. The family is characterized by 6 conserved cysteine residues with some members having an additional pair or single N-terminal cysteine (Figure S22). Sequence similarity for the presented family members ranges from 11–89% (median 33%). AlphaFold2 predicts a triple-stranded anti-parallel beta-sheet with a cystine knot formed by two of the disulfide bonds (Figure S23). Structural alignment of the different monomer models indicates RMSD values that range from 0.41–2.25 Å ± 0.42 Å for alignment of the secondary structure elements. The disulfide bond structure is the same as other cystine knot family members namely, C1–C4, C2–C5, C3–C6 [39], with some members exhibiting an additional disulfide bond (Figure S22). Recently, holocyclotoxin was shown to exhibit the cystine knot fold [40] and previously the relationship between the 5.3 kDa and holocyclotoxin was noted [2,35]. Expanding on this, the assignment of the 5.3 kDa family to the cysteine knot fold and their conserved disulfide bond pattern was also confirmed [41]. Potential functions for the 5.3 kDa family may be anti-microbial [42] or targeting of ion channels [43]. Of interest is the high structural similarity found with a potato carboxypeptidase inhibitor that also present a knottin fold [44] that gives a RMSD of 1.15 Å. There is therefore a possibility that the 5.3 kDa family may also perform this function.

### 2.13. Seven-Disulfide Bond Family (7 DB)

The seven-disulfide bond family is to date unique to soft ticks and was first described in *Argas monolakensis* Schwan, Corwin & Brown, 1992 (Acari: Argasidae), *Ornithodoros coriaceus* Koch 1844 and *Ornithodoros parkeri* Cooley, 1936 [45,46,47]. PSI-BLAST analysis of the non-redundant database retrieves 2000 proteins after 8 iterations that span all Kingdoms of Life except for Arthropods, indicating that the 7 DB members may belong to a large and extensive protein family. It also suggests that this family may have been acquired via horizontal gene transfer, probably from a vertebrate host. The current database contains 7 members.

It was previously suggested that the 7 DB family is composed of two BTSP domains [47]. Alphafold2 modeling indicates, however, that the 7 DB-family shows an integrated fold where the two BTSP-like domains are not independent entities but are formed by the integration of the various beta-sheets to form a globular domain that cannot be differentiated into sub-domains (Figure S24). The structure starts with a two-stranded anti-parallel beta-sheet as the BTSP domain, but while the first strand of the second three-stranded anti-parallel beta-sheet follows from the first beta-sheet, it does not turn back on itself to complete the beta-sheet but moves into a new three-stranded beta-sheet. The second beta strand of this sheet leaves the sheet to form an alpha helix that connects a new two-stranded antiparallel beta-sheet, which moves to a beta-strand that completes the previous three-stranded beta-sheet. The alpha chain then moves back to the “first” domain to form two anti-parallel beta-sheets that stack against the first beta-strand to form the three-stranded anti-parallel beta-sheet of the “first domain”. Pairwise structural comparison of the members shows RMSD values from 0.66 Å −1.7 Å + −0.27 Å. As expected, the disulfide bond structure also presents an inter-mixing of disulfide bonds between the domains giving the pattern: C1–C5, C2–C4, C3–C13, C6–C14, C7–C10, C8–C12, C9–C11 (Figure S25).

Remarkably, the BTSP Barium sulphate adsorption protein1 (BSAP1) can be structurally fitted to the “first BTSP domain” with an RMSD value of 0.91 Å and to the “second domain” with an RMSD value of 1.57 Å (Figure S24). However, fitting to the “second domain” is in reverse order, with the three-stranded beta-sheet from the second domain preceding the two-stranded beta-sheet. This intermixing of domains also results in a disulfide bond pattern that is different from that of the BTSP fold. Conserved disulfide bonds in “domain 1“include C1–C5, C2–C4 and in “domain 2” C7–C10, C8–C12, C9–C11. The last two conserved disulfide bonds are inter-domain and include C3–C13 and C6–C14.

Structural comparison using the DALI server indicates similarity to Evasin-1 (Z-scores: 3.1–4.5 and RMSD 1.5 Å −2.2 Å) and Evasin-4 (Z-scores: 2.5–3.8 and RMSD 3.3 Å −4.6 Å). Evasin-1 and Evasin-4 have been shown to be evolutionary related [48]. Pairwise comparison of Evasin-1 to the 7 DB family indicates a fit of 1.51 Å to the “first domain” and 0.82 Å to the “second domain” (Figure S26). The fit to the first domain is only for some of the beta-sheets while the overall fit to the second domain is better. It is clear that an evolutionary relationship exists between all of these families, related to their beta-sheet structure (Figure S27). The first domain of the 7 DB fold seems to be closer related to the BTSP family and the second domain to the evasins. In the case of the 7 DB family, strand switching between the domains may have resulted in a more overall stable structure. Strand switching is not common in protein structures but has been observed at least once in blood-feeding bugs, where triabin, a lipocalin, have a switched beta-stranded barrel that does not conform to the canonical continuous eight stranded anti-parallel beta barrel characteristic of the lipocalin family A-H, but presents the order ACBDEFGH [49]. To date, no function has been associated with the 7 DB fold.

### 2.14. Basic Tail Secretory Family (BTSP)

The basic tail secretory proteins were first described from *I. pacificus* and *I. scapularis* as a family of small disulfide-rich proteins [31,35]. Since then, BTSP members have been found in all tick salivary gland transcriptomes sequenced to date and are one of the most abundant secretory protein families [47]. Recently, the structure of BSAP1, a member of the BTSP, was determined using NMR and shown to consist of an N-terminal two-stranded and a C-terminal three-stranded anti-parallel beta-sheet arranged end-to-end and stabilized by three conserved disulfide bonds [50,51]. An exhaustive PSI-BLAST analysis using the sequence of BSAP1, retrieved 142 proteins from the non-redundant database. The current supplemental spreadsheet S1 report 484 sequences. The canonical single BTSP domain is characterized by 6 cysteines followed by a lysine-rich tail. The cysteine spacing may be variable and the basic tail may be exchanged for an acidic tail or no tail. The disulfide bond structure is C1–C3, C2–C5, C4–C6 (Figure S28). Alphafold2 prediction of BSAP1 resulted in a structure with a more defined secondary structure that suggests that the NMR structure represents a more labile structure in solution. Even so, the core structure gave an RMSD of 1.47 Å between the NMR and Alphafold2 predicted structure (Figure S29). For comparative purposes we used the Alphafold2 predicted structure. Structural comparison shows that the core domain is conserved as predicted by Alphafold2 with RMSD values ranging from 0.68–1.95 ± 0.24 Å. The C-terminal tail region is unstructured for most proteins although alpha-helices are sometimes predicted. In some cases, proteins have double-BTSP domains. In regard to this, a special case is the related seven disulfide bond family (7 DB) discussed abovee that does not present the same fold. It was suggested that the BTSP family core domain is similar to EGF domains [51]. DALI searches indicate, however, that the closest hits of the core structure resemble the tick evasin-4 fold [52], and an RMSD of 1.01 Å is obtained between BSAP1 and Evasin-4 for the core two-stranded beta-sheet. The general disulfide bond pattern within this core structure is also conserved. An evolutionary relationship, therefore, exists between the BTSP and evasin-4 family. To date the BTSP family has been implicated in fXa inhibition [53], modulation of fibrinolysis [54], and the complement pathway mediated by lectin [51].

### 2.15. Evasins

The evasins are a well-characterized group of proteins that consist of at least two evolutionary independent families named Evasin A and Evasin B with the latest count from the non-redundant database finding 271 Class A evasins and 128 Class B evasins [48]. The Evasin A group contains Evasin-1 and Evasin-4 for which structures have been determined [52,55]. The structure for the Class A evasins presents a discontinuous N-terminal domain comprised of the N-terminus linked by a disulfide bond to two antiparallel beta-sheets (β3-β4) and a C-terminal domain comprised of 2 anti-parallel beta-sheets with 5 anti-parallel beta-strands and an alpha helix in the order β1-β2-β5-α1-β6-β7 (Figures S30 and S31). The core structure consisting of the various beta-sheets and the alpha helix may be absent from some structures. Its disulfide bond pattern is C1–C3, C2–C6, C4–C7, C5–C8 (Figure S30). The Evasin B group contain Evasin-3 for which a structure has been determined [56,57]. It possesses a three disulfide bond knottin fold composed of an anti-parallel beta-sheet with the topology of β1-β3-β2. The disulfide bond pattern is C1–C4, C2–C5, C3–C6 (Figure S32). Fitting of the Alphafold2 modeled structures indicate an RMSD range of 0.8 Å–2.04 Å ± 0.36 Å that shows a largely correctly predicted fold. The evasin A family in general targets CC chemokines, while the evasin B family targets CXC chemokines [48].

### 2.16. Evolutionary Considerations for the BTSP-Evasin A-7 DB Superfamily

The structural similarities of the BTSP, Evasin A and 7 DB families suggest a common ancestral structural fold from which these families evolved that included at least two beta-sheets stabilized by three to four disulfide bonds. Exhaustive PSI-BLAST analysis of the Evasin A and the BTSP families only retrieve family specific members from ticks and their relationships to each other was only detected once Alphafold2 models were searched against the PDB database using the DALI server. This would partly be due to the small size and high sequence divergence observed in these families but could also suggest that these are proteins that originated exclusively within the tick family. If this is the case, then the ability of the 7 DB family to retrieve distant homologs from all kingdoms of life may suggest that this was the ancestral fold for this superfamily. Gene duplication of N- or C-terminal beta-sheet domains would have then resulted in beta-sheets that had to form continuous structures resulting in either the core BTSP or Evasin A fold (Figure S27). It is of interest that the BTSP family shows the best fit to the N-terminal domain, while the Evasin A family shows the best fit to the C-terminal domain, suggesting that the N-terminal domain gave rise to the BTSP family and the C-terminal domain to the Evasin A family (Scenario 1). Alternatively, the Evasin A fold may have originated from the BTSP fold (Scenario 2). The viability of these scenarios would have depended on a horizontal gene transfer even in the ancestral tick lineage with subsequent duplication of the BTSP or Evasin A folds from the 7 DB fold before divergence of the tick families, with subsequent loss of the 7 DB fold in ixodids.

### 2.17. The 13 kDa-Basic Group

The 13 kDa-basic family is a small secretory family found in prostriate and metastriate ticks. An exhaustive PSI-BLAST search of the non-redundant database retrieved 47 sequences, 16 tick proteins with the rest derived from mites and spiders, while the current supplemental spreadsheet S1 contains 15 proteins annotated for this family. This family is highly conserved with sequence similarity ranging from 63–100% (median 73%). The family is characterized by 6 conserved cysteines with a predicted disulfide bond pattern of C1–C6, C2–C3, C4–C5 (Figure S31). Alphafold2 predicts a compact globular structure formed by six alpha-helices, with all structures presenting intact disulfide bonds (Figure S32). Structural alignment indicates RMSD values that range from 0.2–0.78 Å ± 0.14 Å for alignment of the secondary structure elements. The Dali search algorithm found as structural homologs members of the odorant-binding, pheromone-binding or venom 2 allergen families with average Z-scores of 4.0 ± 0.4 and RMSD of 3.5 ± 0.5 Å over 90% of the sequence alignment [58,59]. Odorant-binding proteins have not yet been found in ticks as prominent ligand scavengers involved in tick-host interactions since the lipocalins generally perform this function [2]. The fact that single family members have been found in prostriate, metastriate and other arachnids may point to a role as a odorant or pheromone-binding protein. A major difference between the various odorant-binding families and the tick proteins is that the odorant-binding proteins from *Anopheles gambiae* Giles, 1902 (Diptera: Culicidae) have a disulfide bond pattern of C1–C3, C2–C5, C4–C6, while the venom allergen 2 protein from the fire ant has a disulfide bond pattern of C1–C2, C3–C4, C5–C6. There is therefore a possibility that these families converged on the same general fold, explaining the absence of odorant-binding proteins in ticks [60,61]. Conversely, an ancient relationship between arachnid and insect odorant-binding proteins was postulated [62]. In this regard, the odorant-binding fold of this family has been previously identified in a study that focused on the tick foreleg tarsi transcriptome and was postulated to play a role in chemosensation [63]. The disulfide bond patterns predicted by the Swiss Model and RaptorX servers in this latter study were identical to the patterns found in the current study and also indicated homology to the odorant-binding proteins.

### 2.18. Lipocalin Family

The lipocalin family is one of the largest and most diverse secretory families found in tick salivary glands [64,65]. PSI-BLAST analysis using the histamine-binding protein 2 (O77421) sequence from the hard tick *Rhipicephalus appendiculatus* (Neumann, 1901) [12] against the non-redundant database retrieves 971 proteins that are restricted to ticks. Similarly, moubatin (A46618) from the soft tick *Ornithodoros moubata* (Murray, 1877) [66,67] retrieves ~1081 proteins restricted to ticks. In both cases, PSI-BLAST analysis was run for 15 iterative cycles with no sign of convergence and of those sequences retrieved only 807 (64%) was shared between the two searches. As such, the PSI-BLAST analysis cannot retrieve all members since it cannot be run to exhaustion, indicating that the position-specific scoring matrices derived from alignment do not cover all possible tick lipocalin sequence variations. This indicates that the family is likely too divergent to be represented by any one position-specific scoring matrix. To address this, the current TSFam database contains 125 PSSM models representing various lipocalin clades that should cover the majority of diverse lipocalins in tick sialomes. The current TSFam database contains 2229 members assigned to the lipocalin group, while other in-house databases that also have representatives from transcriptomes present in the small read archives contain more than 11,000 proteins annotated as lipocalins. Figure S33 displays the alignment of representative members of this family with their conserved disulfide bonds.

Canonical lipocalins possess an N-terminal 3_10_-helix, a central eight-stranded anti-parallel β-barrel and a C-terminal α-helix [68]. The β-barrel may or may not possess a cavity that can be augmented by the inter β-strand loops to form a binding cavity. In some cases, the inter strand-loops allow formation of a second binding cavity near the opening of the barrel [69]. The N-terminal 3_10_-helix closes of one side of the barrel, while the C-terminal helix packs against the side of the barrel, giving the structure a distinct resemblance to a coffee cup [68]. Lipocalins may have 2–4 conserved disulfide bonds that stabilize the β-barrel and pin the C-terminal α-helix to the side of the barrel. The binding cavity allow tick lipocalins to scavenge a variety of bio-active molecules including biogenic amines, leukotrienes, and cholesterol [12,13,64,70,71,72,73]. Lipocalins also target complement C5 and properdin via protein–protein interactions and can modulate dendritic cell responses [74,75,76,77]. These functional properties allow ticks to modulate host defense responses such as inflammation, platelet aggregation, complement activation and immune responses.

For the current analysis, 175 lipocalin sequences were modelled with Alphafold2. Dali searches indicate that for the majority of proteins, the best targets are known tick lipocalins, with average Z-scores of 15 ± 3 (range of 2.8–23). The majority of models shows the core lipocalin fold, with deviations found for N- or C-terminal extensions. Pairwise comparison with the structure of the female histamine-binding protein HBP2 (1QFTA) indicated an average RMSD of 1.43 Å ± 0.15 Å. Some lipocalins presented as double lipocalin-domains. The high sequence diversity observed in the lipocalins preclude the construction of a single multiple sequence alignment. However, for the purposes of illustration a representative alignment of those lipocalins with known conserved functions are presented with some examples of structures and their bound ligands (Figure S34).

### 2.19. Kunitz-BPTI Family

The Kunitz-BPTI (basic pancreatic trypsin inhibitor) family is a ubiquitous family of serine protease inhibitors [78]. Ticks possess a large, expanded family of Kunitz-BPTI proteins that exist as single domain proteins, or multiple domain proteins ranging from two domains to more than seven Kunitz-BPTI domains linked in tandem [2]. Their basic single domain structure comprises an N-terminal 3_10_-helix, followed by an unordered strand that turn back to form a substrate-binding presenting loop, followed by a two-stranded anti-parallel β-sheet terminating in a C-terminal α-helix packed against the β-sheet. Generally, three conserved disulfide bonds stabilize the structure, the first between the 3_10_-helix and the C-terminal portion of the α-helix (C1–C6), the second at the start of the substrate-binding presenting loop linking to the turn that follows after the β-sheet (C2–C4) and the third linking the second β-sheet and the N-terminal portion of the α-helix (C3–C5) pinning the secondary structures together (Figure S35). In ticks, some BPTI proteins have been found that lack the second disulfide bond [79,80]. This presumably allows greater freedom of movement for the substrate-binding presenting loop and may have functional significance in those proteins that lacks this disulfide bond.

Members of this family mainly target serine proteases by binding to the enzyme active site via the substrate-binding presenting loop with additional interaction conferred by the C-terminal α-helix [78]. While some tick inhibitors would certainly use this mechanism to target serine proteases, significant deviations from this standard mode of inhibition is observed, notably that inhibitors from both hard and soft ticks insert their N-terminal residues into the active site of particularly fXa and thrombin, while interacting with substrate-binding exosites via their C-terminal α-helices [81,82,83]. In soft ticks, thrombin inhibitors are related to ornithodorin and seems to be evolutionary conserved in the Argasidae [84], while orthologs to boophilin are found in most metastriate ticks characterized to date [85,86]. Other Kunitz-BPTI inhibitors that target the blood clotting cascade include ixolaris and penthalaris that both target fX and fXa [79,87].

Inhibitors that target various receptors have also been characterized. This include integrins and ion channels [21,80,88]. The RGD integrin recognition motif is presented on the substrate-binding presenting loop of the savignygrins that target the platelet fibrinogen receptor α_IIb_β_3_ [21]. The RGD-motif may also be replaced by the RED motif that still allow targeting of the α_IIb_β_3_ receptor [84,88]. Kunitz-BPTI inhibitors with the RGD motif have been identified in all soft tick salivary gland transcriptomes sequenced to date [45,46,47,89].

For the current database 87 proteins have been modeled by Alphafold2. These included single domain Kunitz-BPTI proteins as well as three, four, five and seven domain proteins. These modeled structures correspond with manual assignment of the BPTI domains based on conserved disulfide bond structure. The double-domain proteins presented two types of structures, notably extended to produce a dumbbell shaped protein as observed for tick thrombin inhibitors [90], or two domains packed against each other to form a globular protein as observed in bikunin and ixolaris [91,92]. The modeling of multi-domain proteins (n ≥ 3) as observed for the models produced by Alphafold2 is a new and novel feature creating interesting possibilities to study the structure-function relationships of these molecules.

### 2.20. The 8.9 kDa Family

The 8.9 kDa family occurs exclusively in hard ticks and takes on a highly variable structure with most forms containing one or two domains. Also present are duplicated structures having two complete two-domain modules contained in a single polypeptide. Conserved cysteines are distributed throughout the structure with the simplest, most compact types having four cysteines (two disulfide bonds), with 6-, 8-cysteine proteins found in the two domain forms and 12- and 16 cysteines in the duplicated forms. Considerable variation in the disulfide bonding pattern of conserved cysteines is apparent. The common feature of the 8.9 kDa family is an N-terminal domain containing a four (or sometimes three)-stranded β-sheet having a Greek key topology [93]. The segment between strands 1 and 2 is extended and usually forms a 2- or 3-stranded antiparallel β-sheet which lies over the larger sheet, forming a sandwich. Most forms have a C-terminal domain of varying structure. The fold is like the type C domain of von Willebrand factor with most of the disulfide bonds being conserved [94]. Prostriate and metastriate genera contain two-domain, eight-cysteine types that resemble the C5-binding complement inhibitor CirpT1, but overall, the C-terminal domain is extremely variable and displays a number of different disulfide bond arrangements strongly suggesting that these proteins interact with many different targets and play a variety of biological roles.

#### 2.20.1. Taxonomic Distribution

##### Ixodes

The transcriptome of *I. ricinus* contains approximately 70 unique transcripts varying in identity from 14-80%. A large group of single domain peptides is present that are truncated at the C-terminal end of the N-terminal β-sheet structure (Figures S36A and S37A). These contain two disulfide bonds (four cysteines) with the first (DS1) linking the third strand from the larger β-sheet (β-sheet 1) with the second strand of the smaller sheet (β-sheet 2) and the second bond linking the loop between the second and third β-strands of β-sheet 1 with some part of the C-terminal region of the protein (DS-2). In this group of proteins β-strand A of β-sheet 1 is absent in the models and the N-terminus extends away from the protein and forms a short α-helical segment (Figure S37A). This group exhibits high overall amino acid sequence conservation ranging between 60 and 85% in amino acid identity.

Also present in *I. ricinus* is a larger group of peptides containing both the N- and C-terminal domains and having four disulfide bonds (eight cysteines). These are very diverse in amino acid sequence (17–70% identity) but are all similar in overall structure to the CirpT-type [93] complement inhibitors (Figures S36B and S37B). The N-terminal domain is much like the single-domain sequences discussed above. Many of these sequences are modeled by AlphaFold2 as lacking the first strand of β-sheet 1. A segment of unstructured sequence is present at the N-terminus in these cases suggesting that the full four-stranded β-sheet structure may be present (Figure S37B). The C-terminal domain of this group contains three disulfide bonds, most notably a cluster of two (DS2 and DS3) that is characteristic of all members of the 8.9 kDa family except those *Ixodes* variants that do not contain a C-terminal domain. This cluster contains two sequential cysteine residues, with the first linking to the loop between β-strands B and C of the N-terminal domain (DS2) and the second linking to the C- end of β-strand D of the N-terminal domain (DS3). A fourth disulfide bond (DS4) links the extreme C-terminus with a second C-terminal domain cysteine residue linking the strands forming a small two-stranded β-sheet (β-sheet 3) that is normally present in this domain (Figure S37B).

##### Amblyomma

Ticks in the genus *Amblyomma* produce a diverse set of peptides from the 8.9 kDa family containing variations in the structure of the C-terminal domain as well as forms in which the entire two domain structure is duplicated or partially duplicated to give proteins that could have multivalent binding properties. The *Amblyomma* forms contain 6, 8, 12 or 16 cysteine residues (3, 4, 6 or 8 disulfide bonds).

Six-cysteine forms from *Amblyomma* species are variable in sequence (20–80% amino acid sequence identity within the group) and contain the N-terminal domain seen in *Ixodes* proteins as well as a C-terminal domain that is truncated directly C-terminal to the “CC” double cysteine motif involved in DS2 and DS3 of the eight-cysteine forms described above (Figures S38A and S39A). Because of the shortened C-terminal domain in these forms, they do not contain DS4. Eight-cysteine variants are also present in *Amblyomma* which resemble those described in *Ixodes* and are quite diverse in sequence, showing about 20–80% amino acid identity within the group (Figures S38B and S39B). This group contains the C5 complement inhibitors and is similar in general structure to the eight-cysteine forms from *I. ricinus* (Figure S4B).

In addition to the six- and eight-cysteine forms of the 8.9 kDa family, *A. maculatum* (Kock, 1844) contains extended variants that have extra domains attached to the eight-cysteine structure. The simplest type has a three-domain structure resembling the four-cysteine, single domain forms described in *I. ricinus* attached to the C-terminal end of the eight-cysteine, two-domain module (Figures S40 and S41A). The chain continues from the N-terminal module into the extra domain forming a hairpin loop corresponding to β-sheet 2 of the *I. ricinus* single domain protein, then into a three-stranded, antiparallel β-sheet corresponding to β-sheet 1 of *I. ricinus* four-cysteine proteins and the eight-cysteine proteins from all species (Figure S41A). This type contains twelve cysteine residues forming six disulfide bonds which are conserved in position relative to those from the previously described proteins.

Also present in *A. maculatum* are variants with four domains made up of two complete eight-cysteine units fused end-to-end into a single polypeptide (Figures S40 and S41B). Like the three-domain forms described above, the second structural module of these proteins contains a modified N-terminal domain β-sheet structure, but also has a fully elaborated C-terminal domain containing a β-sheet 3 type structure (Figure S41B). These proteins contain sixteen conserved cysteine residues forming eight disulfide bonds.

##### Rhipicephalus

Like *Amblyomma*, *Rhipicephalus* (Kock, 1844) species contain 8.9 kDa family members having two domains with 6 or 8 cysteine residues as well as four-domain proteins containing 16 cysteine residues. An unusual group of 8-cysteine proteins with a shortened C-terminal domain is present. It contains an N-terminal domain with β-sheet 1 modeled as having three strands, and β-sheet 2 as having two or three strands (Figures S42A and S43A). The C-terminal domain is truncated relative to the 8-cysteine forms of *Ixodes* or *Amblyomma* but contains the “CC” double cysteine motif that makes up part of the two-disulfide bond cluster (Figure S43A). All members of this group are modeled by AlphaFold2 as having single free cysteines near the N-terminus of the protein and near the C-terminus, which are not in proximity to one another (Figure S43A). This suggests that they may form multimers or contain an unmodeled model structure in which these two unpaired cysteines are in proximity to form a disulfide bond.

A group of “conventional” 8-cysteine forms are also found in *Rhipicephalus* in which the domain structure and disulfide bonding pattern of most closely resembles comparable proteins from *Amblyomma* or *Ixodes*. This not to say they are highly similar at the sequence level or would be expected to be functionally homogeneous. Within this single species these proteins exhibit a range in amino acid identity of 17–52% (Figures S42B and S43B).

Finally, *R. appendiculatus* contains a set of extended variants like those of *Amblyomma* which contain four protein domains and sixteen cysteine residues. As in the *Amblyomma* forms, these are derived from the end-to-end fusion of two eight-cysteine forms into a single polypeptide. In this case, the N-terminus of the first eight-cysteine unit forms part of the C-terminal domain of the unit by forming one strand in in β-sheet 3, resulting in it having three strands rather than the usual two. In a second model, the C-terminus of the protein integrates into β-sheet 1 of the C-terminal eight-cysteine unit, making it a 4-stranded antiparallel sheet. The disulfide bonding pattern of this group is that expected from the fusion of two eight-cysteine units.

#### 2.20.2. C5-Binding Anticomplement Proteins

The only established function of the 8.9 kDa family is inhibition of complement by binding to component C5 and preventing its activation [93]. Orthologous 8-cysteine 8.9 kDa family members from *R. pulchellus* (Gerstäcker, 1873), *Dermacentor andersoni*, *R. sanguineus* and *A. americanum* have been found to function similarly. Using the crystal structure of CirpT1, the variant from *R. pulchellus,* complexed with the macroglobulin domain 7 of human complement C5 and the cryo electron microscopy structure of CirpT1 complexed with C5 along with the inhibitors OmCI and RaCI1, we identified a block of eight residues in the interaction interface (Figure S44). Of the selected set of sequences from the TickSialofam database, only those eight-cysteine forms closely resembling the previously described inhibitors contained even a small number of amino acid identities within the selected sequence block. Few partial matches or weakly similar sequences were found in the set of *Ixodes* transcripts suggesting that C5 inhibitors of this “type one” clade of the 8.9 kDa family and are restricted to metastriate species. As anticipated, due to the high variability and low degree of sequence identity within the groups described here, only orthologs of CirpT1 appear to have a C5 binding function. The structural diversity revealed by Alphafold2 modeling therefore suggests that the 8.9 kDa salivary protein family of the eight-cysteine type can be expected to perform multiple functions.

### 2.21. The 8-kDa Family

The 8-kDa family occurs in metastriate hard ticks and contain a cysteine knot structure characterized by a three- or four-stranded antiparallel β-sheet folded into an open sided barrel stabilized by three disulfide bonds (Figures S45 and S46) [95]. The disulfides are clustered at the end of the β-sheet containing both the N- and C-termini. Two alternative disulfide bonding patterns are seen, one with a pattern (based on relative cysteine positions) of: 1–6, 2–4, 3–5, and the second having a pattern of 1–4, 2–5, 3–6, which is also seen in the evasins. This family contains the RaCI complement inhibitors from *R. appendiculatus* which target complement factor C5 (Figures S45 and S46) [96]. These bind at a surface of C5 containing elements of the MG1, MG2 and C5d domains and prevent its cleavage by the C5 convertase. RaCI proteins from *R. appendiculatus*, *R. microplus* (Canestrini, 1888) and *D. andersoni* have been analyzed and found to act in a similar manner but have somewhat variable sequences in regions interacting with C5. This is explained by the large number of backbone interactions involved in C5 binding. RaCI peptides contain a lengthened loop between β-strands 1 and 2 that inserts into a pocket lying between the MG1 and MG2 domains of C5. This loop is not extended in other members of the family suggesting that these binding interactions cannot occur and that these proteins do not bind C5.

### 2.22. The 15-kDa Basic Family

The 15-kDa basic family found in *Amblyomma* sp. is a cysteine knot variant similar in structure to the 8-kDa family [95]. It contains an antiparallel β-sheet domain with two or three strands stabilized by three disulfide bonds in the pattern (based on relative cysteine positions) 1–4, 2–5, 3–6. Some members contain two additional cysteine residues forming a potential fourth disulfide bond as part of a disordered N-terminal coil (Figures S47 and S48). C-terminal to the cysteine knot domain is a length of disordered coil, followed by one or two helical segments containing greater than five turns which are then followed by a second intrinsically disordered region.

### 2.23. Complement-Binding Family

Members of the complement binding family are large proteins containing lectin or von Willebrand A (vWA) domains linked to strings of all β-sheet sushi-like domains, mostly stabilized by one or two disulfide bonds, that are reminiscent of complement control protein (CCP) domains. The lectin and vWA domains occur at the N-terminal end of the protein with the CCP domains extending out from them. One member of the group (JAR89651) from *I. ricinus* contains a fucose-binding lectin domain followed by a C-type lectin domain leading to ten repeated sushi domains. A second protein (JAR90946), also from *I. ricinus*, contains an N-terminal vWA domain followed by eight repeated modular domains Figures S49–S51). The proteins contain large numbers of cysteine residues, and all were predicted by AlphaFold2 to participate in disulfide bonds. These structures suggest that in blood feeding, the N-terminal parts may bind to exposed carbohydrate or collagen patches and the repeated domains may function as modulators of complement function in the mode of factor H. Other members that can be categorized as belonging to this group include JAB71472 from *I. ricinus* that contain only the repeated domains without the apparent lectin or vWA “anchors”. Interestingly, these proteins (see alignments of JAR89651 and JAR90946, Figures S49–S51) show high degrees of similarity to the N-terminal parts and complete conservation of cysteine residues of these large proteins from a wide variety of arthropods such as mites, crustaceans and horseshoe crabs that do not feed on vertebrate hosts suggesting that they have functions in endogenous systems not involving vertebrate blood such as immune surveillance. They are related to the sushi, von Willebrand factor type A, EGF, and pentraxin domain-containing (SVEP1) and CUB and sushi modular domain proteins (CSMD1) of vertebrates. CSMD1 is known to inhibit complement by a mechanism in which it serves as a cofactor to factor I in the degradation of C4b and C3b from the classical and alternative pathways of complement, respectively [97].

### 2.24. Dae-2 Family

The Dae-2 (domesticate amidase effector 2) proteins are a group of cysteine peptidases whose genes have been acquired by tick species by lateral transfer from bacterial genomes [98,99]. These serve an antimicrobial function by proteolytically cleaving cell wall peptidoglycan. The tick salivary Dae-2 proteins have been shown to have broader substrate specificity than microbial forms and are thought to act by controlling growth of skin microbes at the site of feeding. The catalytic cysteine and histidine residues (Cys 23 and His 73 in the bacterial Tae-2 numbering system) are conserved in all tick forms (Figure S52). Differences in surface structure, particularly along the substrate binding groove, are considered to be determinants of selectivity for peptidoglycans from different bacterial forms. Unlike the bacterial Tae-2 which contain no disulfide bonds, tick Dae-2 proteins contain a conserved disulfide linking Cys 58 and Cys 89 (in JAC30591 numbering, Figure S53). There is also a free cysteine at position 4 (JAC30591 numbering) and in most tick sequences this is paired with a cysteine at position 31 to form a second disulfide (Compare JAC30591 and AEO34830 in Supplementary Figures S52 and S53).

## 3. Methods

### 3.1. TSFam Database

From the TSFam database [5], 15,796 sequences obtained from tick salivary transcriptomes were selected based on the presence of a signal peptide indicative of secretion, and no transmembrane domains outside the signal peptide. The original database was clustered by blastp and paired-joining the sequences to attain 25, 30... 90, 95% identity in at least 70% of the longest sequence. Thus, for each degree of identity there are n clusters, sorted by their decreasing abundance. Accordingly, each particular cluster can be determined by two numbers, the first being the identity threshold and the second the cluster number for that identity (Supplemental spreadsheet S1). The sequences from each cluster were used to construct a psiblast generated PSSM, and these were combined, after proper annotation, within the database where tick sequences can be searched using the rpsblast tool (Supplementary TickSialoFam 2.0 database).

### 3.2. Alphafold2 Program

The Alphafold2 program [6] was run locally on the NIH Biowulf cluster in a Linux environment using 8 cpu’s, one v100x GPU and 60 GB RAM, using the monomer or multimer mode. Prediction of disulfide bonds were made by calculating the distances between all sulfur atoms from cysteine residues, available from the pdb files, and assigning a disulfide bond for those pairs that had a distance smaller than 3 Å.

### 3.3. Dali

The Dali program [7], available online: http://ekhidna2.biocenter.helsinki.fi/dali/README.v5.html (accessed on 1 July 2022) was used to compare the Alphafold2 predictions to the structures available in the PDB database. The program was run locally in the NIH Biowulf cluster. The program generates statistical analyses of the comparisons. According to the manual, a Z score above 20 indicates that two structures are homologous, between 8 and 20 that two structures are probably homologous, between 2 and 8 is a gray area, and a Z-score below 2 is not significant.

### 3.4. Disintegrin Searches

We have previously scanned salivary proteins from blood sucking arthropods for disintegrin motifs [19] after building prosite blocks (Supplementary File S1—Prosite disintegrin motifs) which were used to search the tick salivary protein database (Supplemental spreadsheet S1) using the program ps_can.pl [100] Available online: https://ftp.expasy.org/databases/prosite/.

## 4. Conclusions

The addition of structural fold prediction algorithms in the classification of secretory salivary gland protein families adds a powerful dimension that allows confirmation and validation of various protein families, groups or folds. It also allowed assignment of distantly related families or groups to well-known families or to predict novel folds not yet determined by conventional structural biological methodologies. The models provided by Alphafold2 also allow identification of potential homodimers and insights into the quaternary folds of proteins with multiple domains. In addition, the models provide insight into the potential functions and mechanisms of various families and provide a basis for assessment of structural integrity via disulphide bond predictions. Alphafold2-based classification as utilized for the TSFam2.0 database has already improved the original database, while adding significant information that can be used in hypothesis driven research on protein family function and evolution. The TSFam2.0 database is therefore a significant improvement on the original TSFam database that will with subsequent refinement and addition of more tick protein families and structures result in a comprehensive classification of tick protein families.

## Data Availability

Publicly available datasets were analyzed in this study. This data can be found here: https://proj-bip-prod-publicread.s3.amazonaws.com/transcriptome/TickSialoFam/TSF2.0/SupSpreadsheet+1.xlsx.

## Supplementary Materials

The following supporting information can be downloaded at: https://www.mdpi.com/article/10.3390/ijms232415613/s1, (1) Supplemental Figures: PowerPoint file with manuscript figures and movies. (2) Supplemental File S1-Disintegrin motifs in prosite format used to scan tick salivary proteins using the program ps_scan.pl Available online: https://github.com/ebi-pf-team/interproscan/blob/master/core/jms-implementation/support-mini-x86-32/bin/prosite/ps_scan.pl. (3) Supplemental spreadsheet S1–Hyperlinked spreadsheet containing putative tick salivary proteins linked to comparisons to several databases and AlphaFold predicted structures. Clusterization of the proteins allowed for extraction of reversed-position specific motifs collected into the TSFam 2.0 database. The spreadsheet has links to pdb files, which need programs that are able to open them. We suggest the use of ChimeraX Available online: (https://www.cgl.ucsf.edu/chimerax/download.html) or Swiss-PDBViewer Available online: https://spdbv.unil.ch/. (4) Supplementary TickSialoFam 2.0 database–Include RPS models and a formatted database which should be used to query protein sequences by means of the rpsblast program from the NCBI Blast suite of programs Available online: https://ftp.ncbi.nlm.nih.gov/blast/executables/blast+/LATEST/.
