# Supplementary material for: A Deeper Insight into the Tick Salivary Protein Families under the Light of Alphafold2 and Dali: Introducing the TickSialoFam 2.0 Database
Source: Int J Mol Sci. 2022 Dec 9;23(24):15613. doi: 10.3390/ijms232415613 (PMC9779611; doi:10.3390/ijms232415613)
Supplement: Supplementary file 1 [file ijms-23-15613-s001.zip › ijms-2052982-supplementary figures.pdf]

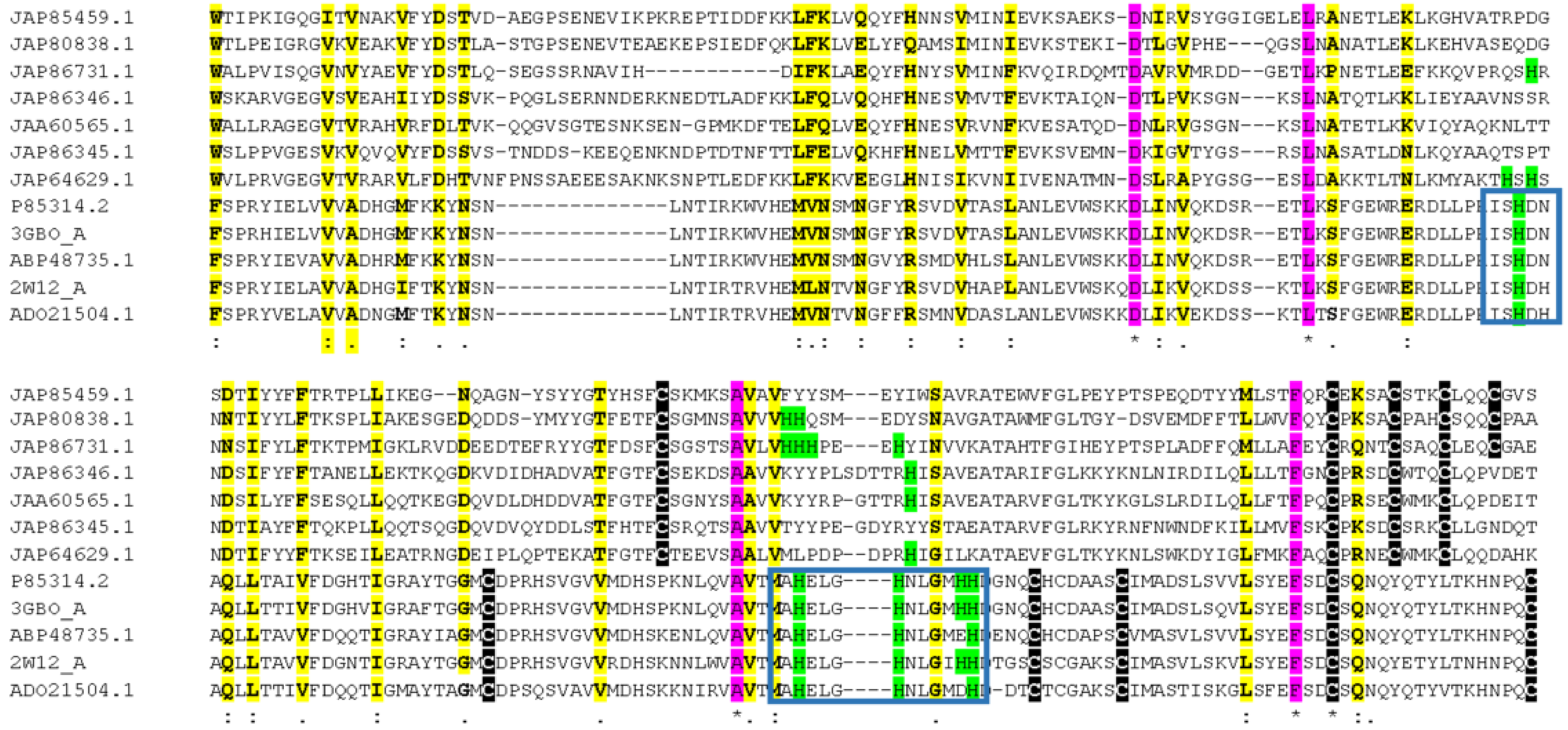

Figure S1: Alignment of members of the 28 kDa family with Zn metalloproteinases. The boxes show conserved histidines in green background. associated with the Zn binding function. The pink background displays identical amino acids, and in yellow the conserved sites. Cysteines are shown in black background. The sequences are named by their NCBI or PDB accessions.

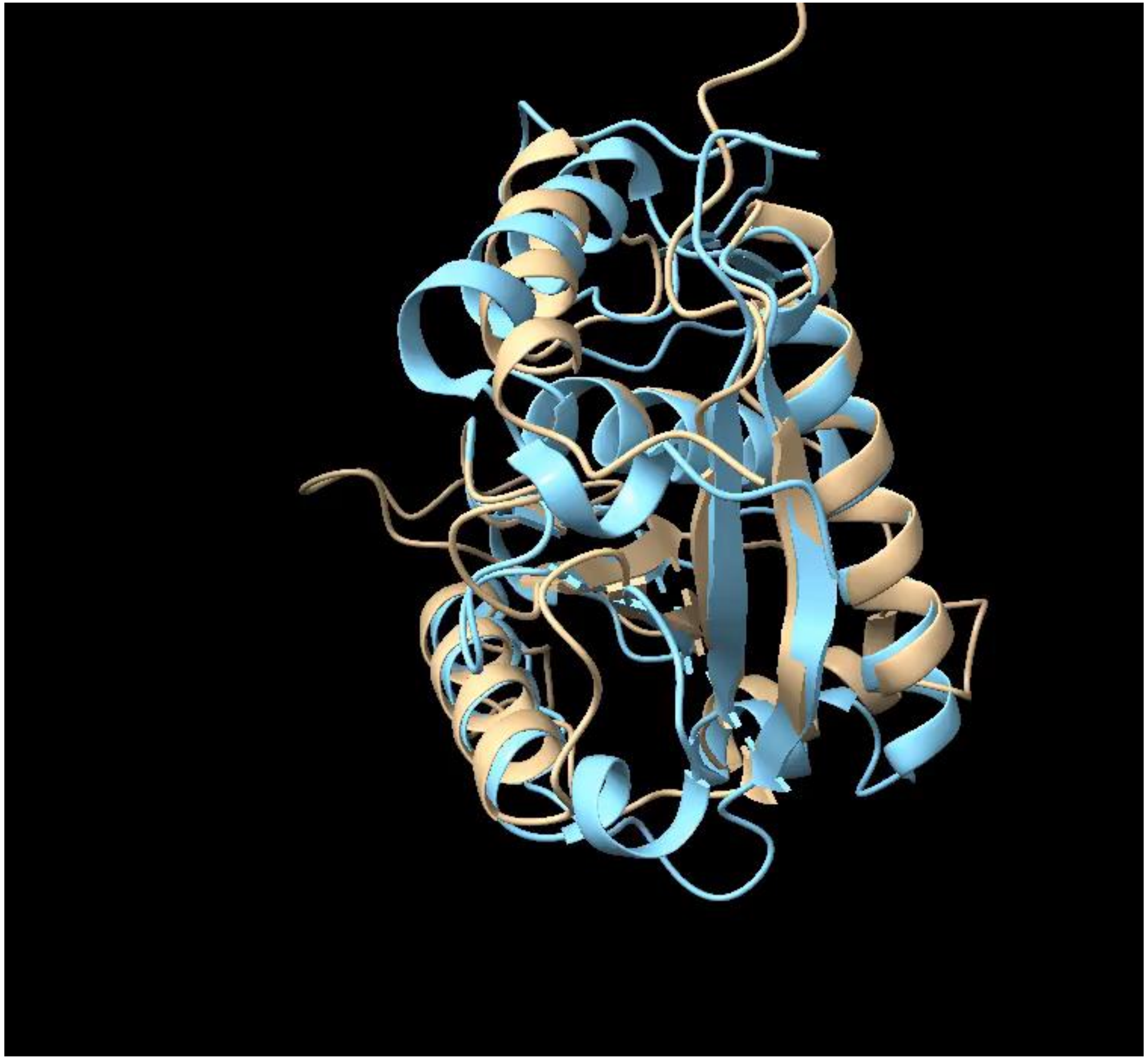

Figure S2: Metalloproteoid JAA55829.1 predicted Alphafold structure (Brown color) superimposed to the metalloprotease leucurolysin-a (PDB 4q1l-A) (Blue color).

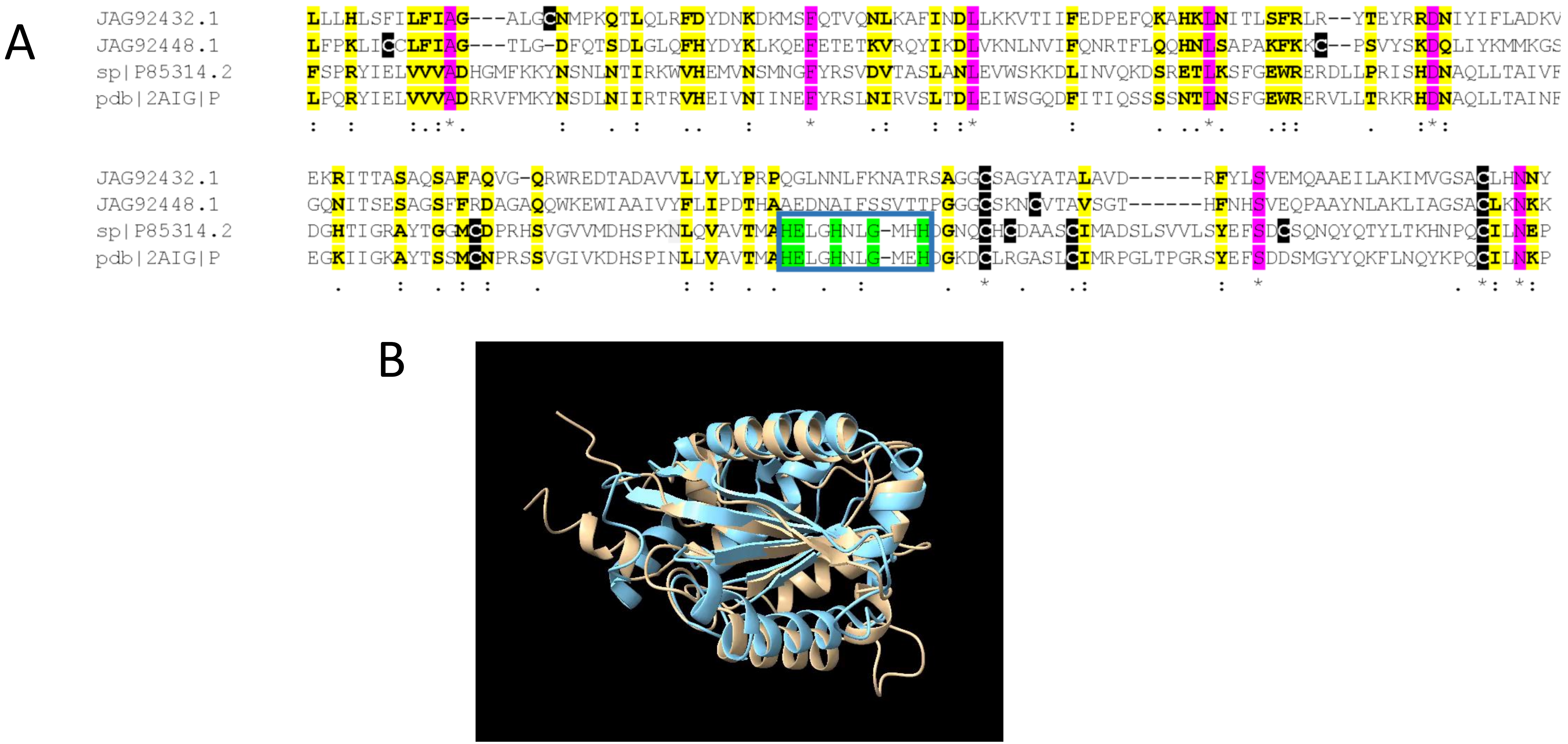

Figure S3: Metalloproteoid subgroup Amblyomma. A) Alignment of two tick protein sequences with two metalloproteases. B) Superimposition of the Alphafold structure prediction for JAG2448.1 (brown color) to the snake metalloprotease structure PDB:3gbo (Blue color).

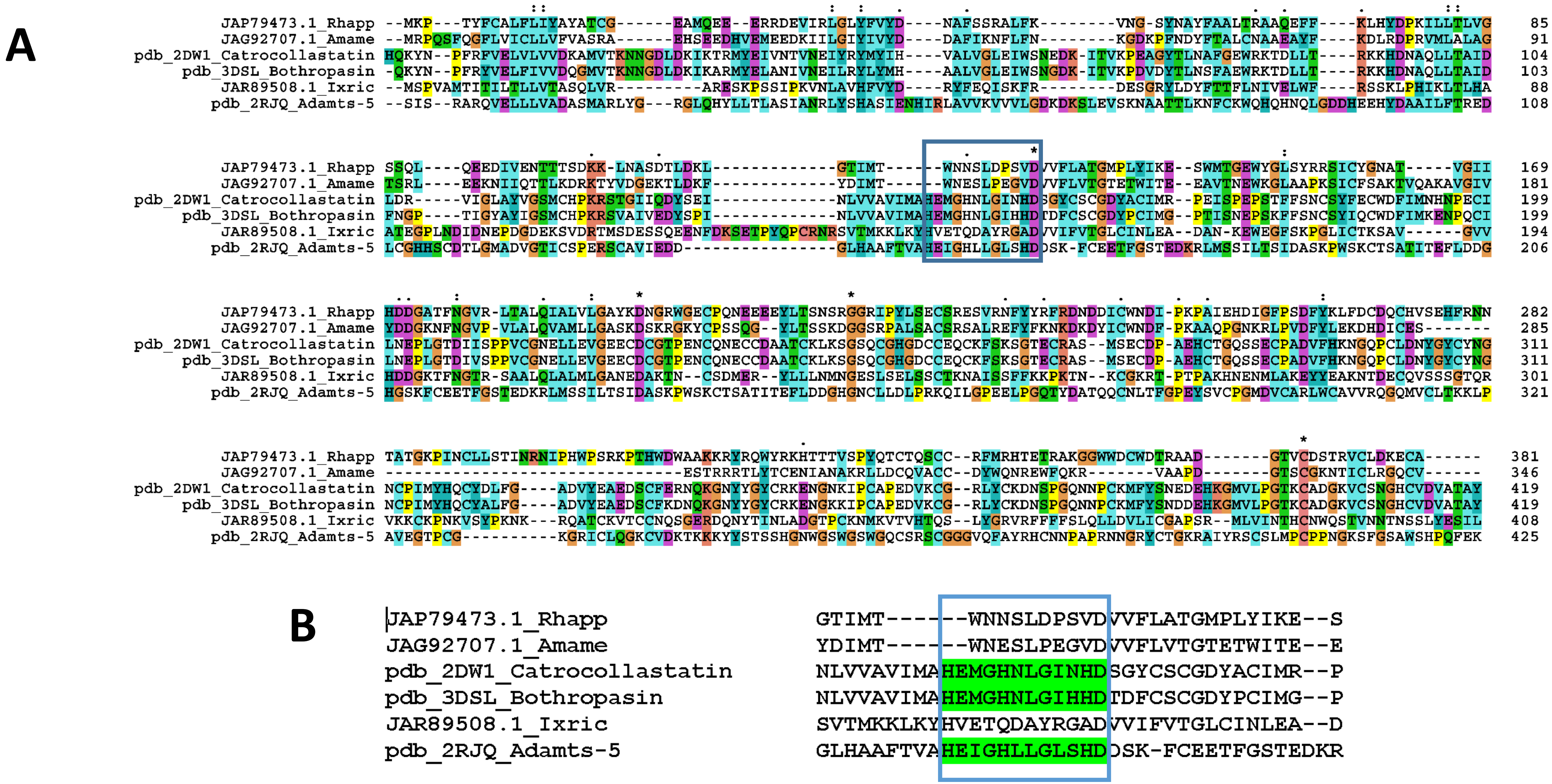

Figure S4: Alignment of three tick metalloproteoid protein sequences previously identified as metalloproteases, with three snake metalloproteases. A) Full ClustalW alignment. B) Detail of the alignment in the region of the Zn binding domain essential for catalytic activity. The boxes on the two figures delimit this domain. The snake proteases are indicated by their PDB accession, followed by their names. The tick sequences are identified by their NCBI accession, followed by the first two letters of their genus name and the first three letters of their species name.

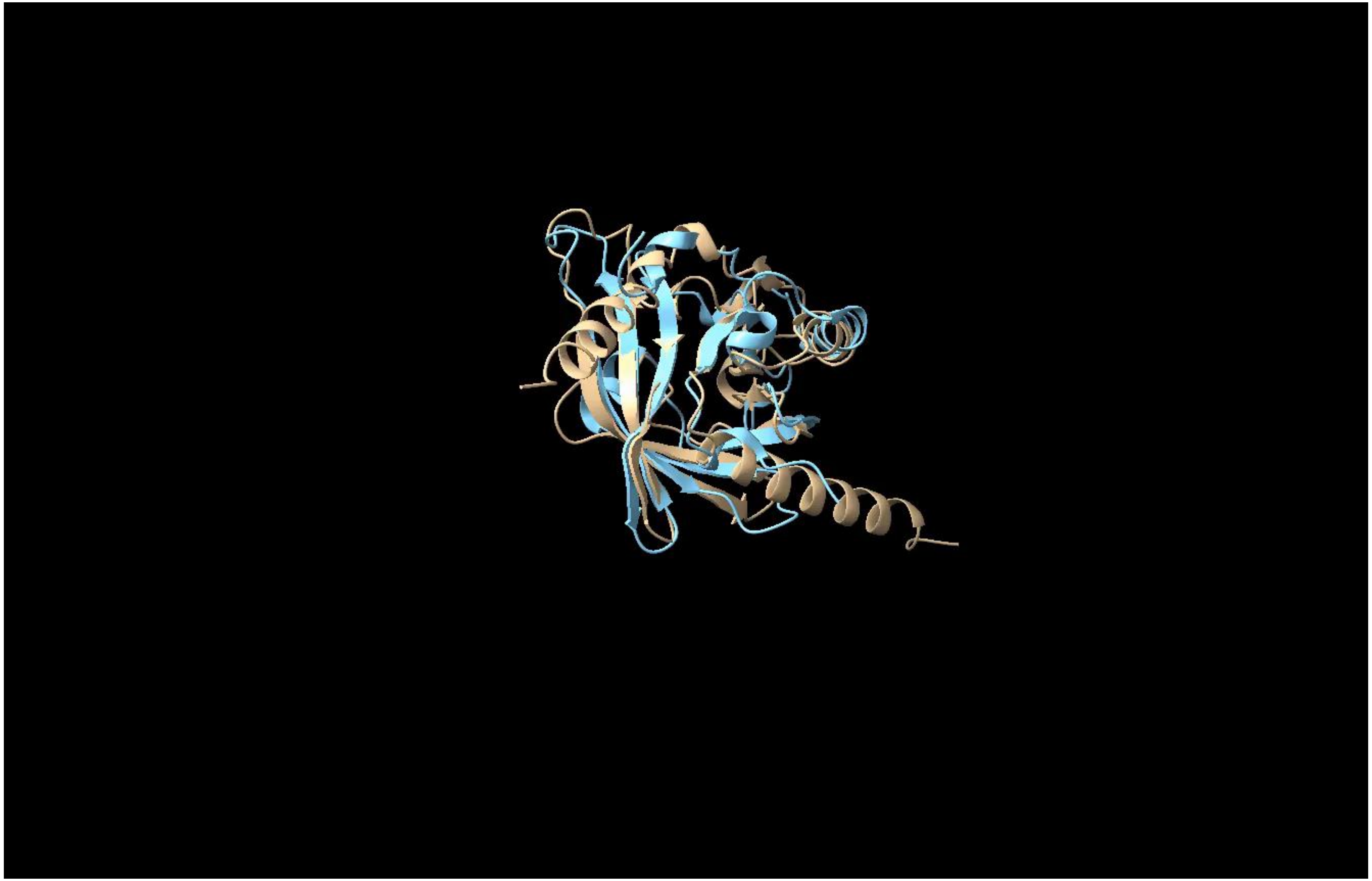

Figure S5: Superimposition of the Alphafold structure prediction for JAC28582.1 (brown color) to the lipocalin structure PDB:4boe (Japanin) (Blue color).

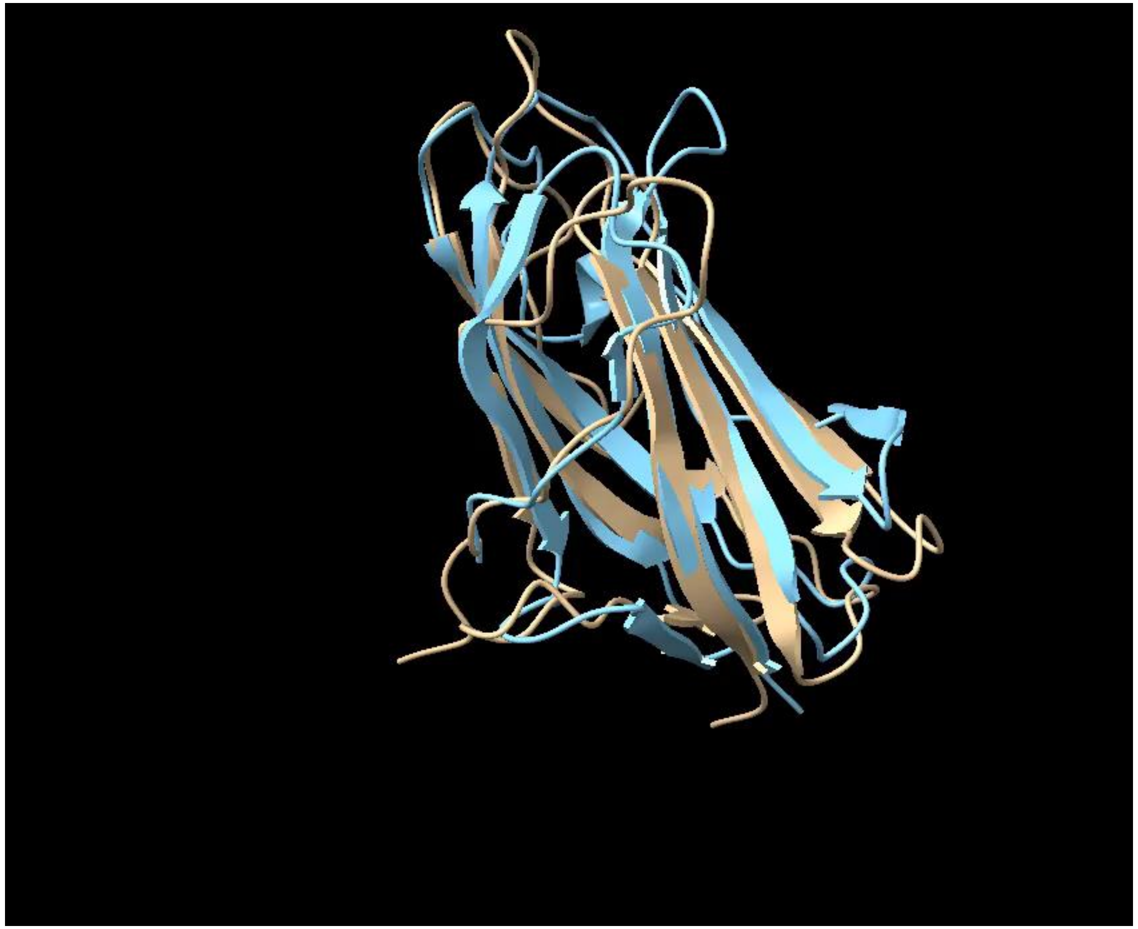

Figure S6: Former 17.7 kDa family matches Niemann-Pick proteins. Alphafold structure prediction for ABI52703.1 (brown color) superimposed to PDB:1nep-A (Bovine Niemann-Pick protein C2) (Blue color).

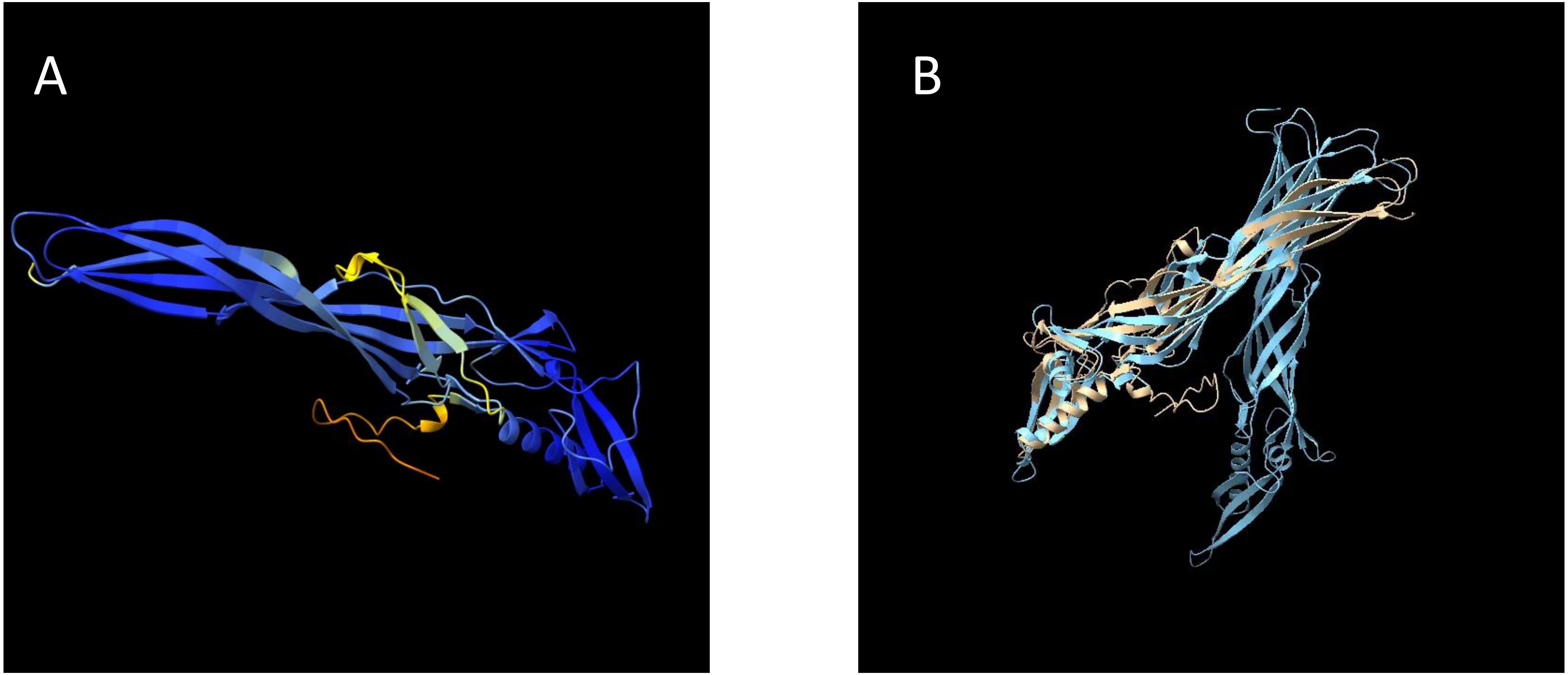

Figure S7: Tick Cytotoxin proteins: A) Alphafold predicted structure for *Ixodes ricinus* JAB71021.1. The ribbon color indicates the B factor confidence value where blue=highest and red the lower values. B) Comparison of JAB71021.1 with the dimeric bacterial protein PDB:2d42.

A

```
JAB72190_Ix_ric       DSIVSTVLPTNARSLG--LDSVDLPSYAFIVKSTS--LTNRDLHAEFHGGKLVGLVSPGLVRWGDCSAPGWQ--GF-N--VTLGCYLSLDNLHLSYVG
JAG92562_Amb_am       DGLFSTLVPLYARSLG--LENASVPNCAFNVASTS--VTNRDVNVELHGGRLLGLVIPGLARRGQCSAPGWL--GL-N--VTLGCYVSLDNAHLSYAG
JAC24281_Amb_caj      DLVLEQHLPPLVRGSHALYPGARIPDFHFKILKTG--ITNRDLKANISEGFIHGFDN-GVHRLGNCDPPMLI-DG--N--TTVNCVLNMTGIGATLTA
JAC26824_Amb_par      DLVLEQHLPPLVRGSHQLYPGARIPDFHFKILKTG--ITNRDLKANISEGFIHGFDE-GVHRLGNCEPPMLI-EG--N--TTVNCVLNMTGIGATLTA
JAA60568_Rh_pul       DVVLLQRMPALIRETPGLFPAAPLPPHYFKVYKTS--ITNRDLKVNVTRGGIRNFDT-AIHRVGDCIP-ALV-AG--N--TSVSCTLSFDGILAELVA
JAP85995_Rh_app       DVVLLQRMPALIRETPGLFPAAPLPPHYFKVYKTS--ITNRDLKVNVTRGGIRNFDT-AIHRVGDCIP-ALV-AG--N--TSVSCTLSFDGILAELVA
AEO36255_Amb_mac      DVVLLQRMPMLIRDTPGLYPTAPLPPFTFKVYKTA--ITNRDLKVNVTRGGIKNFDT-AVRRVGDCDP-KVV-AG--N--TSVTCVISFDGIASDMVA
JAC28147_Amb_tr       DFIIDQVLAGAG------VDVLDLPDISFDVDNQHNWTFSGYVEFHLQNGTLGNLRR-RVRRVGDCGLRGDQDAG--SPLVEIWCNLNLRGVVARYDV
pdb|3UV1|A            NKAIDDAIAAIEQSET--IDPXKVPDHADKFERHVG---ILDFKGELAXRNIEARGLKQXKRQGDANVKGEE--GIVK--AHLLIGVHDDIVSXEYDL
                      :  .   :.              :*     .           .. ..    :         * *:.        *  .  . :   :

JAB72190_Ix_ric       SAKGDSVLNTNKTLSLNVVPVKSSA-FIEVTSGS--GGIPSLKTWLIRPLNFSVG-VTKPLTLNNQRKTAFQSEIAKHSQAALLNVLLASFKEAVERS
JAG92562_Amb_am       SAKGDSLLNTNRSIVLQAMPVNASA-LLEVTSTP--GGAPRVRTWALQPFTLAVG-TSPKLSLNAARQATFDKEASKCVLSALSNILLVAYREALERS
JAC24281_Amb_caj      TTKGDSLVGTIKTIWVNVTLKKETVLRVGVTALA--GRTASLQTFFMEKLKLKTK-YDSHLSLNDDREDQFEELIEEKVRDILISAVYNPYKDLFARA
JAC26824_Amb_par      TTKGDSLVGTIKTIWVNVTLKKETVLRVGITALA--GRAASLQTFFMEKLKLKTK-YDSHLSLNDEREDQFEEHIEEKVRDILISTVYNPYKDLFARA
JAA60568_Rh_pul       VTKGDNLLNIVKSVDVEALVYNTTG-RLEVTAAP--NRPGFVRTLFVEGVNFDVR-PGSNLDLNEVRMNNFKTHIANFLREELYMNIYGNYQILLNHA
JAP85995_Rh_app       VTKGDNLLNIVKSVDVEALVYNTTG-RLEVTAAP--NRPGFVRTLFVEGVNFDVR-PGANLDLNEVRMNNFKTHIANFLREELYMNIYGNYQILLNHA
AEO36255_Amb_mac      VTKGDNLLGTIKDVGVQAVVYNTTG-TMEVTAAR--NRPGFVRTFFVEHVKLEVT-PGDNLDLNAVRMSNFKSYIANYLREELYLNLYGNYQTLLNYA
JAC28147_Amb_tr       EVIVD---DRSKSVVAELLVDTGNVYFKFESRQDNCTECSHFTDFQVTDVTARLKPLGVALDYAPDVIQQFENEVVSRAPKHISYAFTAAYKRALAKM
pdb|3UV1|A            AYKLGDLHPTTH-VISDIQDFVVALSLEIPDEGN--ITXTSFEVRQFANVVNHIGGLSILDPIFGVLSDVLTAIFQDTVRKEXTKVLAPAFKRELEKN
                          .      : :  :                        .    .  .                    :     .         .   ::  .
```

B

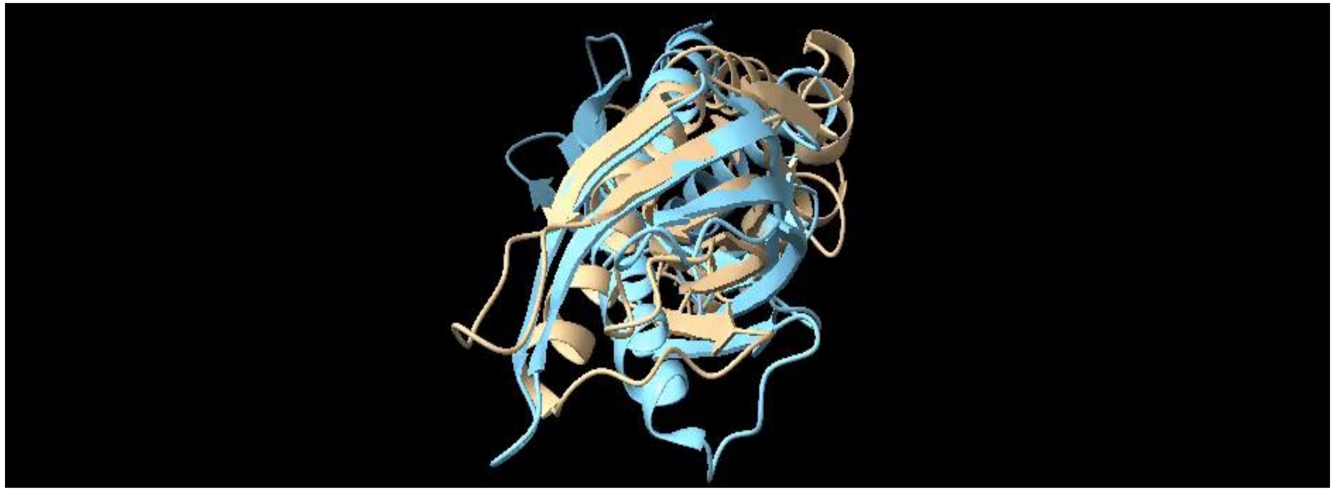

Figure S8: A subset of formerly assigned Cytotoxin proteins are most similar to Der f 7 allergen. A) Clustal alignment of tick salivary proteins with PDB:3uv1-A (Der f 7 allergen). Cysteines are shown in black background. Identical and similar amino acids are shown in green background. B) Superimposition of the Alphafold predicted structure of JAB72190 to PDB:3uv1-A.

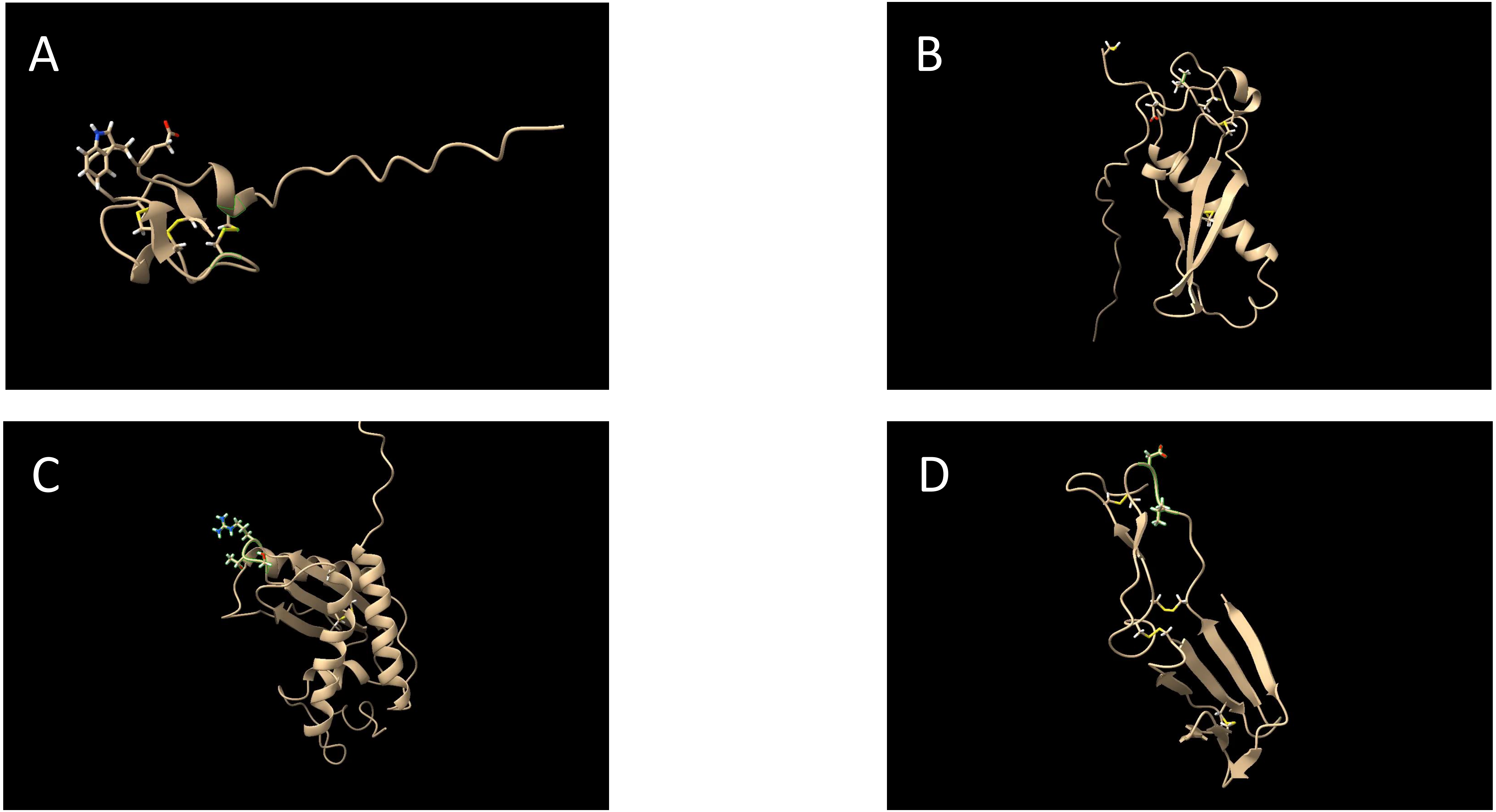

Figure S9: Tick salivary proteins with disintegrin motifs. A) 5.3 kDa AAY66640.1 with WGD domain.   B) 8.9 kDa AEO35167.1 with VGD domain. C) Antigen-5 JAA67471.1 with RTS domain. D) Salp15 ABI97200.1 with VGD domain. Disulfide bonds are shown in yellow.

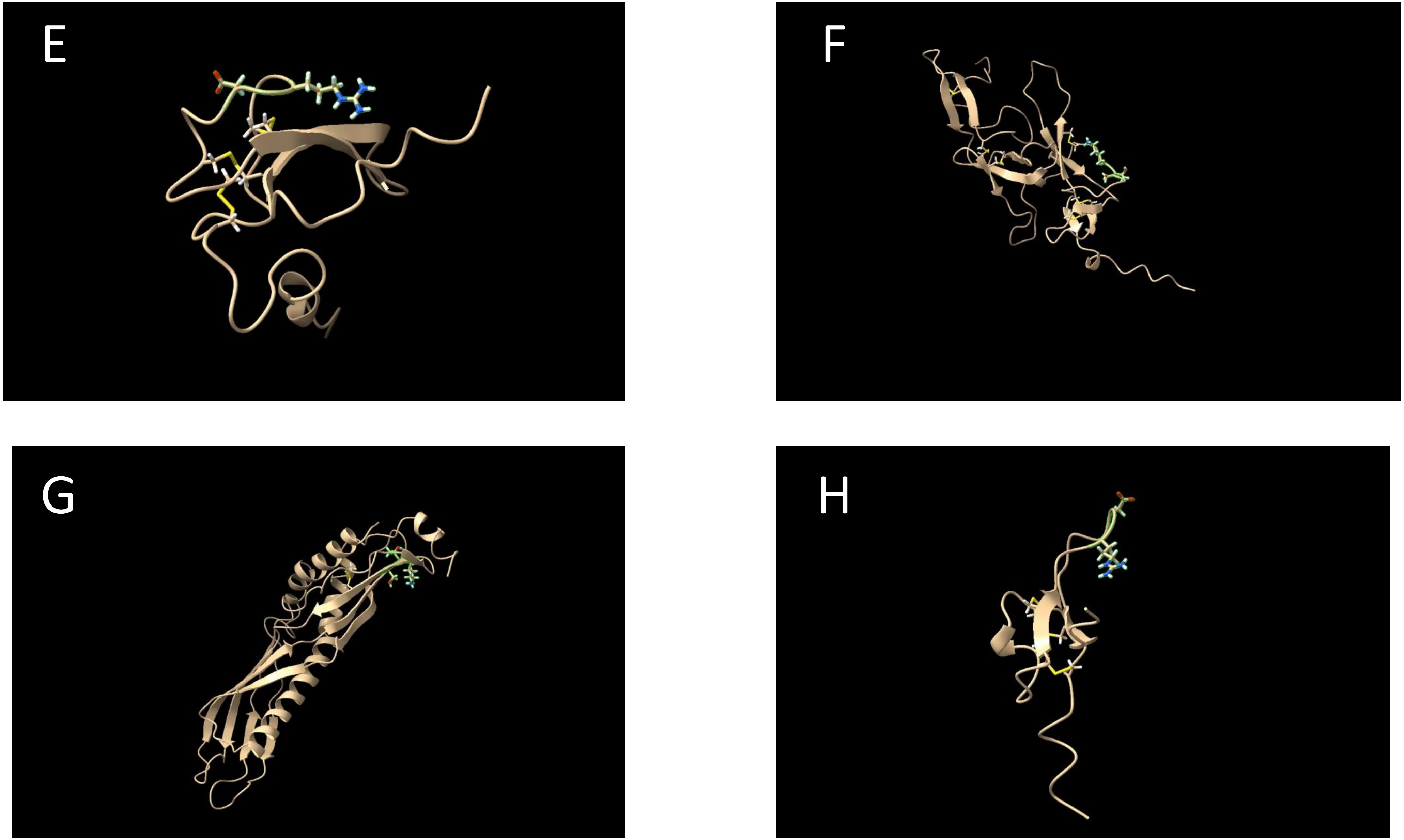

Figure S9: (Continuation): Tick salivary proteins with disintegrin motifs. E) Defensin JAP78020.1 with RGD domain. F) Basic Tail JAR94714.1 with KTS domain. G) Derf7/JHBP JAB71528.1 with KTS domain. H) Ixodegrin JAA69873.1 with RGD domain. Disulfide bonds are shown in yellow

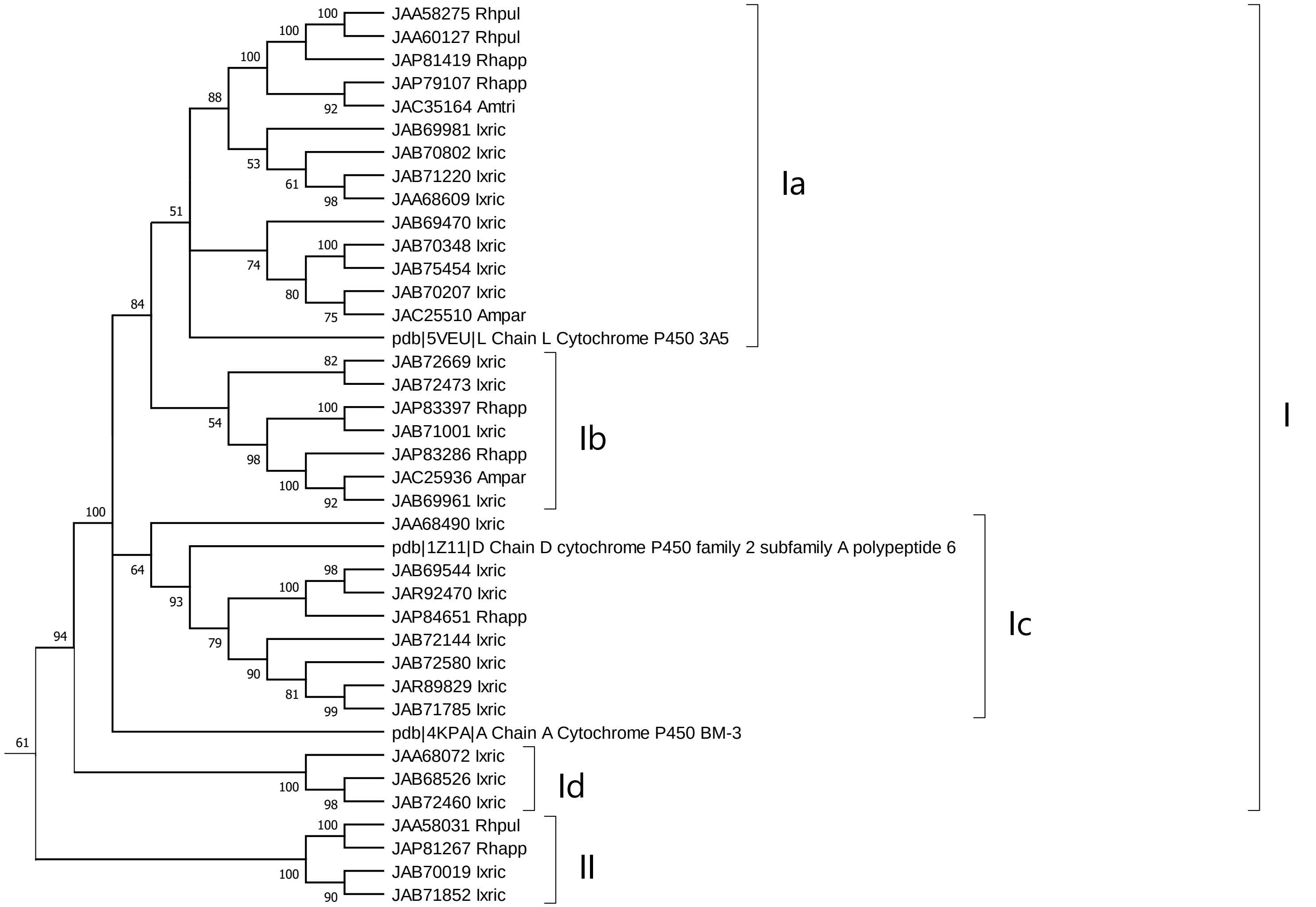

Figure S10: The tick salivary secreted cytochrome P450 family of proteins. Evolutionary analysis by Maximum Likelihood method. The evolutionary history was inferred by using the Maximum Likelihood method and JTT matrix-based model [1]. The bootstrap consensus tree inferred from 500 replicates [3] is taken to represent the evolutionary history of the taxa analyzed [3]. Branches corresponding to partitions reproduced in less than 50% bootstrap replicates are collapsed. The percentage of replicate trees in which the associated taxa clustered together in the bootstrap test (500 replicates) are shown next to the branches [3]. Initial tree(s) for the heuristic search were obtained automatically by applying Neighbor-Join and BioNJ algorithms to a matrix of pairwise distances estimated using the JTT model, and then selecting the topology with superior log likelihood value. This analysis involved 42 amino acid sequences. All positions with less than 50% site coverage were eliminated, i.e., fewer than 50% alignment gaps, missing data, and ambiguous bases were allowed at any position (partial deletion option). There were a total of 492 positions in the final dataset. Evolutionary analyses were conducted in MEGA X [2]

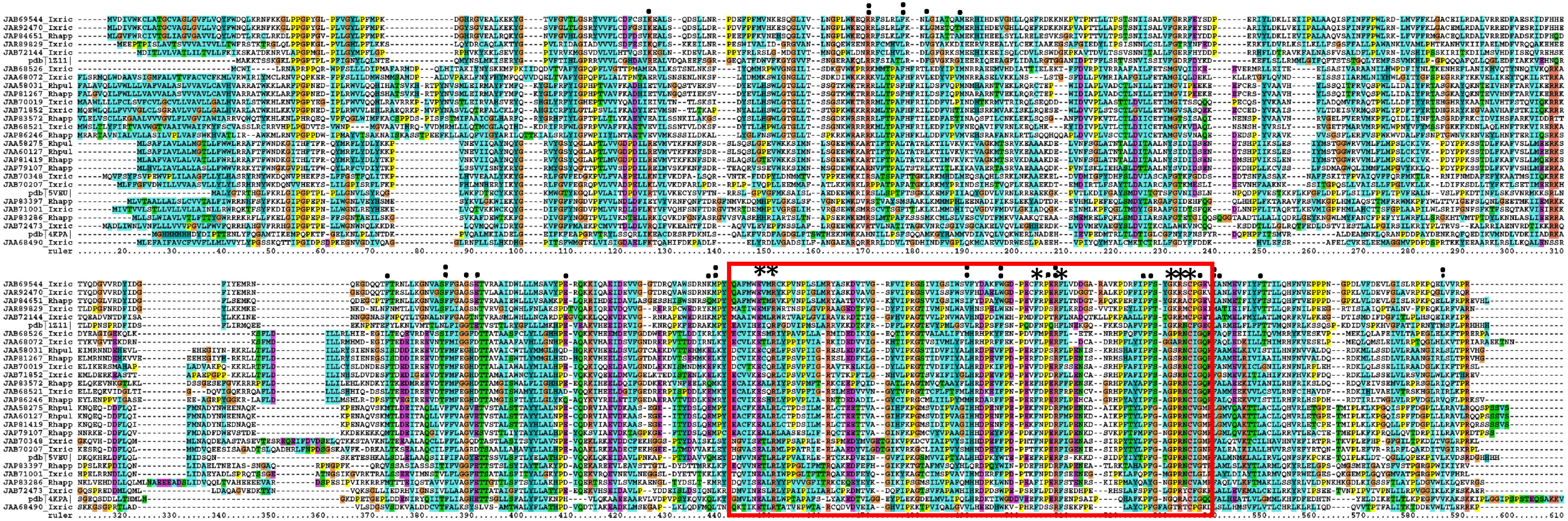

Figure S11A: ClustalW alignment of secreted tick salivary proteins of the cytochrome p450 family together with matching sequences from the PDB database. The red box indicates the region **c**ontaining the P450 signature amino acid sequence of the enzyme active center FxxGx(H/R)xCxG (including the Cys that coordinates with the heme iron) and the signature [AG]-G-x-[ED]-T that contacts the heme moiety.

```
JAB70348_Ixric     LTEEAALAQCLLFFLAGQDTASTLASITSYVLAVNPE-VQEKLRKEVDDCFEKHGGS-PTYDAISKLSYLNGVISETLRMFPSAPRLE-RSPMEDYMVGETGIKVPKDCVIAIPVYSIHHDPEFFPD-PHTFNPERFIGENAS--SIRPYTYLPFGAGPRNCIGMRFALQTVKTCILHAVHNVEFIRTPR
JAB70207_Ixric     LTKSEALAQCILFFLAGRDTSAALLAHAVYLLALHPE-AQAKLRKEADECFEKHGEE-LTMDVVAKLEYVHGVASEALRMLPPATRIE-RLAAEDYVLGDTGIKVRKGCLIAIPVYSMHHDPEYFPD-PFTFKPERFIGENIE--SIRPYTYLPFGAGPRNCIGNRFALEAIKIALLHVVRSVEFYRTQN
pdb|5VEU|          LSDLELAAQSIIFIFAGYETTSSVLSFTLYELATHPD-VQQKLQKEIDAVLPNKAP--PTYDAVVQMEYLDMVVNETLRLFPVAIRLE-RTCKKDVEIN--GVFIPKGSMVVIPTYALHHDPKYWTE-PEEFRPERFS-KKKD--SIDPYIYTPFGTGPRNCIGMRFALMNMKLALIRVLQNFSFKPCKE
JAA58275_Rhpul     MTLDEITAQLLVFFVAGVETVSTALSTTAYYLALNPE-CQDRVIAEVDKAAS-EGE--ITYDSLQEMPYLEACFKEALRLCTPDSILM-RLCTEETTVA--GIHFKPGMSVDIPVAGIHHDPENFPE-PEKFNPDRFMPENKD--AIKPFTYLPFGAGPRNCVGMRLGMVQAKTTLACLLQHVRLETGPE
JAA60127_Rhpul     MTLDEITAQLLVFFVAGVETVSTALSTTAYYLALNPE-CQDRVIAEVDKAAS-EGE--ITYDSLQEMPYLEACFKEALRLCTPDSILM-RLCTEETTVA--GIHFKPGMSVDIPVAGIHHDPENFPE-PEKFNPDRFMPENKD--AIKPFTYLPFGAGPRNCVGMRLGMVQAKTTLACLLQHVRLETGPE
JAP81419_Rhapp     MTLDEITAQLLVFFVAGVETVSTALSTTAYHLALNPE-CQDRVIAEVDKAAS-EGE--ITYDSLQEMPYLEACFKEALRLCTPDSILM-RLCTEETTVA--GIHFKPGMSIDIPVAGIHHDPENFPE-PEKFNPDRFLPENKD--AIKPFTYLPFGAGPRNCVGMRLGMVQAKTTLACLLQHVRLETCPE
JAP79107_Rhapp     MTLDEITAQGIVFFLAGVESVSTTLIYTAYYLALHPE-YQKSVIAEVDKTAGPKGE--FTYDSLQEMPCLEACIKEALRLAASESLIF-RQCTEETTVA--GINFKPGMCVYVPAAGIHHDPEYFPE-PDKFNPERFLPENKD--SVKPFTFMPFGNGPRNCVGMRLGMLQVKTTLACLLRRVTLEACTE
JAP83397_Rhapp     VSSASIASSSITIFVGGFETTSTSLSYIAYTLAKYPD-AQEKAREEVLEAVSTTGTL-DYDTTMRKLKFLEQVVNETLRLYPPGLIFMTRQAKEDFEFH--GIKFKTGTAFMVSQYHLQRDPQYWIN-PDEFHPDRFAPENEA--TLRKTAHAPFGLGPRNCVGTRLAMLSLKYTLARMLQKYRLELGPS
JAB71001_Ixric     LSSEEVIMNSTVLFLGGFETSATALSFITYALGKYPD-VQEKVRQEVNEVLNEGGSL-DYETVTKKLKYVTQVINEALRIWPPGLTFTTRQAKEDFEYQ--GIKYKAGTCIMSPVAVIHMDERFFPD-PTKFNPDRFSEENEG--SIPKLAFQPFGMGPRNCLGLRLAYLDIAYTIARMVQNFRWELGES
JAP83286_Rhapp     MTTTEIQSTAVVFLVAGFETTGTTLSFTSYLLAKHPD-IQERTRSEVLSVMKAENGL-TYDSLT-KMRYLDQVISEALRYYPVVVGFITRKCEQEYEYK--GIKIPSGMSILVPAFQMHHDPKLWND-PEAFIPERFSTENRS--KIEPMAYQAYGNGPRNCVAMRFAQLVLKFTMAKLLSSYRLVLDPN
JAB68526_Ixric     LTEQEVRDEVSSIFIGGFDTTATAASFCLYLLGHHPE-VQKKVHEEMDEVFGDDWERPVTLDDIKRLKYTECVIKESMRIYPAVPLLA-RMIDEDIKIG--EQTIPKGTVALALIFYMHRHPKYFEN-PDVFMPERFL--ETK--NRHPFQFVPFSAGPRNCIGQKFAQFEEIILLTQVMRRYKVESKVP
JAA68072_Ixric     FTEDEIREEVNTFMIGGFDTTATAAAFAVHLLGNHPE-AQAKVHAEIDSVFGNDRERPVTTDDIRNLKYLECVLKETLRLYPPIPVIA-RKIDEDVVIG--KHTIPKGTVSIVMLYFLHRHPRFFEK-PDVFLPERFL--DCA--DRHPFLYIPFSGGARNCIGQKFAQLEDKILLTHIMRHFKVESELP
JAA58031_Rhpul     ISDDDIREEVDTFMFEGHDTTAVAICWTLYMMGLHQD-HQRKVHEELDSVLGTCAEKDVTTEHMKELKYLDCVIKECQRLFPSVPIIG-RESLEDFKLG--DYVIPKGSTIDVFIYALHRDPEVFPD-PERFDPSRFLPENIS--KRHSHAFIPFSAGSRNCIGQRFAAMELKIVISTILHNFNVVALDQ
JAP81267_Rhapp     ISDDDIREEVDTFMFEGHDTTAVAISWTLYMMGLHED-HQRKVHEELDSVLGTDAEKDVTSEHMKELKYLDCVIKECQRLFPSVPIIG-RESLEDFKLG--DYVIPKGSTIDVFIYALHRDPEVFPD-PERFDPSRFLPENIS--NRHSHAFIPFSAGSRNCIGQRFAAMELRIVIAAILHNFNVVALDQ
JAB70019_Ixric     FTDEDIREEVDTFMFEGHDTTALAIAWALYMIALHPE-IQQKIQQELDAVLGNDLEKNISMNDMKEFKYLDCVTKECQRLYPSVPFIG-RAVTKELKLG--DYLLPEGTTVDIFIYALHRDPKIFPD-PEVFDPERFSSENSA--SRHPFAFIPFSAGSRNCIGQKFANMEVKICLGNILRNFNLRPLEH
JAB71852_Ixric     FTDEDIGEEVDTFMFAGHDTTAMAIAWNCYLIALHPD-VQKKVQEELDMVLGELKTEDISTENLKELKYLECVVKESQRLCPSVPVIG-RTVTKPFTLG--NHVLPEGTSVEIFIYGLHRDPEVFPD-PEVFDPDRFLLENCA--SRHPFAFIPFSAGSRNCIGQRFGSMEVKITIAHIFKNFHVKPFDQ
JAP83572_Rhapp     ITKEDMREEVDTFMFGGHDTTAMGISWALFLIGHHPE-EQRKIHEELDQIFGDDKERYVNFEELRQMKYLECAIKEALRIYPSVPMFT-RKCEEPFQID--GATLPAGTMVQTAAYFLHRDPEVFPK-PEEFHPERFFPENSK--GRHPFAYAPFSAGPRNCIGQKFALAEEKIVIANILRHFRIKSLDQ
JAB68521_Ixric     LTEEDIREEVDTFMFEGHDTTAMGISWAMYLIGLHAD-VQQKIHEELDGIFGEDRERPISPDDLKEMKYLECALKESQRLFPSVPFIG-RELMEDVVVN--GYTVPRGTTCFLFTFMLHRDKEIFPN-PEVFDPERFRPENCV--GRHPFAYVPFSAGPRNCIGQKFAMMEEKVVLCSVLRNFCIHAVDF
JAP86246_Rhapp     FTVEGIREEVDTFMFEGHDTTAMGISFTLYLLGLYKQ-AQYKVYRELETIFGEDAERAVTVDDLKEMRYLECVIKESHRLYPPVPLIA-RNTDEDADIL--GYCIPRGCMLIILPYMMHRDARFFPE-PEEFRPERFLPEHCA--GRHPYAYIPFSAGPRNCIGQRFALQEEKVVISTILRHFRLHSPDH
JAB69544_Ixric     FTRNLLKGNVASFFGAGSETVRAAIDWLLLMSAVYPE-RQKKIQAEIDEVVG-GTDRQVAWSDRNKMPYTQAFMWEVMRCKPVNPLSLMRYASKDVTVG--RFVIPEGSIVIGSIWSVFYDAKFWGD-PECFRPERFLVDDGA-RAVKPDRFIPFSYGKRSCPGEVIANMEVFIYFTTILQHFNVEPPPN
JAR92470_Ixric     FTRSLLKGNVGSFFGAGSETVLAAIDWLLLMSAVYPE-RQKKIQAEIDEVVG-GTDRQVAWSDRNKMPYTQAFMWEVMRYKPVSPIGLMRYASKDVAVG--SFVIPEGSVVISSFWSVFHDAELWGD-PECFRPERFLVDGGT-RALKPDFFIPFSYGKRSCPGKVIANLEVFIYFTTLLQHFNVEPPPN
JAP84651_Rhapp     FTRNLLKGNVASFFGAGSETVRAAIEWLLLVSAMKPE-LQRRIHDEIDAVLASGESSHISWSNRSQMPYTQAFMWETMRVKPVNPLSLMRYAATDVKVG--KYVIPRGSVVIGSIWSIFYDPAYWGD-PEVFRPERFLTNDGT-RPQKPERFIPFSYGKRACPGEVIANMETFIYFTSILQHFTVEPPPD
JAR89829_Ixric     FNMTLLVGNVLSFLIAGSNTVRVGIQWHLFNLAKSPDTVQARIQREVDQVVG--RERRPCWEDRYKMPYTMATIWEMFRWRTNSPLGVPRAAGEDTFFG--DYFIPKGTIVMPNLWAVHNSPELWEN-PEVFDPSRFLKRDGS-VVTKPEYLIPFTIGKRMCPGEIMATIEIFLYVTTLLQRFNILPEEG
JAB72144_Ixric     FNPQTLTGNALNFFGAGTNTVRASMLWHLLNCARDPEGVQKKLQQEVDKVVG--RNRAPEWEDRHKMPYTMAAIWEMLRWRTVAPLGVIRAVERDTTIG--GFHVPAGTVVLPNVWAVHNDPDIWEH-PMDFDPTRFLNADQTKLLPKPAALLPFSTGRRMCPGETLATMELFMYLATLMQRFRVLPKEG
pdb|1Z11|          FYLKNLVMTTLNLFIGGTETVSTTLRYGFLLLMKHPE-VEAKVHEEIDRVIG--KNRQPKFEDRAKMPYMEAVIHEIQRFGDVIPMSLARRVKKDTKFR--DFFLPKGTEVYPMLGSVLRDPSFFSN-PQDFNPQHFLNEKGQ--FKKSDAFVPFSIGKRNCFGEGLARMELFLFFTTVMQNFRLKSSQS
JAB72473_Ixric     IEDRHVIGNSIVLLAAGFETTATAIGFTLYLLATYPE-EQEKVHSEIDAVLP--YDDEISLDSIQNLKRLDMVINESLRLYPAIPILIARLCPRDMTVK--DQFIPAGTSVFAPAWHIHRDPDSWPE-PSRFLPDRFSEDHPE---RHSLAFSPFGLGPRYCFGKRLALLEVKMALCEILRKYRVIPSEE
pdb|4KPA|          LDDENIRYQIITFLIAGHETTSGLLSFALYFLVKNPH----VLQKAAEEAARVLVDPVPSYKQVKQLKYVGMVLNEALRLWPTAPAFS-LYAKEDTVLGG-EYPLEKGDELMVLIPQLHRDKTIWGDDVEEFRPERFENPSAIP----QHAFKPFGNGQRACIGQQFALHEATLVLGMMLKHFDFEDHTN
                   .           ::  * ::                 .                            .:        *  *                            .         :      : .    * * :*                 .:  * * * . :.       .   .
```

Figure S11 B: ClustalW alignment of secreted tick salivary proteins of the cytochrome p450 family together with matching sequences from the PDB database. The green-labelled amino acids represent the signature [AG]-G-x-[ED]-T that contacts the heme moiety; the pink-labelled amino acids represent the active center signature sequence FxxGx(H/R)xCxG (including the Cys that coordinates with the heme iron).

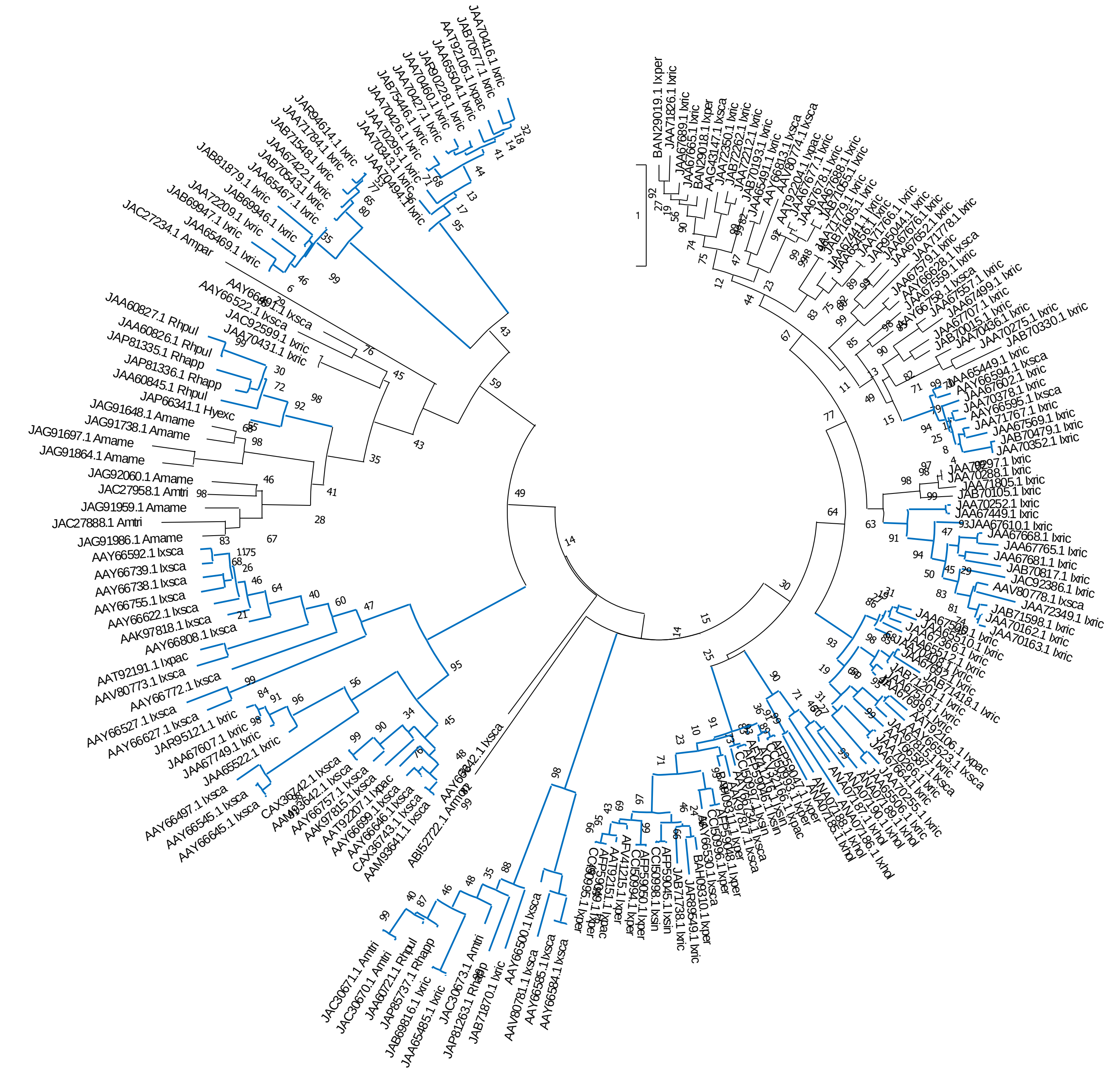

Figure S12: Phylogenetic analysis of the Salp15-Ixostatin group. The evolutionary history was inferred by using the Maximum Likelihood method and JTT matrix-based model1. The bootstrap consensus tree inferred from 500 replicates is taken to represent the evolutionary history of the taxa analyzed. Branches corresponding to partitions reproduced in less than 50% bootstrap replicates are collapsed. The percentage of replicate trees in which the associated taxa clustered together in the bootstrap test (500 replicates) are shown next to the branches. Initial tree(s) for the heuristic search were obtained automatically by applying Neighbor-Join and BioNJ algorithms to a matrix of pairwise distances estimated using the JTT model, and then selecting the topology with superior log likelihood value. This analysis involved 197 amino acid sequences. All positions with less than 50% site coverage were eliminated, i.e., fewer than 50% alignment gaps, missing data, and ambiguous bases were allowed at any position (partial deletion option). There were a total of 148 positions in the final dataset. Evolutionary analyses were conducted in MEGA X. Branches with bootstrap support larger than 90% are shown in blue color.

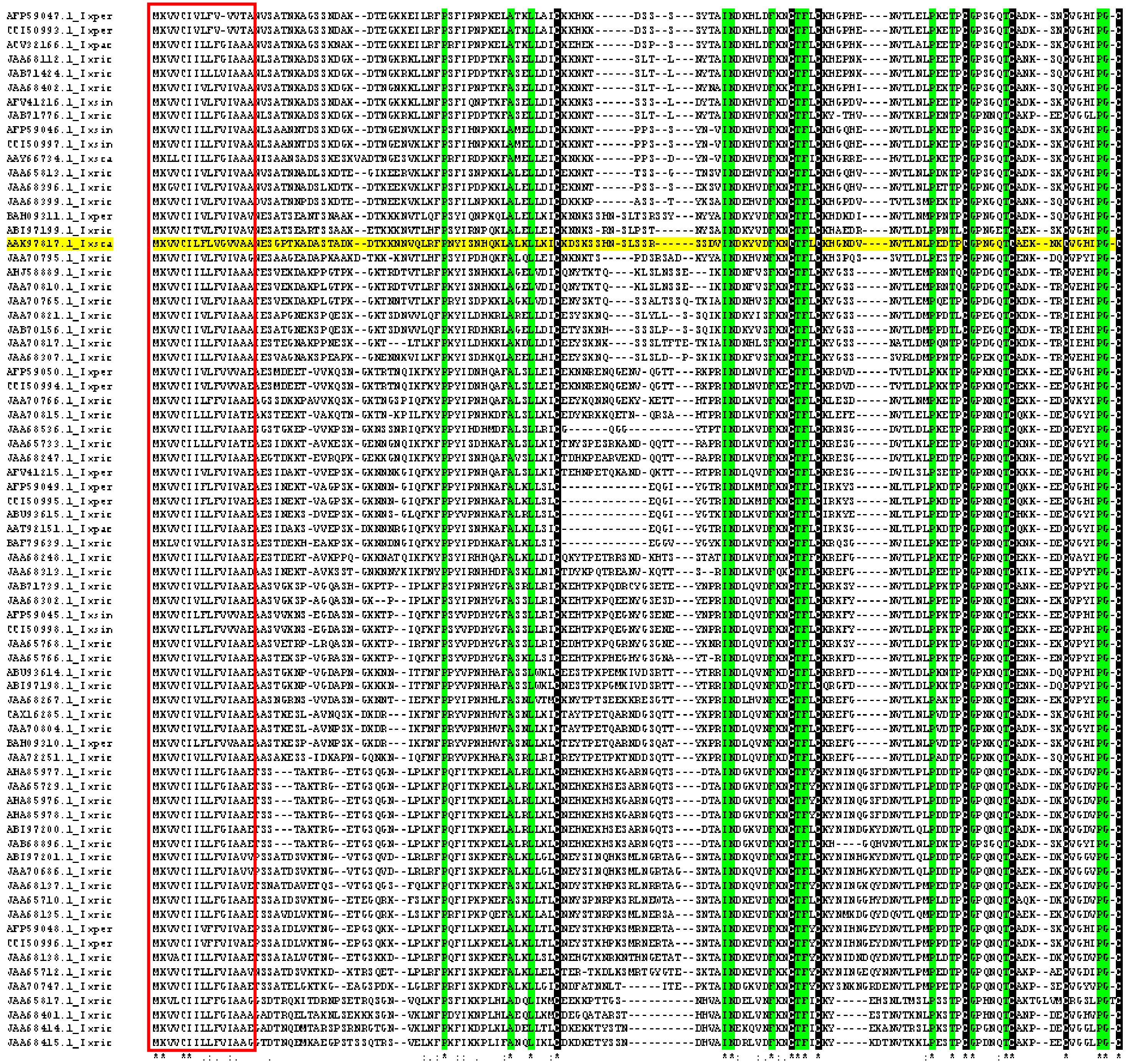

Figure S13: Clustal alignment of members of the Salp15 family of proteins. The seven conserved cysteines are shown in black background. The remaining identical sites are shown in green background. The sequence coding for Salp15 (AAK97817.1) is shown in yellow background.

The proposed disulfide bridges are:

|C:1 C:3|C:2 C:5|C:4 C:6|

The resulting prosite block is:

P-x(9)-A-x(2)-L-x(3)-C-x(8,24)-I-N-x(5)-F-x(2)-C-T-F-x-C-x(11,16)-P-x(2)-T-x-C-G-x(4)-T-C-x(5,7)-C-x(4)-P-G-x(0,1)-C.

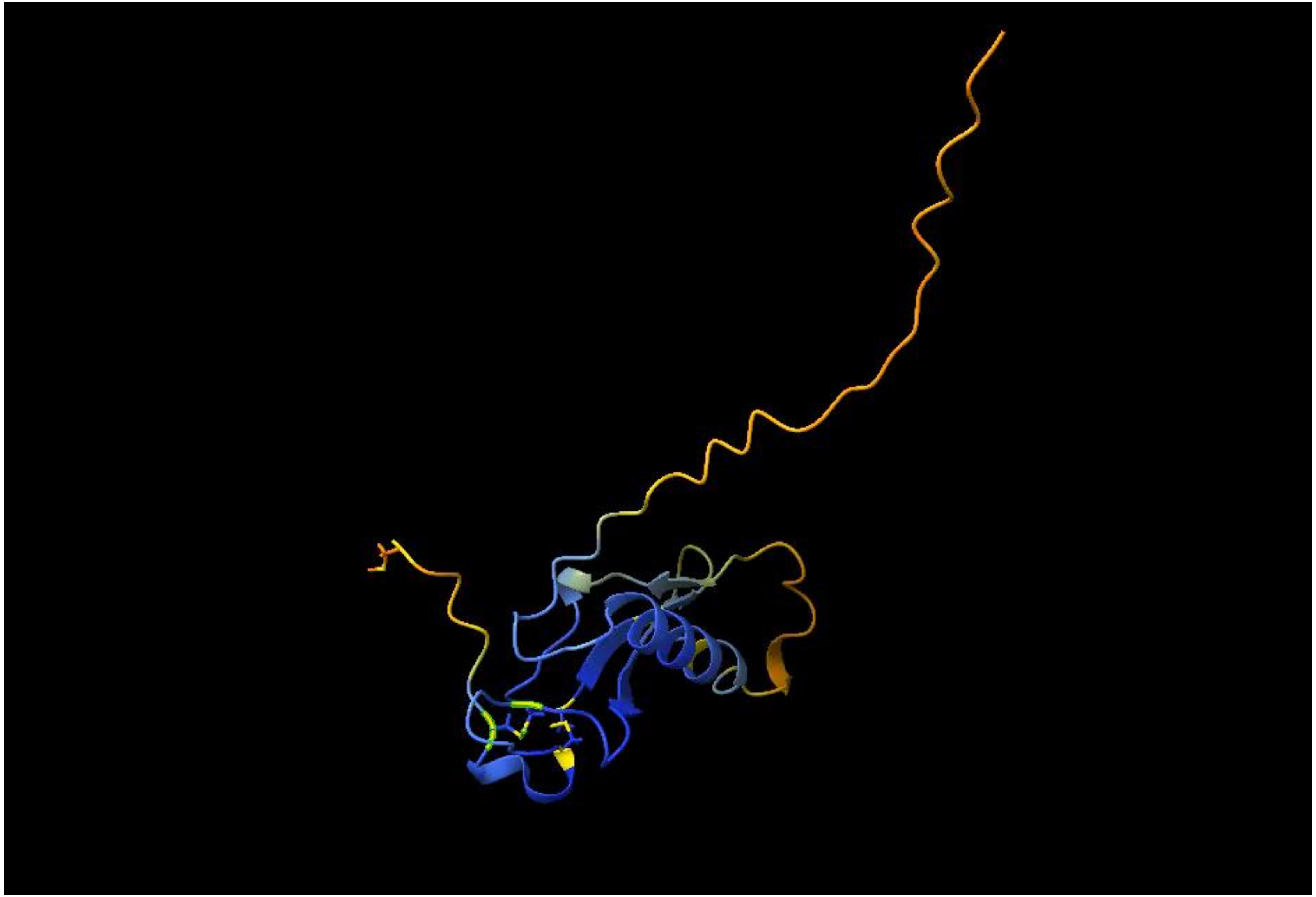

Figure S14: Predicted alphafold structure of Salp15 showing disulfide bonds. The structure is colored according to the B factors, where blue represents higher confidence and red lower confidence. Cysteine residues are colored in yellow.

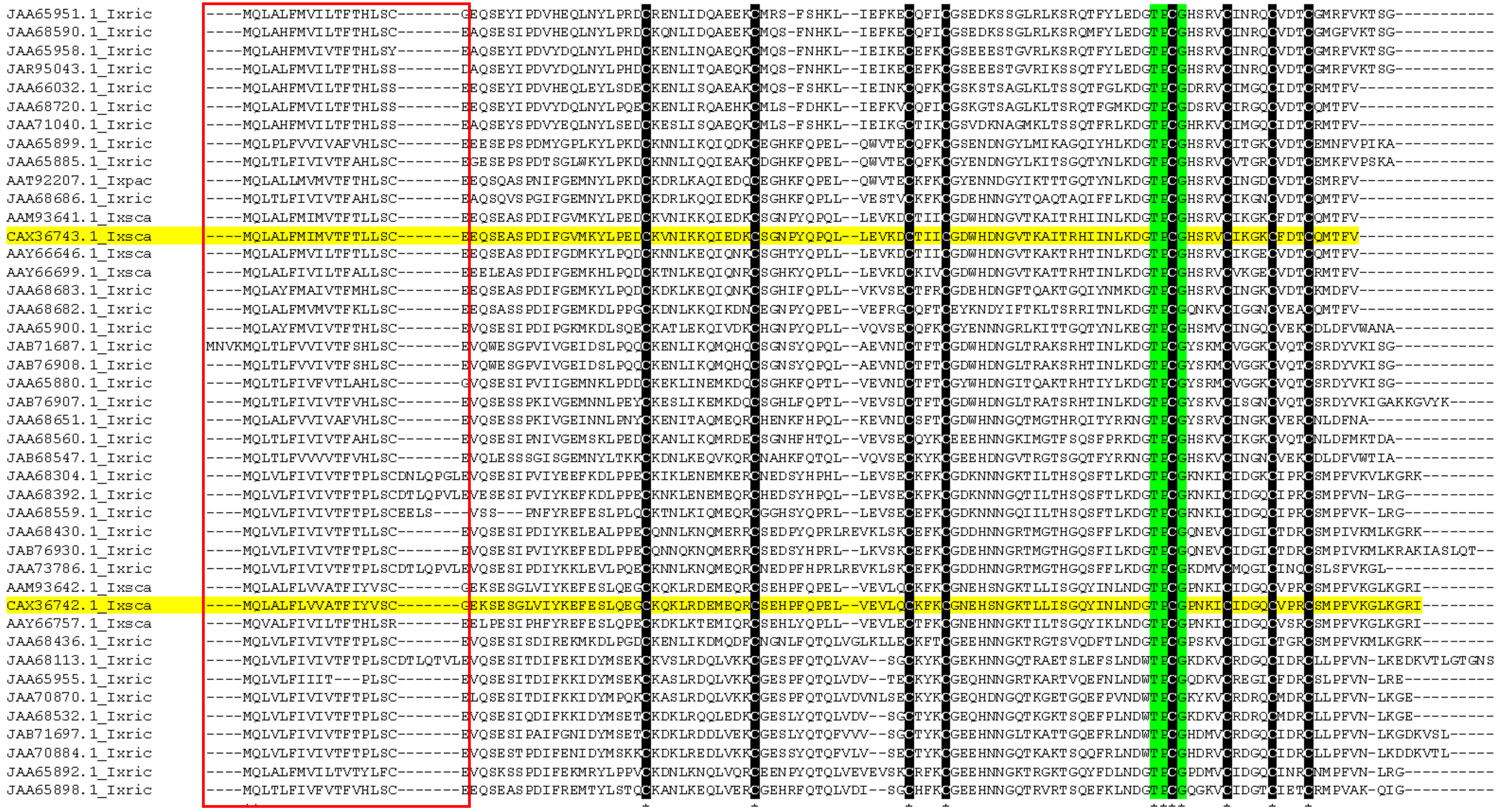

Figure S15: Clustal alignment of members of the Ixostatin family of proteins. The eight conserved cysteines are shown in black background. The remaining identical sites are shown in green background. The red box delineates the signal peptide region. The sequences coding for ISL929 (CAX36743.1) and ISL1373 (CAX36742.1) are shown in yellow background.

The proposed disulfide bridges are: |C:1 C:3|C:2 C:5|C:4 C:6| The resulting prosite block is: PA C-x(11)-C-x(13,16)-C-x(3)-C-x(22)-T-P-C-G-x(4)-C-x(4)-C-x(3)-C.

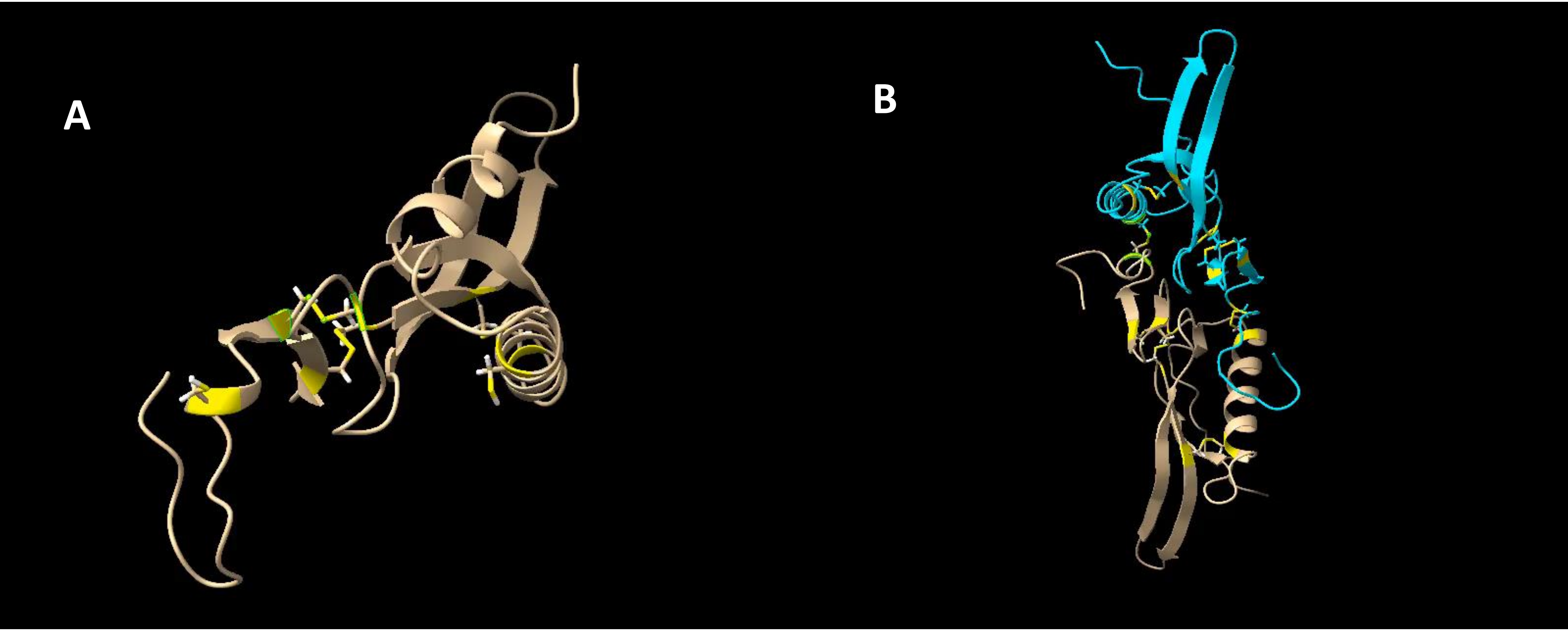

Figure S16: Predicted Alphafold structure for the Ixostatin CAX36742.1. A) Monomer with disulfide bonds |A:2 A:4 |A:3 A:6 |A:5 A:7|
B) Dimer with disulfide bonds |A:2 A:4 |A:3 A:6 |A:5 A:7 |B:2 B:4 |B:3 B:6 |B:5 B:7 |A:1 B:8 |A:8 B:1

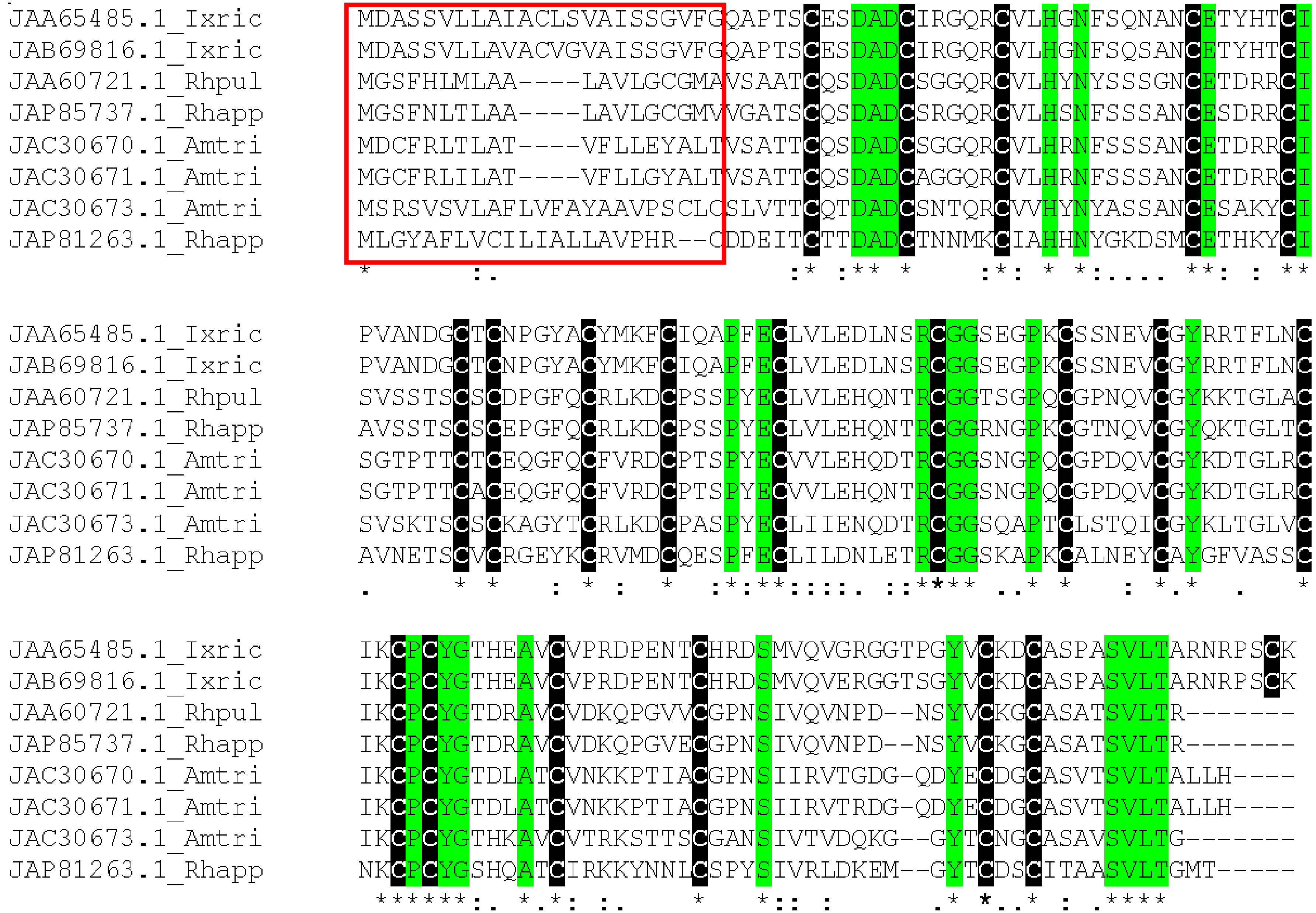

Figure S17: Alignment of the 20cys subgroup of the Salp15/Ixostatin group of tick salivary proteins. The conserved cysteines are shown in black background. Other conserved amino acids are shown in green background. The red box delineates the signal peptide region.

A

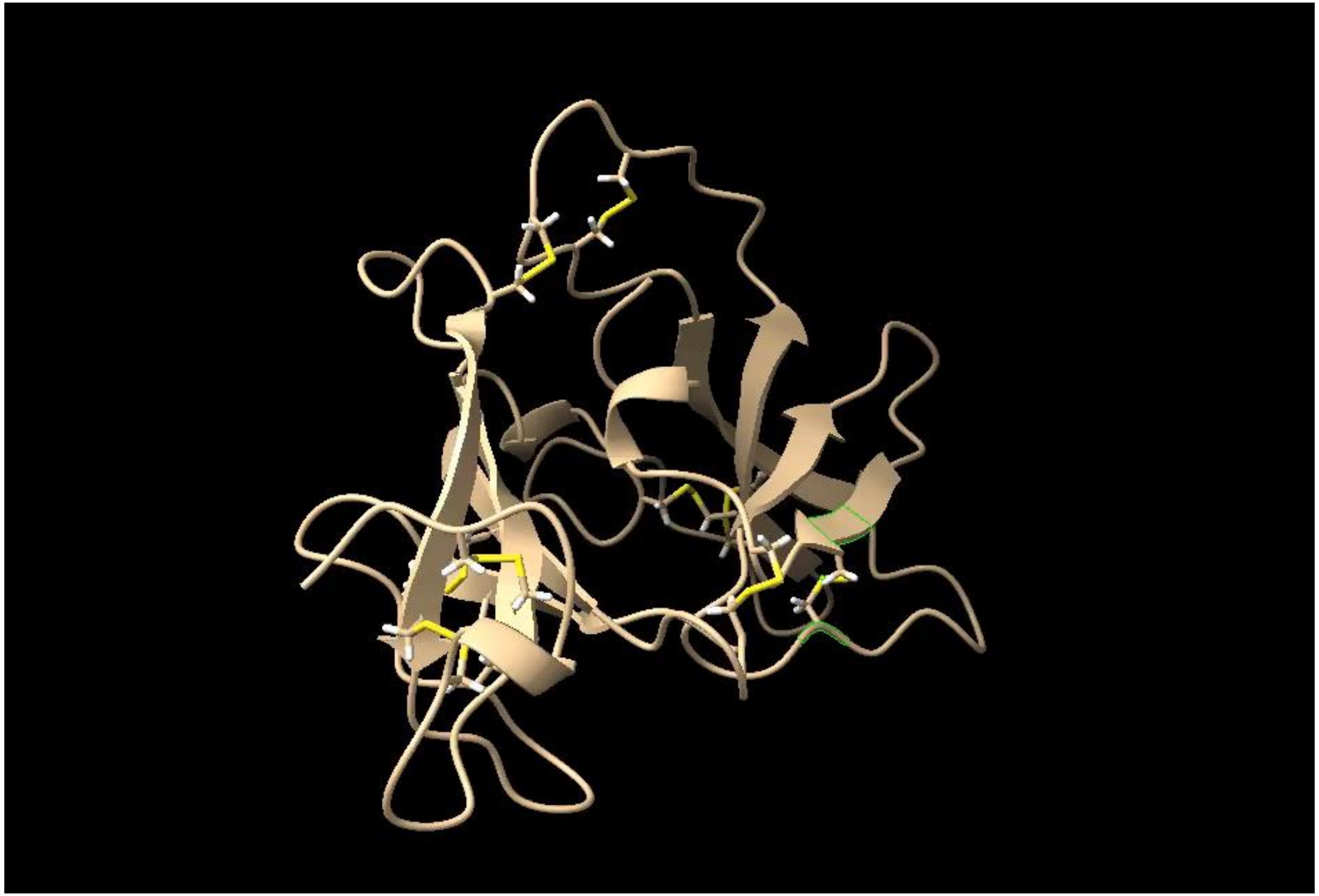

B |A:1 A:3|A:2 A:5|A:4 A:15|A:6 A:8|A:7 A:10|A:9 A:20|A:11 A:13|A:12 A:17|A:14 A:16|A:18 A:19|

Figure S18: A) Predicted Alphafold structure of JAA60721.1, a 20cys subgroup member of the Salp15/Ixostatin group of tick salivary proteins. The 20 cysteines form disulfide bonds as indicated in B.

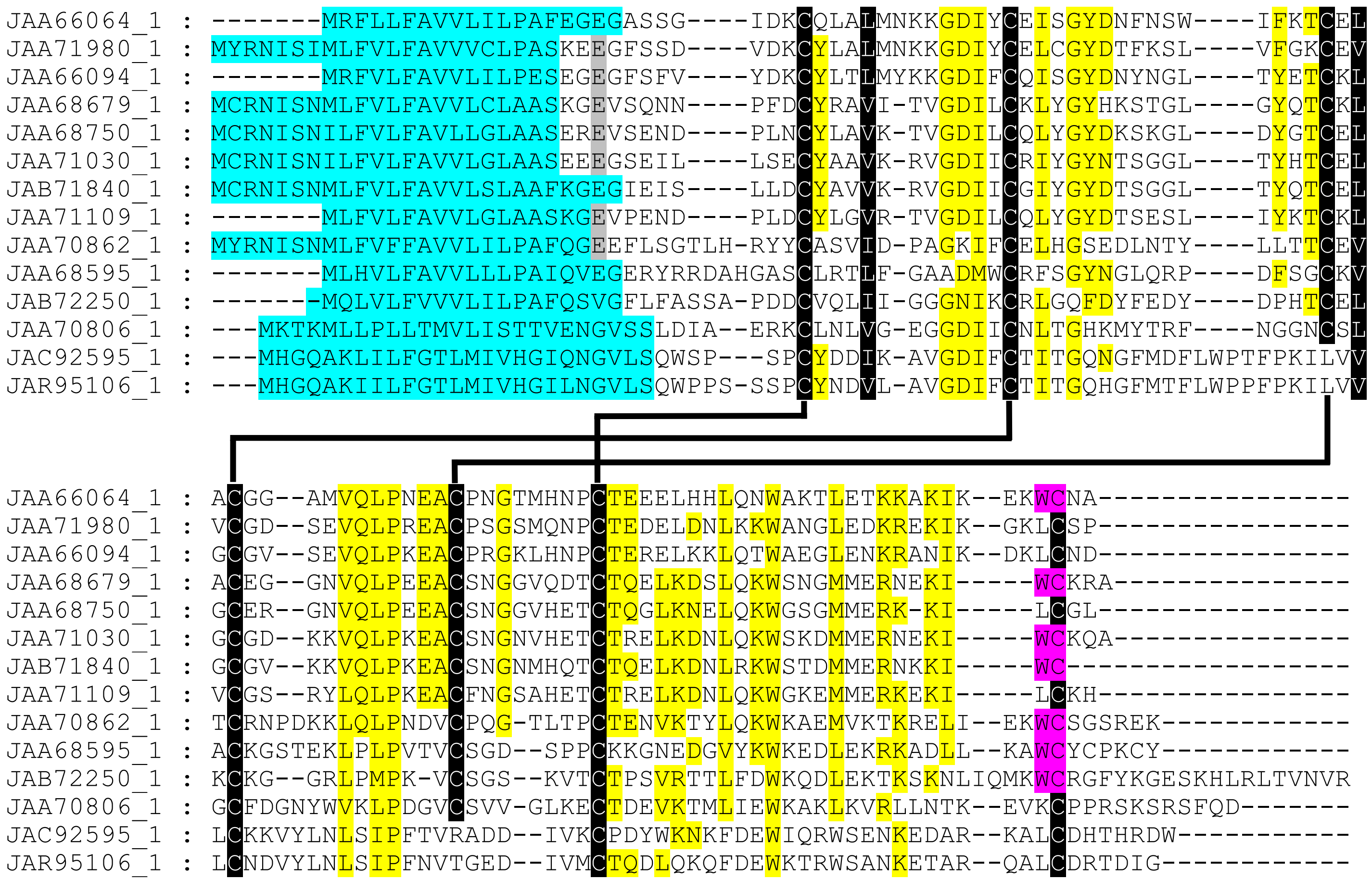

Figure S19: Alignment of selected members of the 10kDa-WC family. Indicated are the conserved cysteines and their disulfide bond pattern. Conserved residues are highlighted in yellow, while the WC motif is highlighted in purple. The predicted signal peptides are highlighted in light blue.

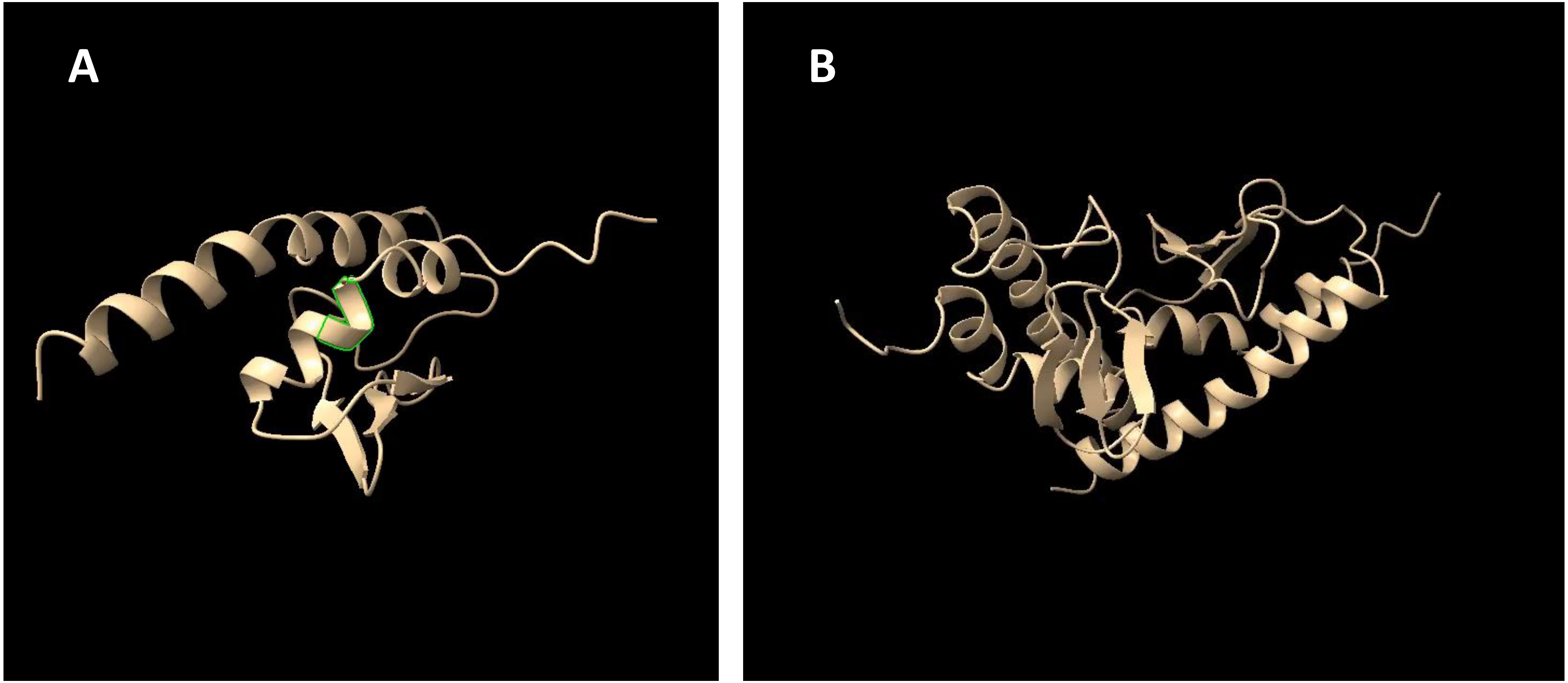

Figure S20: Predicted structure of the 10kDa-WC family. A) Monomer B) Dimer.

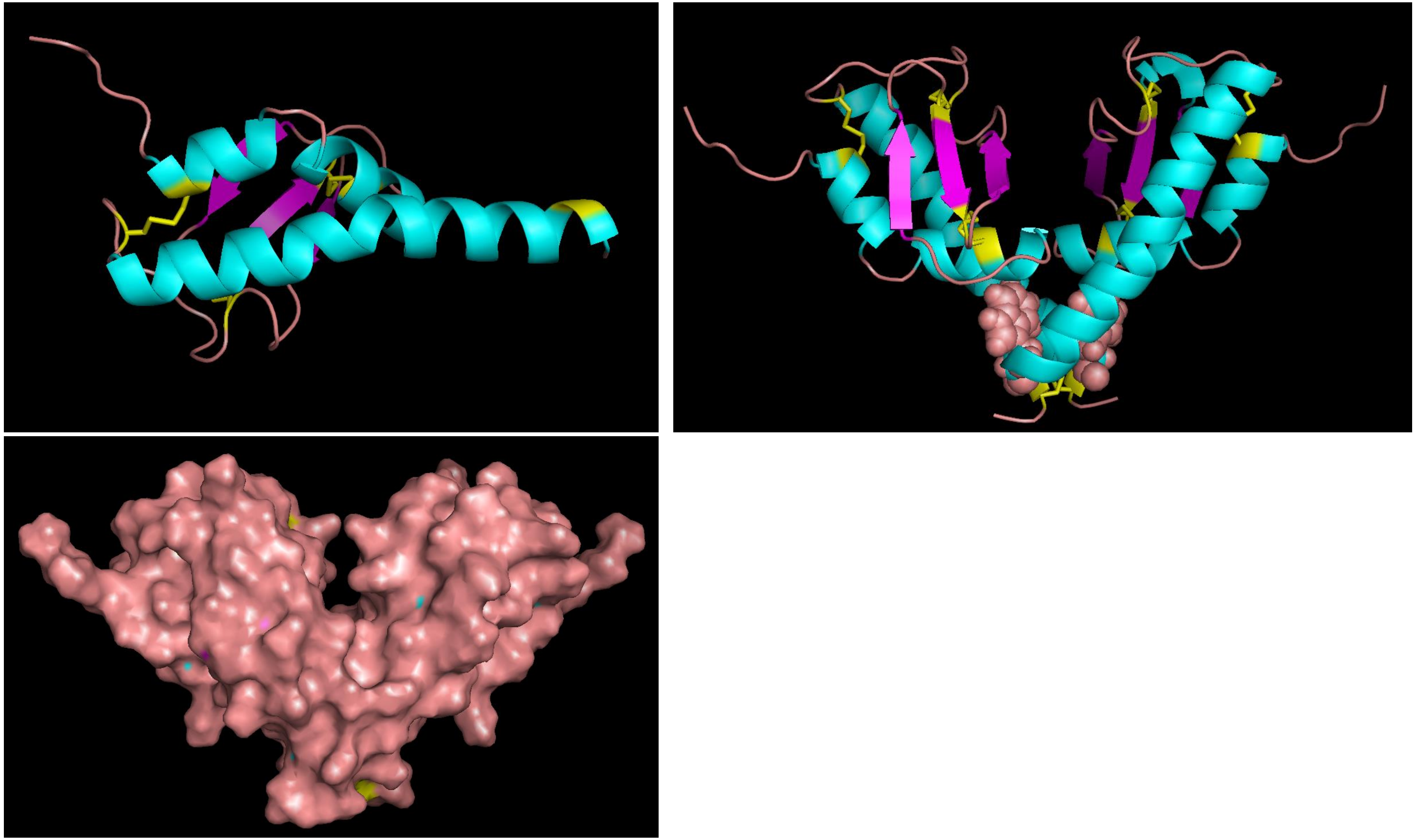

Figure S21: Predicted structure of the 10kDa-WC family. Top left are the N-terminal alpha-helices (top), the central anti-parallel beta-sheet (purple) and the C-terminal alpha-helix. disulfide bonds and cysteines are highlighted in yellow. Top right indicate the predicted homodimer with the formation of a disulfide bond by the C7 cysteines of each monomer at the bottom of the structure and the potential binding cleft at the top. Also indicated are the tryptophanes of the WC motif. The bottom right indicate a surface model of the dimer that indicates the binding cleft formed.

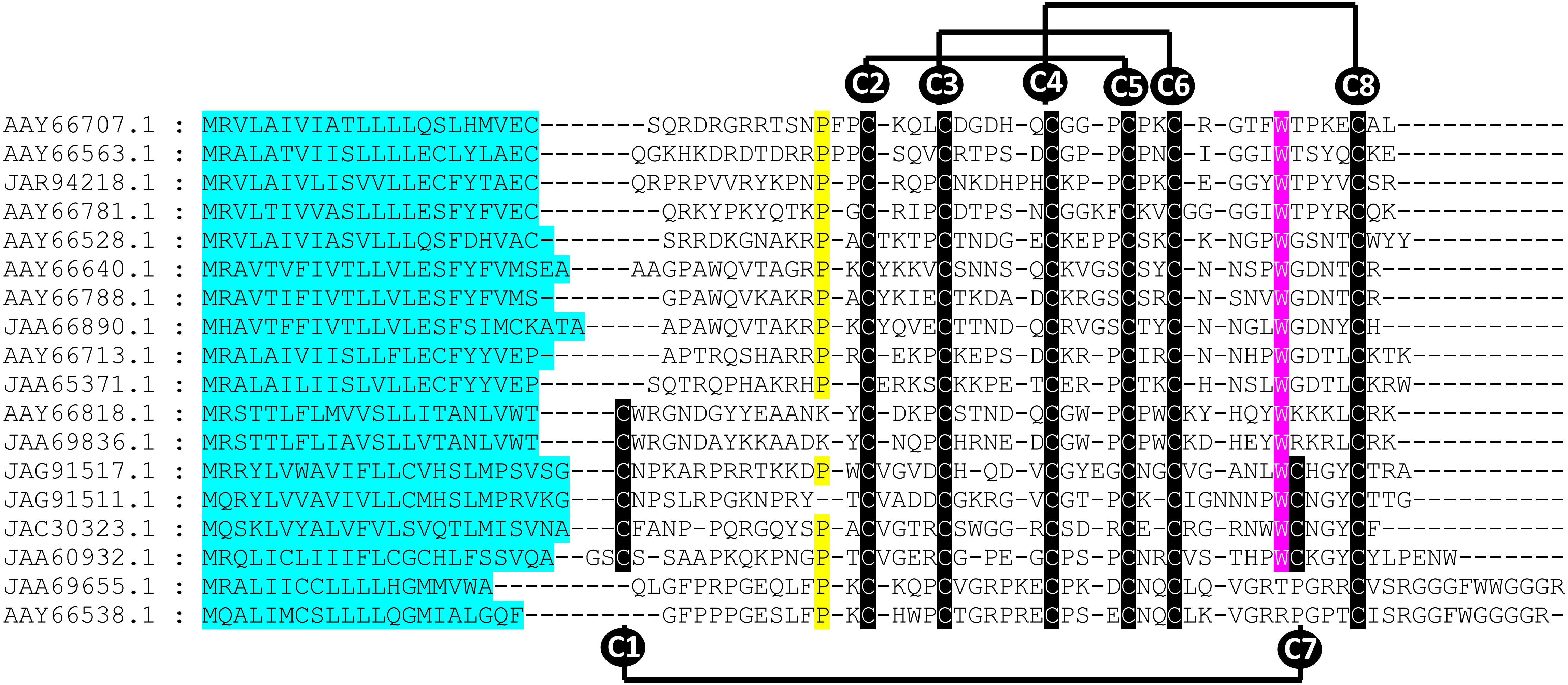

Figure S22: Alignment of selected members of the 5.3kDa family. Indicated are the conserved cysteines and their disulfide bond pattern. Conserved residues are highlighted in yellow, while a conserved W is highlighted in purple. The predicted signal peptides are highlighted in light blue.

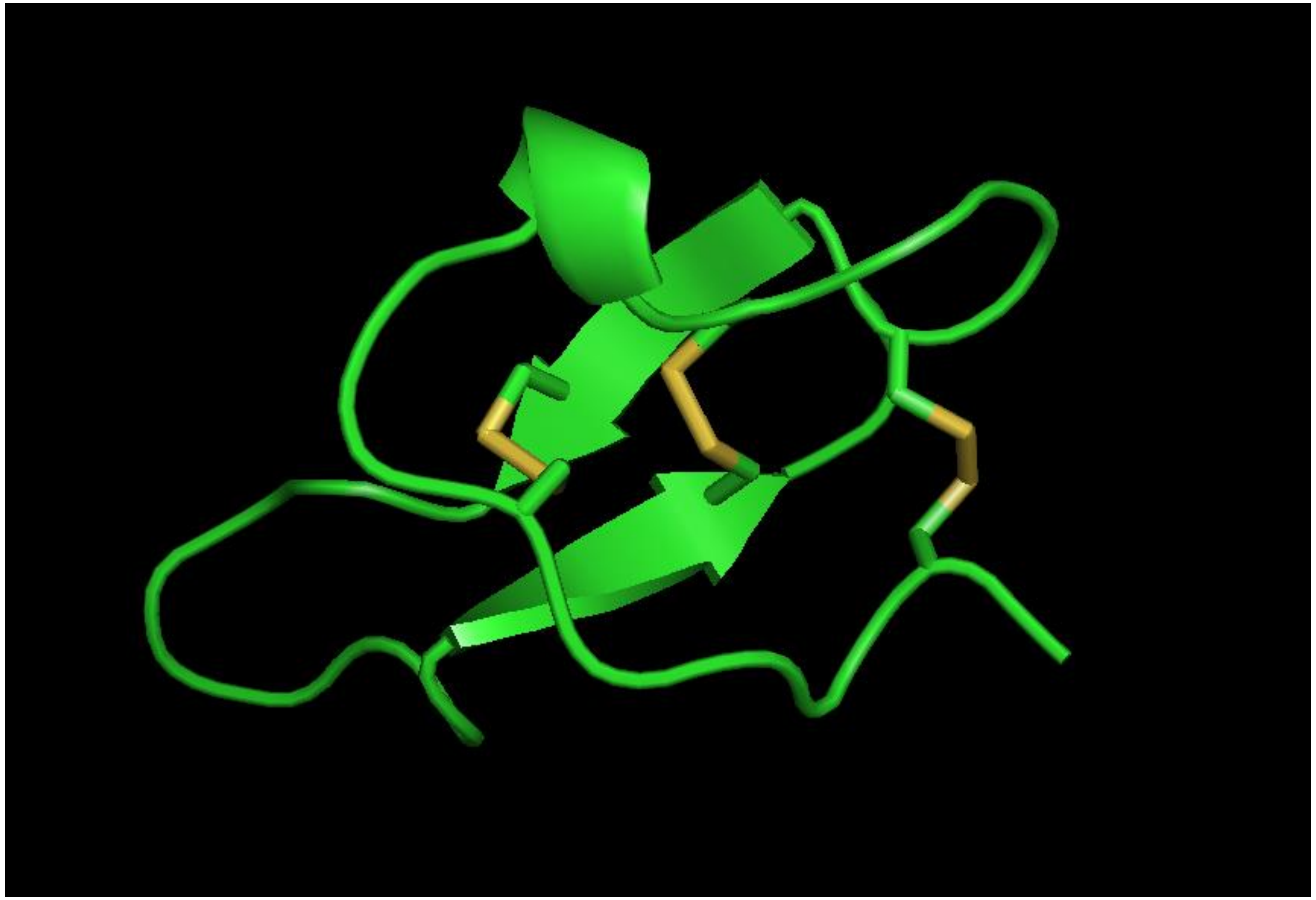

Figure S23: Predicted structure of the 5.3kDa family. Indicated are the central fold composed of two anti-parallel beta-sheets linked with three disulfide bonds that forms a cysteine knot.

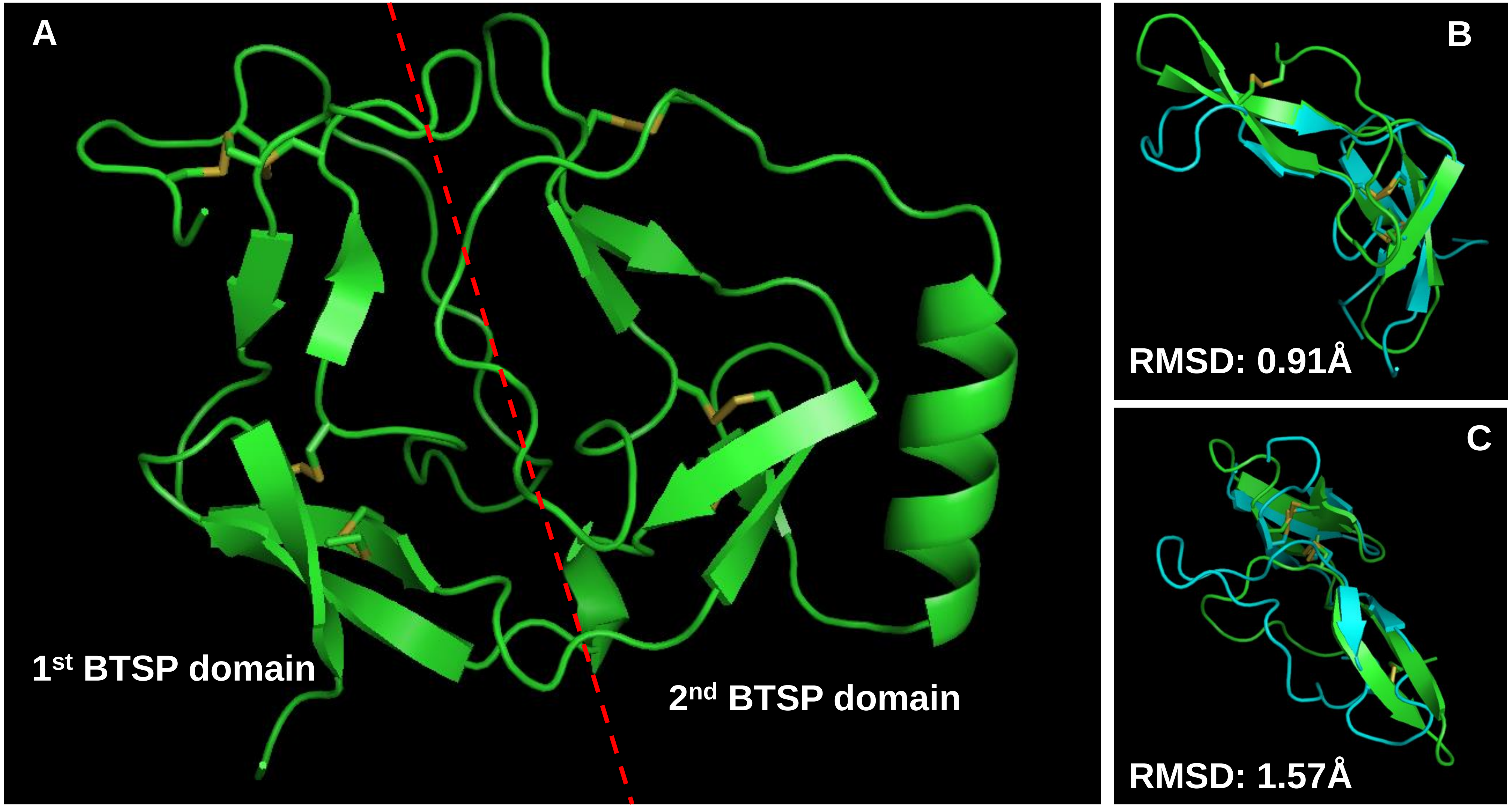

Figure S24: Predicted structure of the 7 disulfide bond family. A) The N-terminal start top left and the C-terminal end bottom left. The two BTSP-like domains are separated by the dashed red line. B) Superposition of the BTSP BSAP1 and the first 7DB-domain. C) Superposition of the BTSP BSAP1 and the second 7DB-domain.

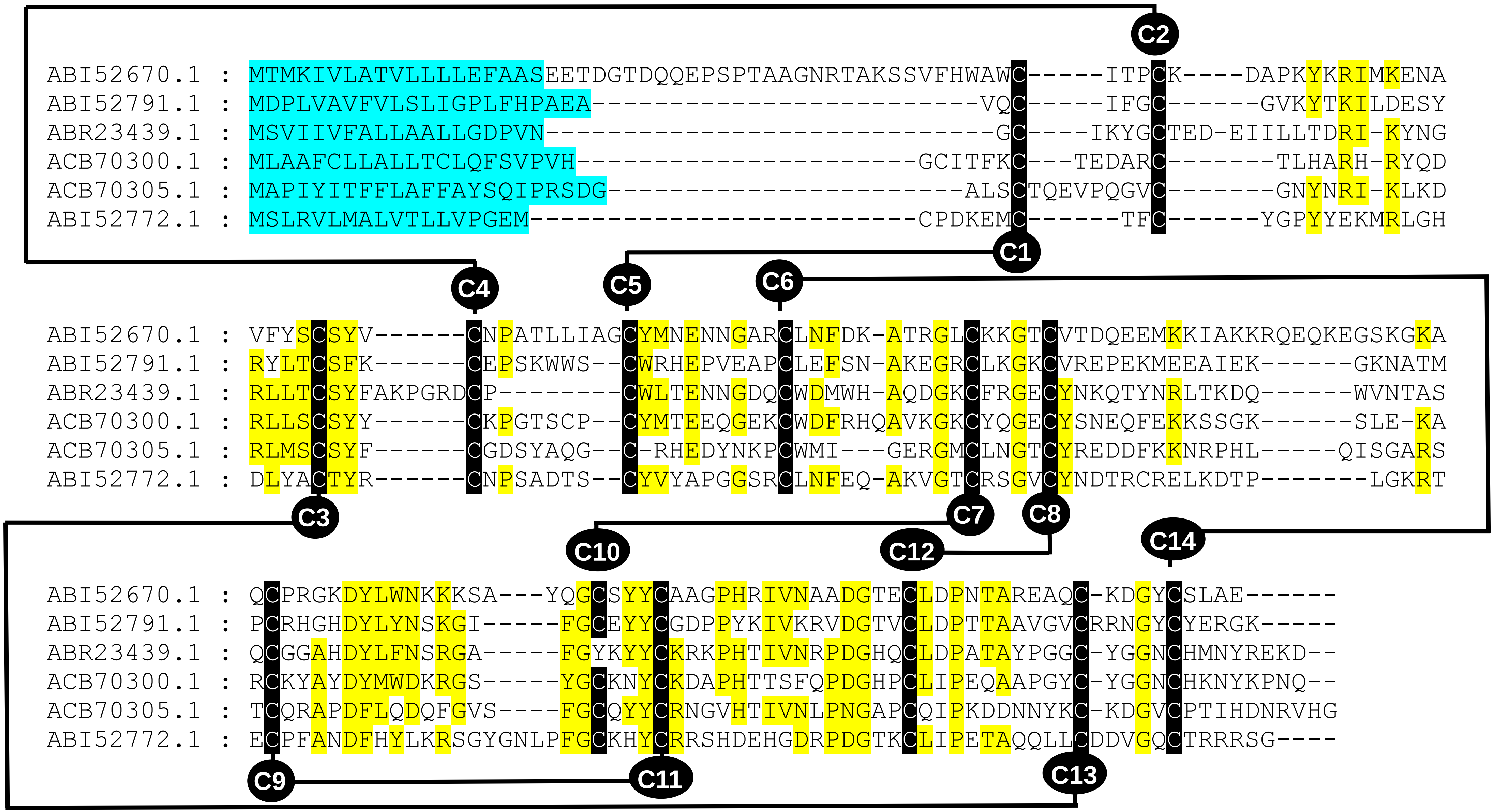

Figure S25: Alignment of selected members of the 7 disulfide bond family. Indicated are the conserved cysteines and their disulfide bond pattern. Conserved residues are highlighted in yellow. Signal peptides are highlighted in blue.

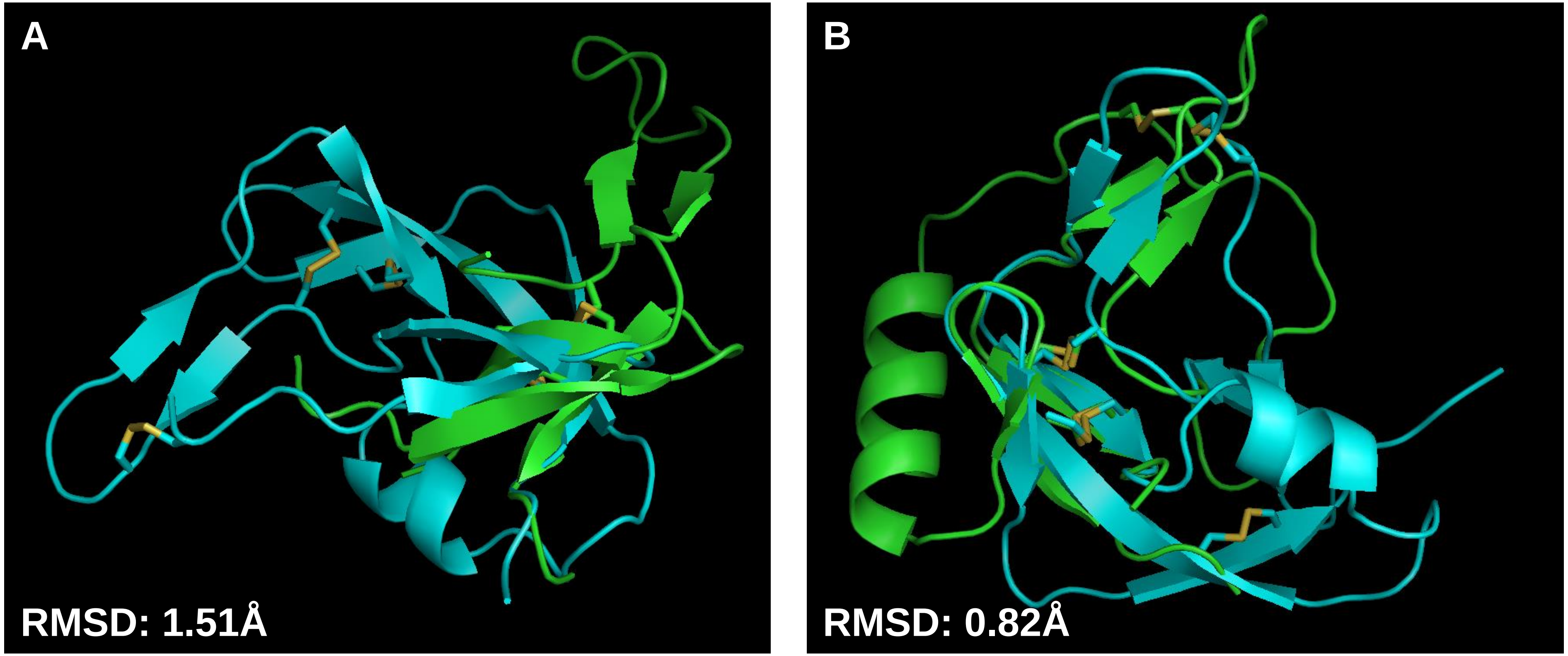

Figure S26: A) Superposition of the “first” 7DB-domain and the Evasin-1 fold. B) Superposition of the “second” 7DB-domain and the Evasin-1 fold.

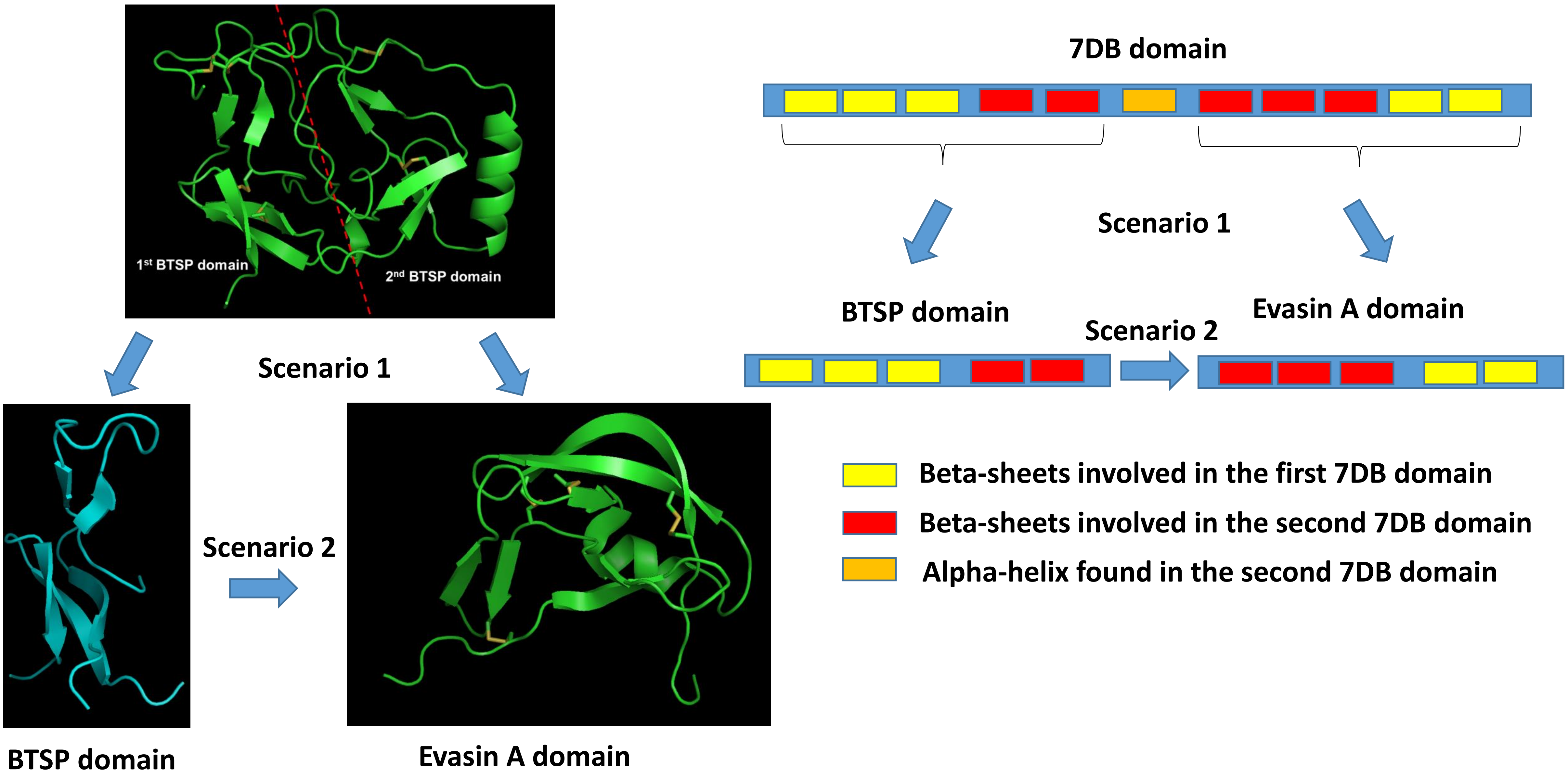

Figure S27: Model for the evolution of the 7DB, BTSP and Evasin A families. Indicated are the first and second domains of the 7DB family and how duplication of respective beta-sheet configurations may have resulted in the different BTSP and Evasin A domains.

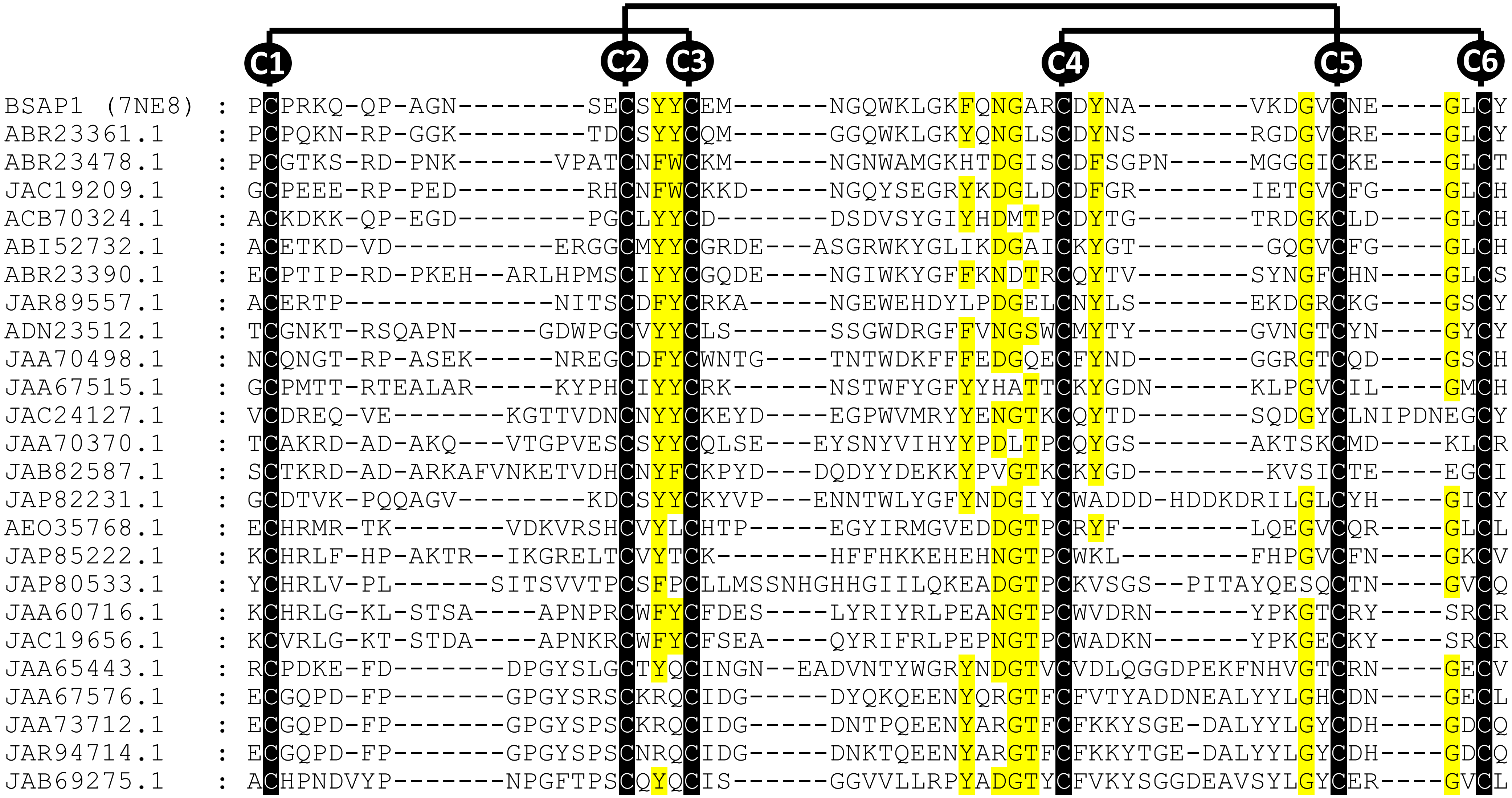

Figure S28: Alignment of selected members of the BTSP family. The alignment has been trimmed to the core domain. Indicated are the conserved cysteines and their disulphide bond pattern. Conserved residues are highlighted in yellow.

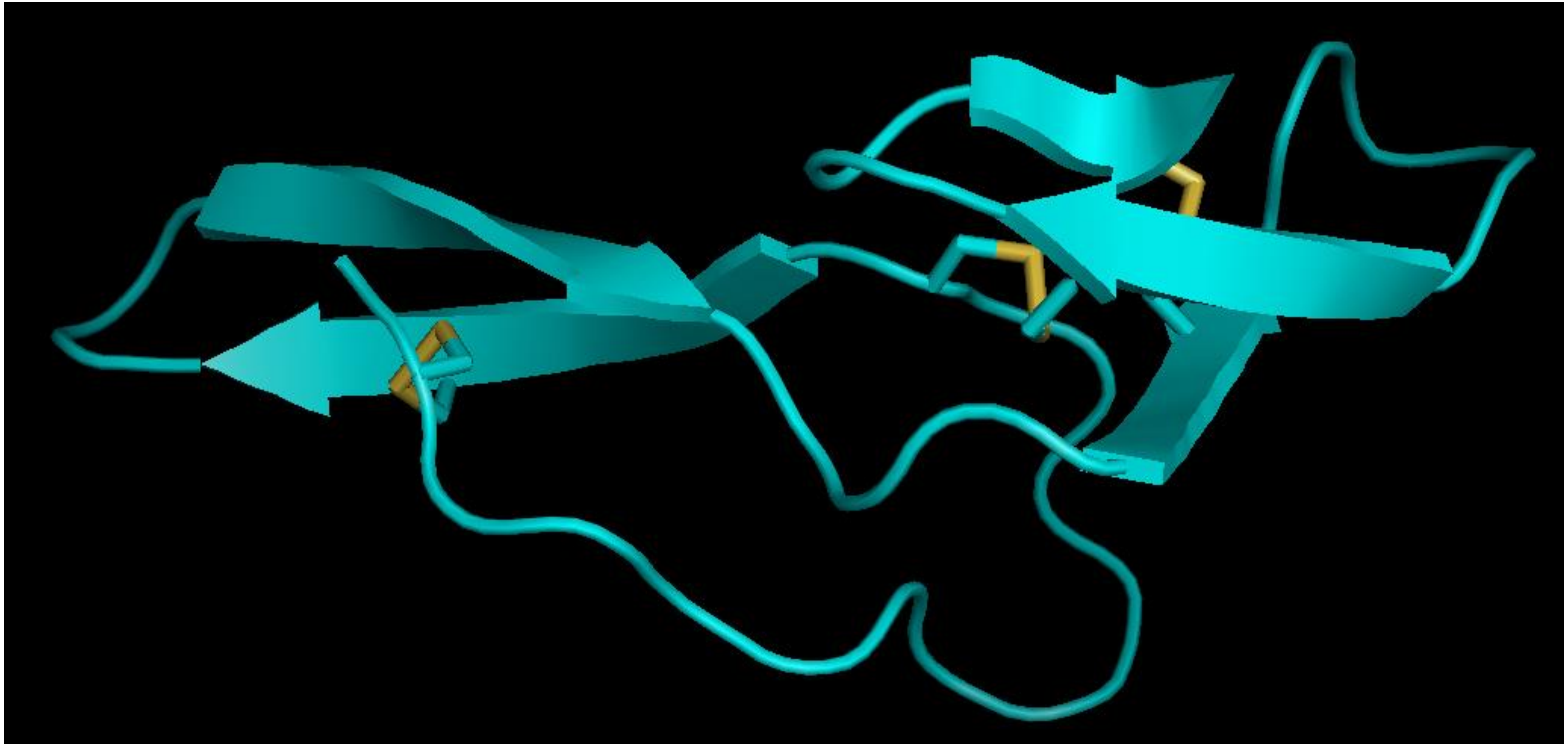

Figure S29: Predicted structure of the BTSP family. Indicated are the central fold composed of two domains, an N-terminal domain (left) composed of two anti-parallel beta-sheets and a C-terminal domain (right) composed of three anti-parallel beta-sheets. Disulphide bonds stabilize the fold by linking the N-terminal to the first domain, while remaining disulphide bonds stabilize the C-terminal domain.

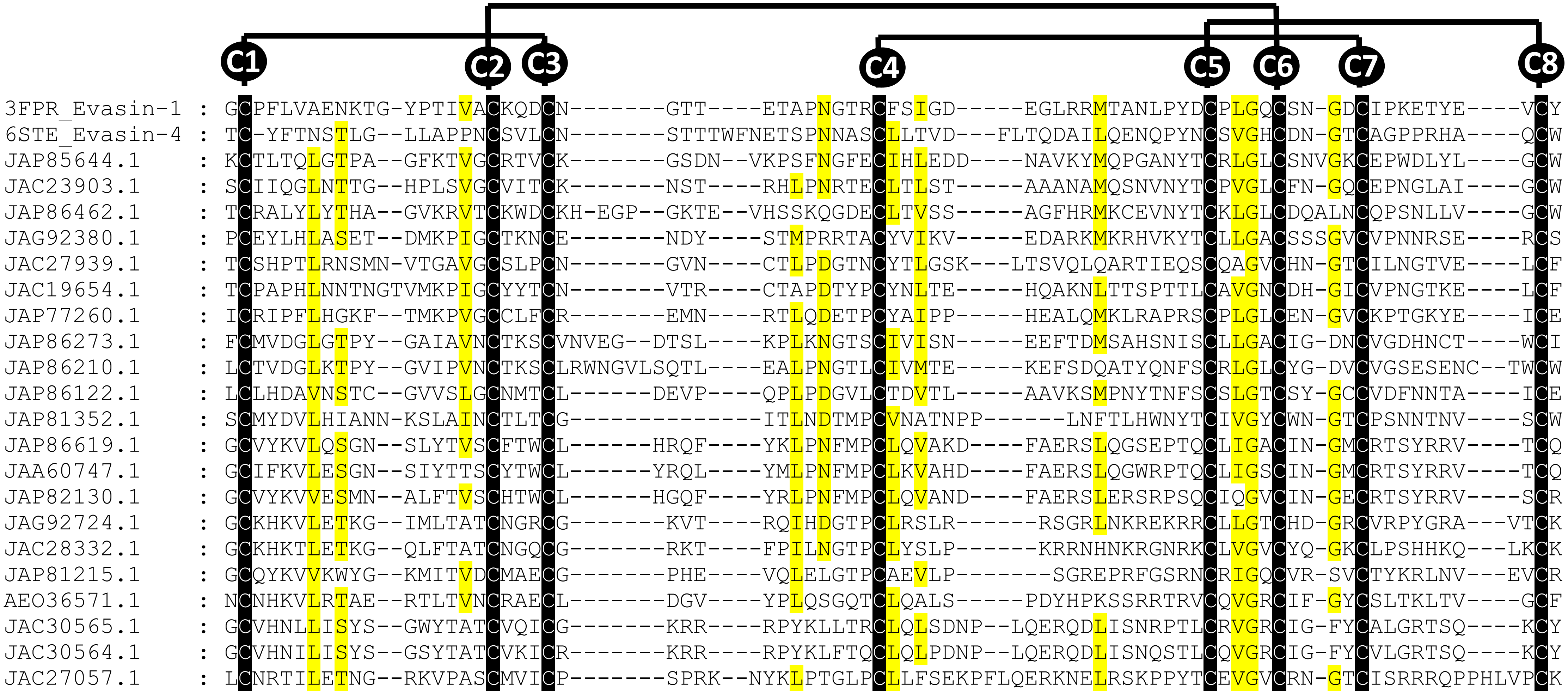

Figure S30: Alignment of selected members of the Evasin A family. Evasin-1 and Evasin-4 is included. Indicated are the conserved cysteines and their disulfide bond pattern. Conserved residues are highlighted in yellow. The alignment was trimmed to represent the core domain. The structure of Evasin-3 with disulfide bonds is also indicated below the alignment.

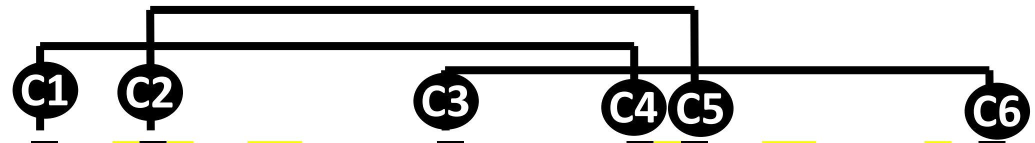


```
6I31       : LVSTIESRTSGDGADNFDVVSCNKNCTSGQN----ECPEG---CFCGLLGQNKKGHCYKII
JAA69499.1 : SADSKGTSDSQDSTKSIKVDFCETNCTK-TDGGWTGCTGD---CICVSVGDSIEGRCMDFG
JAA69478.1 : SAESNEQGSSPSSGKSLKVDFCETNCTE-QDGVWSDCTGD---CFCVHVGDSKESRCMHVI
JAA69096.1 : SAGSKERSAPQSSENSIKAEFCDTNCTEGPDKTWSDCSPG---CFCVHVGNSTEGRCMTFN
JAA69342.1 : VALTDAAGNDD---ELFSVEYCGMNCTKKEDGTWTGCTGKKGGCKCYHENGKGDGLCLSTV
```

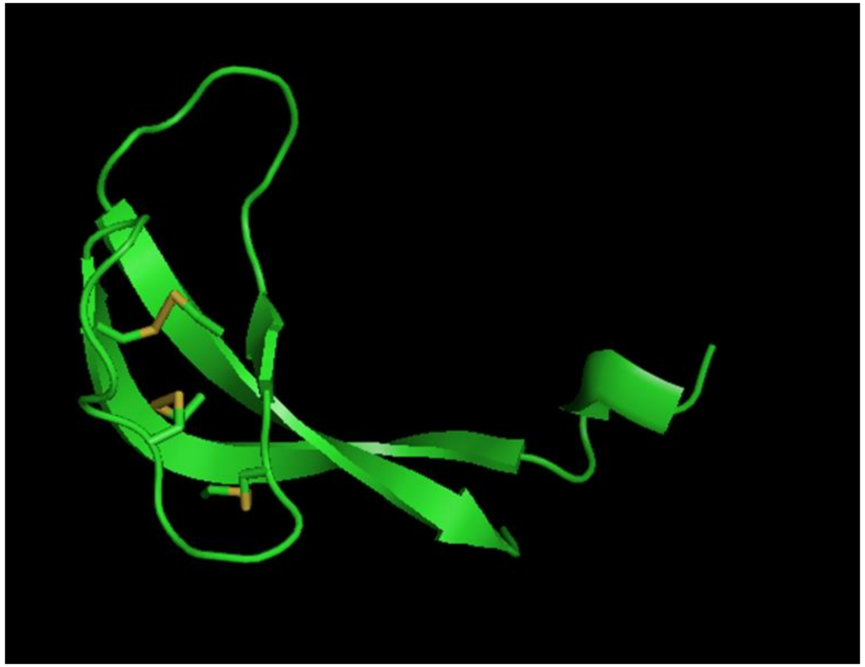

Figure S31: Alignment of selected members of the Evasin B family. Evasin-3 is included (6I3L). Indicated are the conserved cysteines and their disulfide bond pattern. Conserved residues are highlighted in yellow. The alignment was trimmed to represent the core domain. The structure of Evasin-3 with disulfide bonds is also indicated below the alignment.

```
AEO35915.1 : MKTAVVFVLTACLLVGV-HAGTRTDTRRAEFDRWRQCMVDKLPTDKAPVFDDCQSRASGTEMRKFREGLQCVL
JAC30533.1 : MKTAVVFVLTACLLVGV-HAGTRTDSRRTEYDQWRQCMVDKLPTDKAPVFDECQTRASGTEMRKFREGLQCVL
JAC30534.1 : MKTAVVFVLTACLMVGV-HAGTRTDSRRAEYDQWRQCMVDKLPTDKAPVFDECQTRASGTEMRKFREGLQCVL
JAC30535.1 : MKTAVVFVLTACLLVGV-HAGTRTDSRRAEYDQWRRCMVDKLPSDKAPVFDECQTRASGTEMRKFREGLQCVL
JAC30536.1 : MKTAVVFVLTACLLVGV-HAGTRTDSRRAEYDQWRQCMVDKLPTDKAPVFDECQTRASGTEMRKFREGLQCVL
JAC23862.1 : MKTAVVFLLTACLLARA-SAGSRAETRRAQFDQWRQCMVDKLPTDKEPVFRGCHDTASGTDMRKFRQGLECVL
JAC26572.1 : MKTAVVFLLTACVLARA-SAGSRAESRRAQFDQWRQCMVDKLPTDKAPVFQGCHNTAAGTEMRKFRQGLECIL
JAG92140.1 : MKTAVVFLLTACLLARA-SAGSRAESRRAQFDQWRQCMVDKLPTDKAPVFQGCHNTAAGTEMRKFRQGLECVL
JAP65894.1 : MKTAVVLVLVACFVATATSAKGRAASRREQFDEWRNCMVQKIPNDKASVYQACHERSRGTDMHRFRDGLQCVL
JAP77831.1 : MKTAAVLLLVACFVAVATSAKDRTTSRRQQFDNWRSCMVKKIPGDKAPVYEACHNRSRGTDMHRFRDGLQCVL
JAP86044.1 : MKTAVVLVLAACLVAVAMSAGGRISPSREDFAEWRACMVAKLPQDKVQEYENCRNRSRGTDMRRFRQGLECVL
JAA65678.1 : MKILLITVVV-CVFAA--TDASRRSTRRQQFTDWRNCMVEQLPSEKKPIYDACYNRSRGTDMHKFREGLKCVL
JAA65679.1 : MKILLITIVV-CVFAA--TDASRRSTRRQQFDDWRACMVGQLPTEKKPIYEACHNRSRGTDMHKFREGLKCVL

AEO35915.1 : GSYQLVNGNNVNLAQMTQVAPTIQKEDLKKAFEECPKDDGNRRVAKAVKCVIDHLENTCPVPSGAQN---
JAC30533.1 : GSYQLVNGNNVNLAQMTQVAPTIQKQDLKKAFEECPKDDGNTRIAKAVKCVIDHLKNTCPVPSGAQN---
JAC30534.1 : GSYQLVNGNNVNLAQMTQVAPTIQKQDLKKAFEECPKDDGNTRIAKAVKCVIDHLKNTCPVPSGAQN---
JAC30535.1 : GSYQLVNGNNVNLAQMTQVAPTIQKEDLKKAFEECPKDDGNRRVAKAVKCVIDHLENTCPVPSGAQN---
JAC30536.1 : GSYQLVNGNNVNLAQMTQVAPTIQKEDLKKAFEECPKDDGNRRIAKAVKCVIDHLENTCPVPSGAQN---
JAC23862.1 : GSYQLVDRNNVNLARMTEVAPTIAKEELKKAFEDCPKDDGNKKVAKAVKCVIDHLETTCPVPAGAQS---
JAC26572.1 : DSYQLLDRNNVNLARMTEVAPTITKEELKKAFEDCPKDDGNKKVAKAVKCVIDHLETTCPVPSGAQS---
JAG92140.1 : DSYQLVDRNNVNLARMTEVAPTIAKEELKKAFEDCPKDEGNKRVAKAVKCVIDHLETTCPVPSGALS---
JAP65894.1 : SSYNIVNRNDVNLERMAQLAREITQQDLKSAFEECPKNERNKQLQRTVKCVIDHLESTCPVPEGAAGARE
JAP77831.1 : SSYNIVKKNDVNLDLMAQLAKNITQQDLKGAFEECPKNDRNKNLQRAVKCVIDHLESTCPAPDGAAGSRK
JAP86044.1 : SSYNLVNRTDVDLSRMQQLAQNVTQEEVRAAFHDCSQDENNKKVGKAVKCFIDHLETSCPVPADANKE--
JAA65678.1 : TDYNLLNGTKIDLAAMTSRASSVSGK-LGQAFTDCPKDDHNRNAAKGVKCIIDRLETDCPVPTGSTTR--
JAA65679.1 : TDYSLLNGTKIDLAAMTTRASSVSGK-LGQAFTDCPKDDHNRNAAKGVKCIIDRLETDCPVPTGSTTR--
```

Figure S32. Alignment of the 13kDa-basic family. Conserved residues are highlighted in yellow and the signal peptides in light blue. Conserved cysteines with their predicted disulphide pattern is indicated.

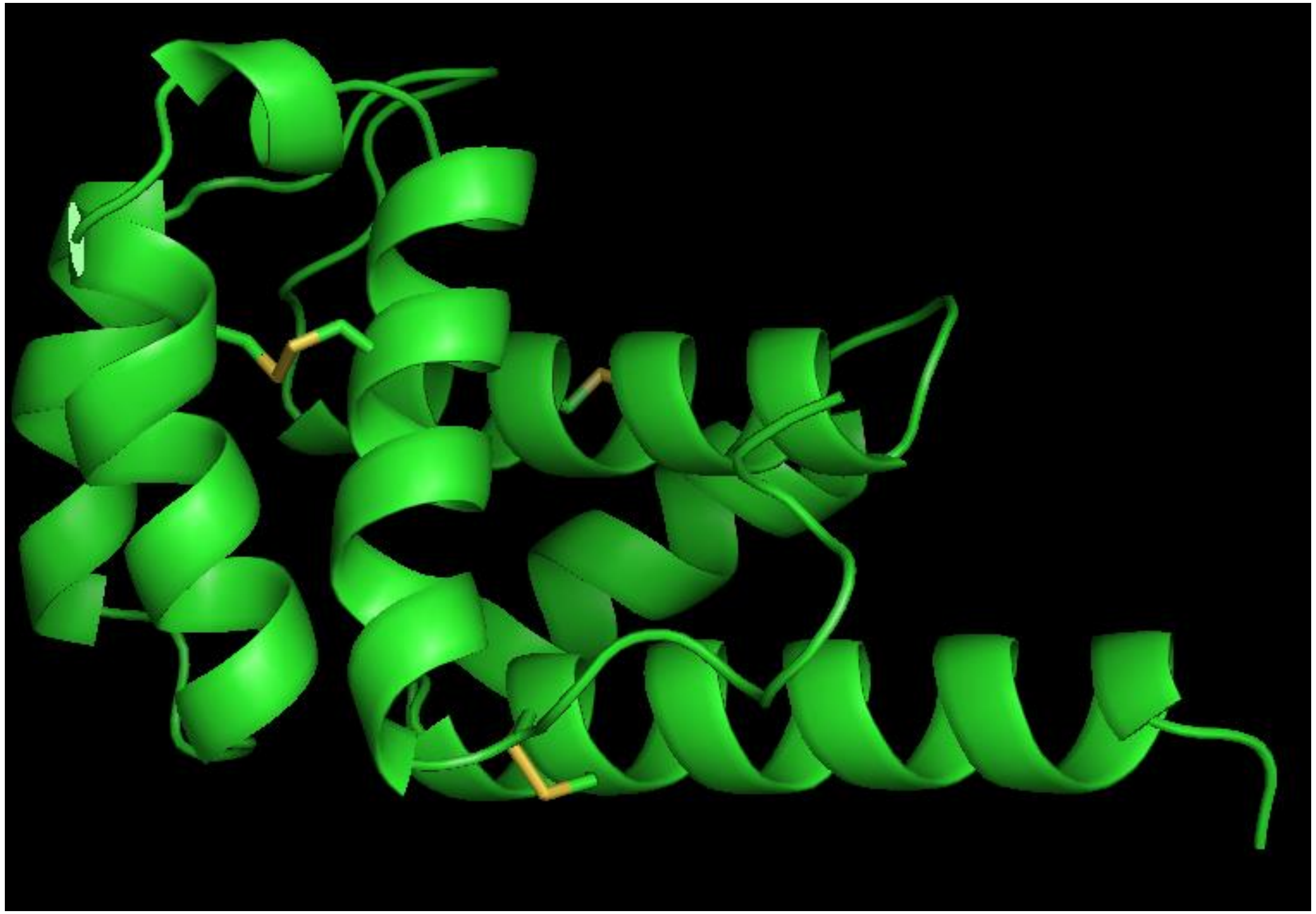

Figure S33. Predicted structure of the 13kDa-basic family. Disulfide bonds are indicated in yellow.

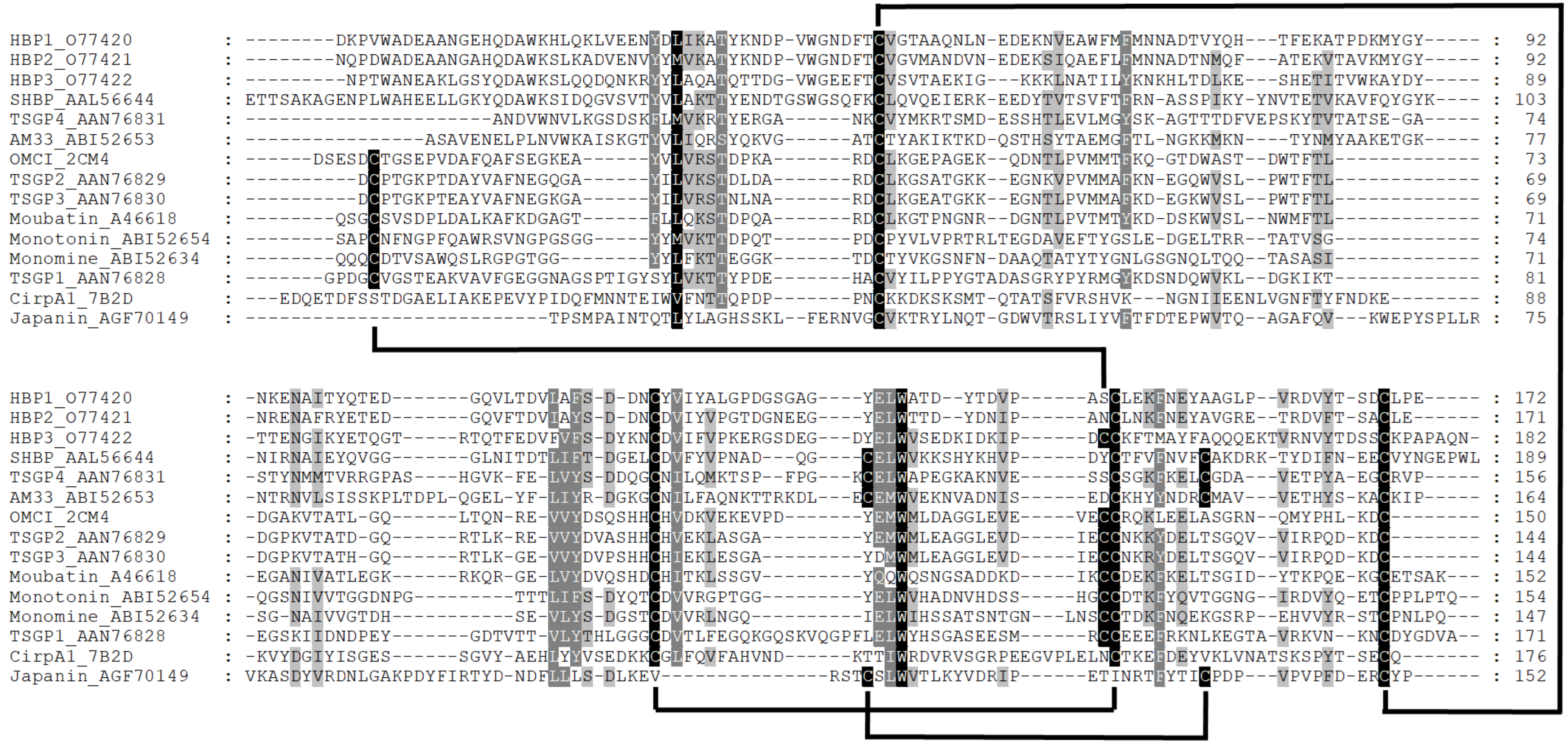

Figure S34. Alignment of lipocalins for which structures and/or functions has been determined. Indicated are conserved disulfide bonds. Also indicated are the names and accession numbers of the proteins. Functions include histamine and serotonin binding (HBP1, HBP2, HBP3, SHBP, Monomine, Monotonin, TSGP1), leukotriene B4 scavenging (OMCI, TSGP2, TSGP3, moubatin), complement inhibition (OMCI, CirpA1), leukotriene C4 scavenging (AM-33, TSGP4) and cholesterol binding and immune regulation (Japanin).

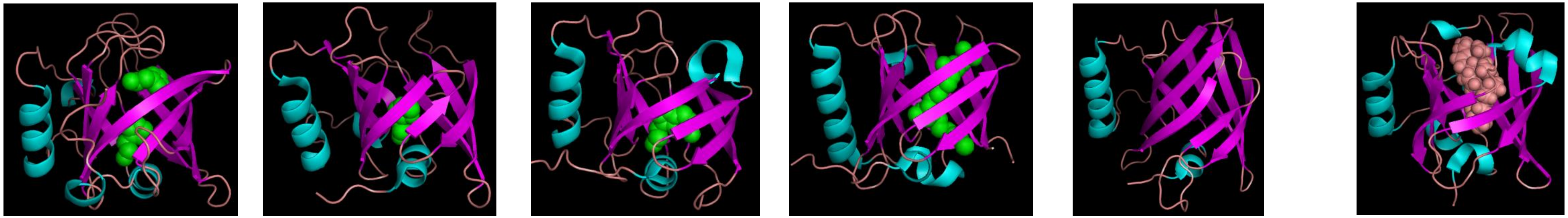

HBP1
Histamine

Monomine
Histamine

Monotonin
Serotonin

OMCI
Leukotriene B_4
Complement C5

Cirp1
Properdin

Japanin
Dendritic cells
Cholesterol

Figure S35: Structures for lipocalins whose functions has been determined. Indicated are the names of the proteins. Ligands scavenged or functions are indicated below names.

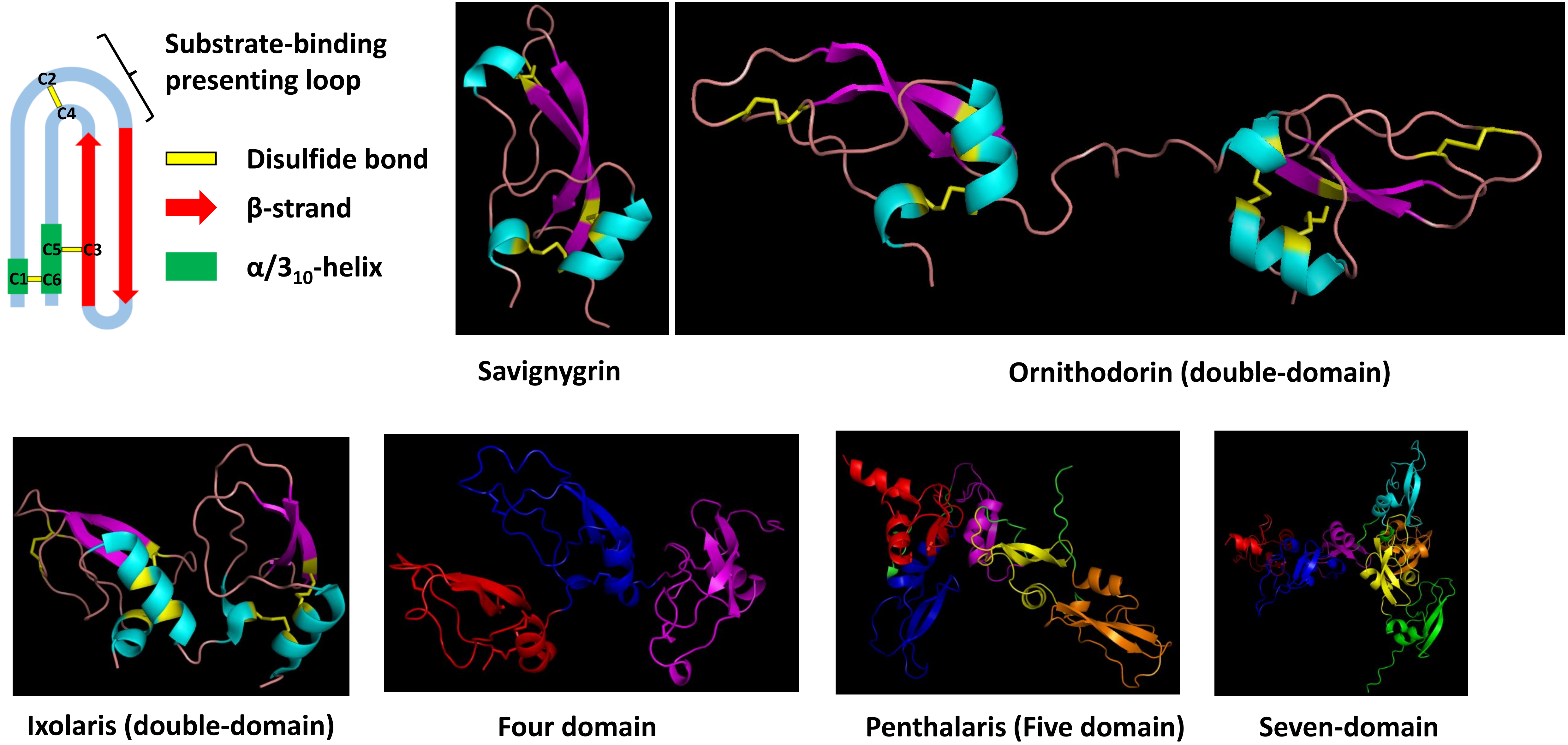

Figure S36: Summary of the Kunitz-BPTI fold. Top left indicate a schematic representation of the secondary structure, disulfide bonds and substrate-binding presenting loop. Also indicated are representatives for single, double-domain (ornithodorin-like), double-domain (bikunin-like), three-domain, five-domain and seven-domain models. For the single and double domain proteins, secondary structure is colored, while for the four, five and seven domain proteins, domains are colored individually.

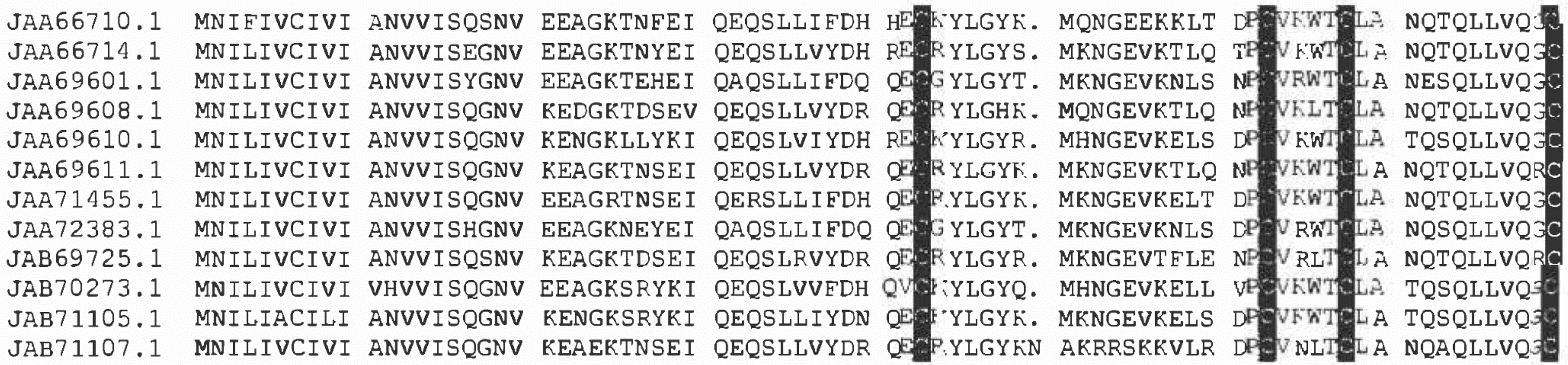

Figure S37A: Amino acid sequence alignment of *Ixodes ricinus* four-cysteine 8.9 kDa family members. Conserved cysteine residues are highlighted in black.

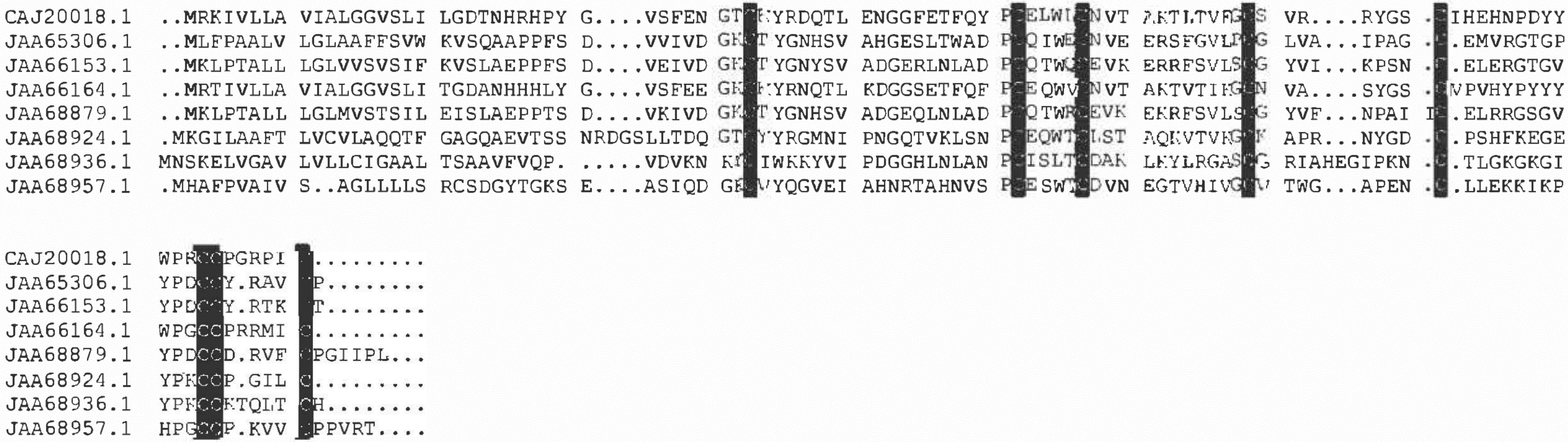

Figure S37B: Amino acid sequence alignment of/. *ricinus* eight-cysteine 8.9 kDa family members.

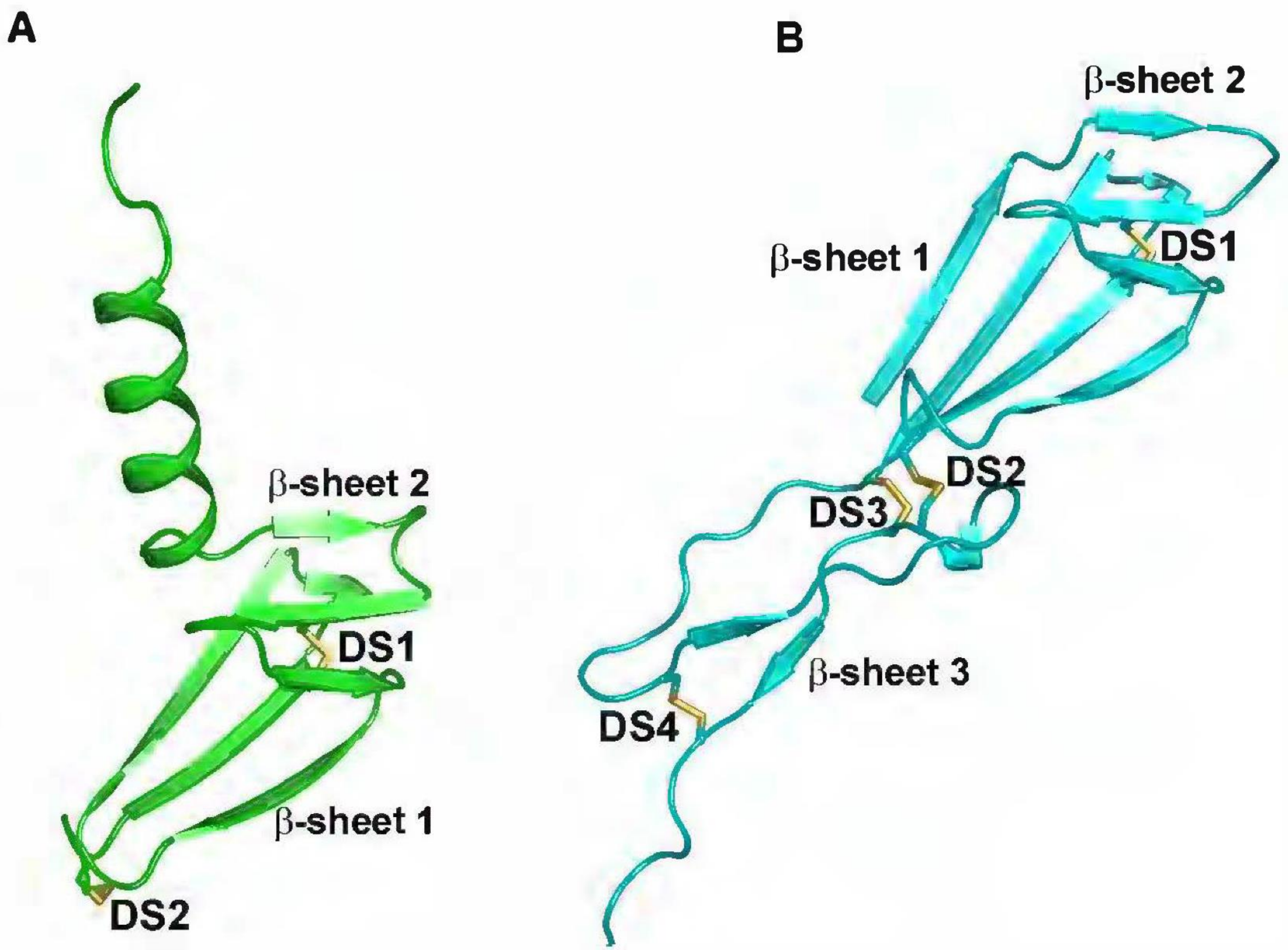

Figure S38: Structural models of four- (A) and eight- (B) cysteine 8.9 kDa family members from *Ixodes ricinus.* Cysteine side chains are shown as sticks with sulfur atoms colored yellow. Beta sheet structures described in the text are labeled.

```
AEO35217.1   ...MKKVPML LLIFGAEVAM ATRFSMMRDE SLHCQDGECF FRGYEVKVGV PVSMEKPCEQ WNRRESVEYP EVFVK.GCDM KIVSERTLPE .YIEQDESNL
AEO35215.1   ...MKKVSVL LFVFGVEVAI ATRFSMMRDE SLHCQDGECF FRGYEVKVGV PVDMENPCEE WNRQKSVQYP EVFVK.GCDV KIVSERTLPK .HIDQNESNL
AEO35216.1   ...MKKVPML LLIFGAEVAM ATRFSMMRDE SLHCQDGECF FRGYEVKVGV SVEMEKPCEE WDLQKSEQYP EVFVK.GCDG KVVSERTLPK .YIDQNETNV
AEO36432.1   MASVCKAIKL IVLFGPLCLT SGVKYVSKQY ALTFEDGQCK FEGLNMPYGG EGFLFG.CVF LKCDYEN..K TVTMY.GCPP ...PPYVLPL SDYGADSNDI
AEO36553.1   ......MNIL FFVICLLCPV YTTL.YNTDD DQRVDGKACV IGPLSLSAGD TRYSNRPCVK AQCIANP..P QLIIT.GCSA DGVNDYTLS. ...EGKNIVV
AEO36758.1   ......MHTL LLCLAIFLTL AVIYCDGKKN GGKSK.GNCE YGDRTIPNGQ SRNLQNPCVK VKCNDGT..P TVTECPGASV K.LEERRRGK .RSGKRKEGV

AEO35217.1   WPKCCKK... .
AEO35215.1   WPKCCQK... .
AEO35216.1   WPSCCQK... .
AEO36432.1   WPNCCPGYEV E
AEO36553.1   WPGCCDRDD. .
AEO36758.1   FPQCCEEDSS .
```

Figure S39A: Amino acid sequence alignment of *Amblyomma maculatum* six-cysteine forms having two alternative disulfide bonding patterns

```
AEO35167.1   ....MSMLVA LLCTLASFTL IICGTPDVVG RTYVVKNVKV IN.GSCIYVG NQIPDGEQKS LHYPCENVTC NAEEREVTAV MCKDFGVGDG CTLKLINEGV
AEO35493.1   ....MMQLMV LSFAVLLHSM GAT....HAE VFNSTGSLVF VN.GTCWYNG LKIDDDTYQS FKKPCSQLWC SASKGYLTFY GCMRPLEKPY CGVP..VDQV
AEO35708.1   ....MRIIVA SLCALVVFAT VICDIQEH.G QSYLTRNVTI VD.GSCIFER NTLPDGETKA LHEPCVIATC YAERREVNAT [illegible]CRNFGVDPG CRVHWTPDGV
AEO35865.1   MLEVKKLSTS KDMLTFLLIT ITLGAHIVDG EDVEVSSLLF VN.GTCLYRN VQLSDGESQA QNEPCENWTC DNKNKRLKVQ GCNLENKYGS CVYTNSGRRT
AEO35866.1   MLEVKKLSTS KDMLRFLLIA ITLGAHIVDG EDVEVSSLLF VN.DTCHYRD TVISDGESQA QYEPCENWTC DISKKHLKVQ GCNLEDKYGS CVYTNRGTWP
AEO35932.1   ....MCSKLS LAVYAIVAVT GTLWH..ISE AYSGQVHVEV LN.GTCIYAN RTLGHRRSIQ LKEPCEGWYC DANRRIIFVG WCSPVALLGH CRTVK.GNGT

AEO35167.1   FPACCP..RQ ECPDGKKTV
AEO35493.1   YPKCCS.YTR TC.......
AEO35708.1   YPECCP..RQ VCDRTD...
AEO35865.1   WPYCCRY.ER TC.......
AEO35866.1   WPYCCSY.QR TC.......
AEO35932.1   YPNCCP..RM VCN......
```

Figure S39B: Amino acid sequence alignment of *A. maculatum* eight cysteine forms

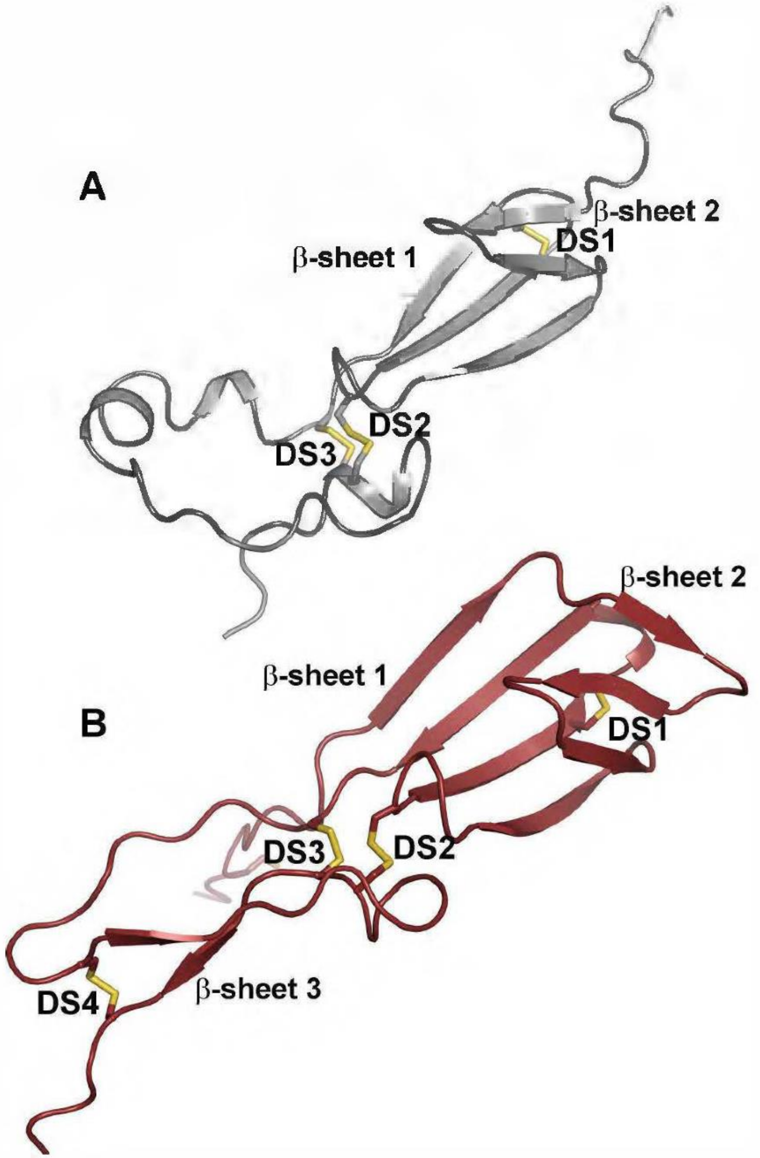

Figure S40: Six- (A) and eight- (B) cysteine 8.9 kDa family members from *Amblyomma macu/atum.* Cysteine side chains are shown as sticks with sulfur colored yellow. Disulfide bonds are labeled (DS). Beta sheet structures are labeled as in the text.

```
AEO35686.1  ...MLVRALL CGLILPMANS FDS..SDLNI LTVS.VTMEG GK..CVYKNH TFPSYYDPSG DNCELWTCNK PKHTVSVMGC SPLPPGCERK PKANSTFPKC
AEO36153.1  MCTMLHYLLF CGIVLLVLHN HGS..SGLKL VEVSSVKVEN GN..CVYGSV SFPTYFQTNG SLCEDWTCEP KKHTLTIVGC SPPRPGCRRN TSVDLRFPVC
AEO36311.1  ...MFASVLL FGCISHLVDC ASN..SDMKI VVAT.ATVVG DK..CVYKDK TFSSIFVPDA PVCEQWTCDK KRGTVNILGC SDPSPGCTRI ASGGAKFPDC   16 Cys
AEO36678.1  MRNRGSNILP CAMLSLLSLG RANILENVKY VYVQNVKVDE IKHTCEYRNY TFNSTLVLTD .PCMSLECYW RKQEVVISEC KQPKRNCVQR PSEGKVFPEC
AEO36727.1  ...MLQRILL FGLALELATS FDN..SDLKT WLAT.VQMDG DK..CIYRNY TFALYYNPKE .ACEDWTCNK REKTVSVVGC SDPPTGCIRN ASSDAAFPEC
AEO36739.1  ...MLLCGLL SALLLWVPRS RAEDVSNVKQ VEIKNPQLDK GY..CAYHTS AFNGAILPSG .LCERWTCKY NEGKILKEEC KALEHGCKR. SNPKARFPEC
AEO36783.1  ...MLAFPLL GALLAWLCPL SVEGIHSIRR IISTSPNIPK GI..CVHYPY FINGTLTVPG .ECRTLTCYY HQGQVLIEEC QPLEHDCQR. VNSSAPFPFC
AEO36784.1  ...MLAFPLL GALLAWLCLL SVEGIHSIRR IIATSPNIPK GI..CVHYPY FINGTLTVPG .ECRTLTCYY HQGQVLIEEC QPLEHDCQR. VNSSAPFPFC   12 Cys
AEO36785.1  ...MRAFTLL GALLAWLSHR PVEGIHNLRR ITVSNPNIPR GI..CAYYPY FINGTLDLPG .KCQKLTCYY RQGRIEIEEC QLLEHGCVR. VKPDAAFPHC
AEO36864.1  ...MLLCGLL ITLLLWVPLS RAEDVSNVKQ VEIQNPQLDK GY..CAYHTT AFNGAIIPPE .ACERWTCKS NEGKIIKEEC KELEHGCER. KNPKAKFPRC
AEO36918.1  ...MNRVMAI MSILLRLAHA QAP..EDLKV VEFTNVTLHE DEY.CEYKNS TFRNKVEVAA PVCKTITCLH SQGKVRISEC APLKPGCERD PEPMSFYPEC

AEO35686.1  CEKSCLNKTT PPCQTPDGTP LLEGGEYNST KP..CLRYEC LNGTLIMEE. CPHPEP.TDP HCSRSLAEEA .PYPACCGAG IVCQHKGKAK GKGKGRGKDG
AEO36153.1  CEEICLEATQ PYCVAPDNTP IMEGLEYNST DP..CGRYMC QNKTLIQVAG CPELEAKDDP SCQPSFAERA .PFPACCPAA VVCTSSG... ..........
AEO36311.1  CSTTCVSTSN PACEAEDGIL VYEGHTYNSS KP..CVQYAC NNGQITKTR. CPGADD...P LCQSSFADPE QPFPCCCGAA HVCTSNT... ..........   16 Cys
AEO36678.1  CQTTCFEETY R.CLTPDGVL LEDGQSFSCT SP..CVKYTC RKGTLETER. CRTSND...P FCAVSQADSS RPYPLCCGSI .VCSRRRR.. ..........
AEO36727.1  CEKSCVRLTD ATCTTPNNIL LADGQAYNST DP..CVRYEC NNKTITTVR. CS.EPP...P GCTRDSTRSS YGYPTCCEIR ..CVKTASP. ..........
AEO36739.1  CETQCLEKSS PFCTTPDNVL LLYGDSRQSH VSGKCVKYTC ENGNLVESK. CENQ...... .......... .......... .......... ..........
AEO36783.1  CEKKCLPRTN PYCTTPGGVL IPNGVSWTTQ SP..CMRHTC KDGKVETKR. CSRRKRSNER LFAVYPAFNL K......... .......... ..........
AEO36784.1  CEKKCLPRTN PYCTAPDGVL IPNGESWTTR NP..CMRYTC KDGKLETQR. CSRRRRSNKM FLP....... .......... .......... ..........   12 Cys
AEO36785.1  CETKCLPRTN PYCTAPGGVL IPNGGSWTSR NP..CMRYTC NDGRLETQK. CSRRRRSI.. .......... .......... .......... ..........
AEO36864.1  CETKCLEKSY PFCTTPDNVI LPYGGSRESK GSGNCVKYTC ENGKLKESK. CDNE...... .......... .......... .......... ..........
AEO36918.1  CRTKCFQPKH S.CVTPGGYY LLPHGFWFYI HRIPCFYVVC IDGIFAAGR. CGMPPQWRWP .......... .......... .......... ..........

AEO35686.1  KNKRKKRSSK KNL
AEO36153.1  ..KRRRR... ...
AEO36311.1  ...GRK.... ...
AEO36678.1  .......... ...
AEO36727.1  ...PEALANK PG.
AEO36739.1  .......... ...
AEO36783.1  .......... ...
AEO36784.1  .......... ...
AEO36785.1  .......... ...
AEO36864.1  .......... ...
AEO36918.1  .......... ...
```

Figure S41: Amino acid sequence alignments of *A. maculatum* twelve- and sixteen-cysteine 8.9 kDa family members.

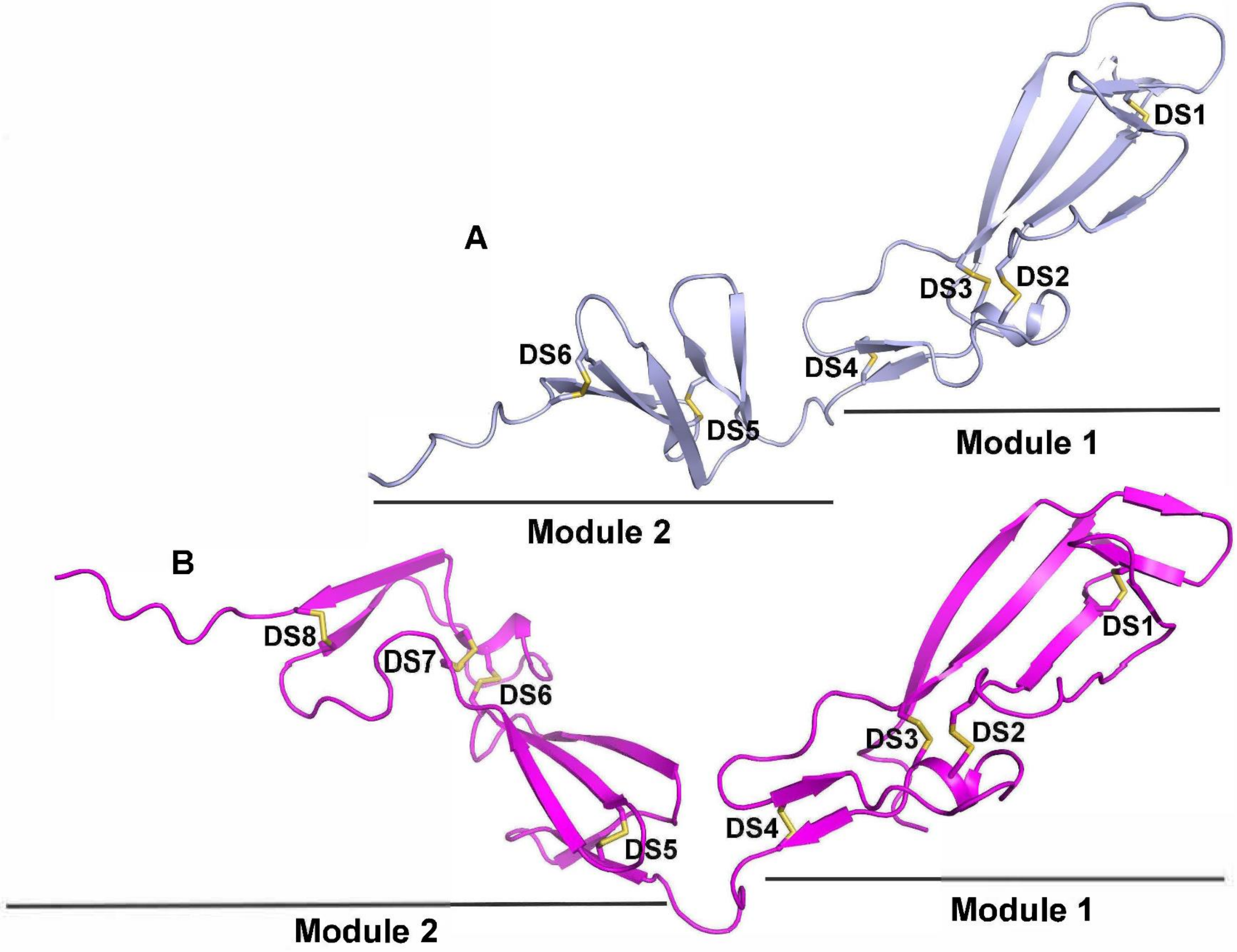

Figure S42: Structural models of *A. maculatum* twelve- (A) and sixteen- (B) cysteine forms. Cysteine side chains are shown as sticks with sulfur atoms colored in yellow. Disulfide bonds are labeled DS. Repeated modules are indicated by bars under the models.

```
JAP77261.1  .MSTALVILF AACAASVVGQ FYDRCPQRE HRHGC DFLG RIIGRGLTVS LRIC LRVTC HPDGS.VTSI GCPREAG.SN PHPGAPPTR. .GVWQCC DM
JAP77703.1  MKCLFAAIVV LLIVSLLRRS FA.IKCPARF DRQGCH VFG LTVPRGHTVA VPNPCV SVTC NHNG.HMLVK GCINFAGNME VHPGSPDEAR VLPYRCC PR
JAP80461.1  MKYSLLVISL VAYVAVVKC. VF...CPARF ANGRC FIFG RVILDGLAWT FNNPCV QVSC NYHGRTITIK GCPGNE.... .AP.GAP.QP MNRYRCC HN
JAP84679.1  MKLSAALLIL VASFSFAKRR PG.ITCPARH TPVGC FFLG HFIPAGESSA SWNPCV FVSC SRNGQTVSIT GCPSVAE.PR QHAASAPTLR PQGYRCC ER
JAP84931.1  MNIPAVGILL AVQVALVMTQ V...QCPVRY GQRGC TAFG QTIPAAGSAN LRNPCV HVKC SANRHRVTVT GCQDVAG.LN QHPPGAPGGP WGDWPKCC SR
JAP86317.1  MMISALAILF SAWAALVLCQ YYDRCPVRN HPIGC QFMG RLLFPDTSQL WRLPCLIVSC AVDGS.VTTQ GCPRTTG.RN PHPGAAPGP. .GVWPKCC DM
JAP85507.1  .MTTALVILF AACAASVVGQ FYDRCPQRE HRHGC DFLG RIIGRGLTVS LRIPCLRVTC HPDGS.VTSI GCPREAG.SN PHPGAPPTR. .GVWQCC DM
JAP85780.1  MKMLTVALAL ISLSVLVACH IR..C PVRS VRGACW YDG RLYPAGSHVS SINPCA FITC SHTGQTLTIT GCPGTAG.GD EHPPGAAGVP LLFYPKCC SR

JAP77261.1  C G......
JAP77703.1  C GPAPRFR
JAP80461.1  CD IL....
JAP84679.1  C RPV....
JAP84931.1  C PALPRG.
JAP86317.1  C S......
JAP85507.1  C G......
JAP85780.1  C PRIR...
```

Figure S43A: Amino acid sequence alignment of *R. appendiculatus* eight-cysteine 8.9 kDa family members with alternative disulfide bonding pattern. Conserved cysteine residues are highlighted in black.

```
JAP86702.1  ..MMIPVILI CSVLHLFLVF EIVP..GQSV NTYK.VNTTD EGQCH FNGTY YPNGNFSTWE PRCYMALCN T ...TARELTL WCN ARRIPG.C LMPKAPKD
JAP77355.1  ....MHTFGA SLFVLVVISV VYADVSERGH TYVTKNVTVE DGACV YLRNV IPNGETKALN NPCV LSTC YA ...ADRKVNS TLCPNIGVDE GCHVEWTPDG
JAP81018.1  ....MTKLLT CFVIFITGTL FQVDI.SWQA LYPD.PSAYR NGKCYFRGGV YKPGENMYDQ PRCMKWGC SK INSTSGSMVG VSCGVVVASP PCKVTPLTTG
JAP81333.1  ....MTKLRT CLVLFITSVL CLVEV.SWQA ......TPYK EGKCYFEGKF YEPGENIYTK .RCSMWSC IK TNSTHTYMHE KCV RPLVQM GCRRLPGKEG
JAP81374.1  ....MSLVHK CLMVTMSVLC AGCGI.CNGY MGRT.PVEIK NGHCTLDGRT IRVNQSVSLQ DIC EEWTC DY ...VSKSVVI ACSPTEAGP DC KMVDGK.G
JAP85717.1  .....MWFRS FLLFTATVVL AEWHA.ANGY VSIA.EVPVE NGECDFNGTK VAPGHPLNLE EIC EEWTC ST REGQTGHLSI ACGAVGAGA GC RKVKGT.G
JAP86658.1  MSGAVSVLAL CVVATLFIFD PWMAC.GQLV TVIKEVNVTD QGQCH YNGTY YPKGNFSTWI PKC YMAIC KS ...ARKVLKI IC DAKPVHG.C PLPPVPQD

JAP86702.1  GYACCV EPIC.......
JAP77355.1  VYPNCCPKHV C PSASATS
JAP81018.1  IYPKCCPRRV C P......
JAP81333.1  IYPKCCPRFL C P......
JAP81374.1  DYPDCCPQML C Y......
JAP85717.1  VYPDCCEKVI C D......
JAP86658.1  GYPACCPEPV C.......
```

Figure S43B: Amino acid sequence alignment of *R. appendiculatus* eight-cysteine 8.9 kDa family members with conventional disulfide bonding pattern. Conserved cysteine residues are highlighted in black.

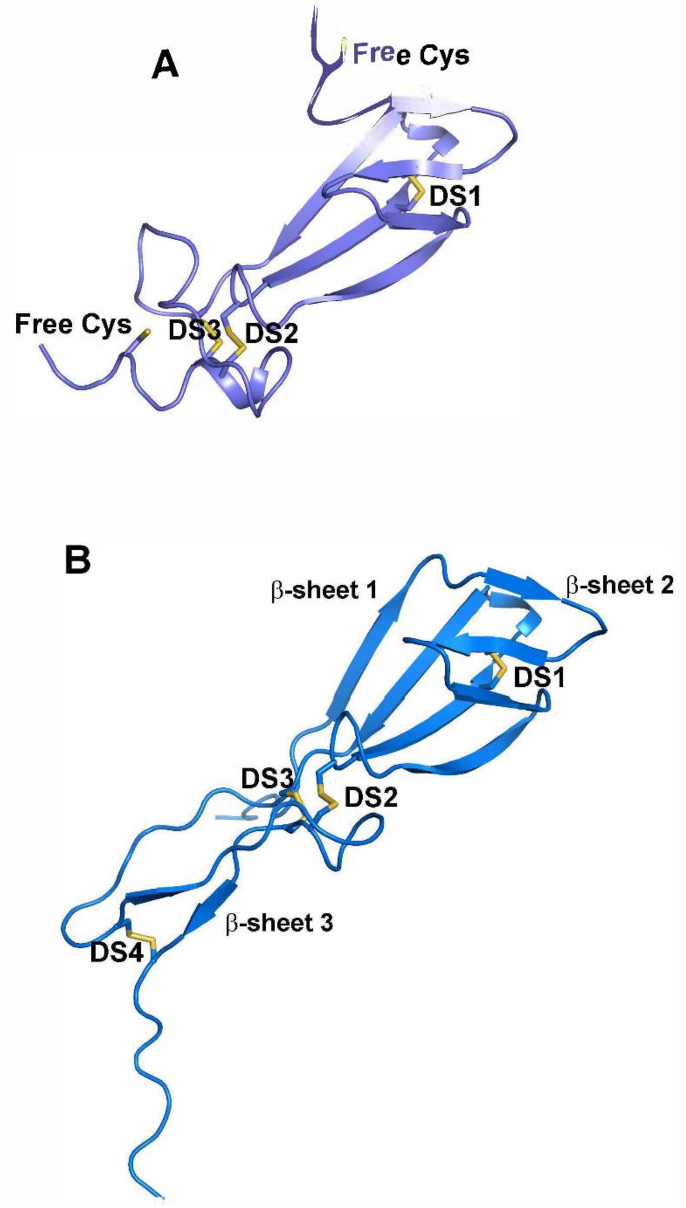

Figure S44: Eight-cysteine cysteine 8.9 kDa family members from *R. appendiculatus* with alternative (A) and standard (B) disulfide bonding pattern. Cysteine side chains are shown as sticks with sulfur colored yellow. Disulfide bonds are labeled (DS). Beta sheet structures are labeled as in the text.

```
JAP77355.1   MHTFGASLFV LVVISVVYAD VSERGHTYVT KNVTVEDGAC VYLRNVIPNG ETKALNNPCV LSTCYAADRK VNSTLCPNIG VDEGCHVEWT PDGVYPNCCP
AEO35708.1   MRIIVASLCA LVVFATVICD IQEHGQSYLT RNVTIVDGSC IFERNTLPDG ETKALHEPCV IATCYAERRE VNATLCRNFG VDPGCRVHWT PDGVYPECCP
AEO35710.1   MRILVASLCA LVVLATVICD IEEHGHSYLT RNVTVVDGSC VFERNTLPDG ETKALHEPCV IATCYAERRE VNATLCRNFG VDPGCRVHWT PDGVYPECCP
JAG91758.1   MRALVS.FCA LSAFATVICD IQEHGHSYLT RNVTVENGAC IFEHNTLPDG ETKALHDPCV IATCNAARRE VNATLCRNFG VDPGCRFHWR FVGVYPECCP
JAG91783.1   MRAFVASLCA LVAFATVICD IQEHGHSYLT RNVTVENGAC IFERNALPDG ETKALHDPCV IATCYAARRE VDATLCRNFG VDPGCRFHWR NDGVYPQCCP
JAG91784.1   MRVLVASLCA IVAFSIVTCD IQEHGHSYLT RNVTVENGAC IFERNTLPDG ETKSLHDPCV IATCYAARRE VNATLCRNFG VDPSCRFHWR NDGVYPQCCP
JAC23742.1   MRAFTASFCA LVAFATVICD VQEHGHSYLT RNVTVENGAC IFERNTLPDG ETKALHDPCV IATCYAARRE VNATLCRNFG VDPGCRVQWT PDGVYPQCCP
cirpt1       .......... .........D VQERGHTYVT KNVTVEDGAC VYLRNVIPNG ETKALNNPCV LSTCYAADRK VNSTLCPNIG VDEGCHVEWT PDGVYPNCCP
cirpt2       MRTLVASLCV FAVFSAVCCD VQERGHTYRT RNVTVEDGAC VFERNVIPDG ETKALNSPCV LSTCYAAARE VNSTLCPNIG VEQGCRVEWT PVGEYPNCCP
cirpt3       MRTLGVSLFV LVGISAVYCD VQERGHTYVT KNVTVENGAC VFERNVIPDG ETKALNSPCV LSTCYAADRK VNSTLCPNFG VAEGCHVEWT PDGEYPNCCP
cirpt4       MRAFVALFCT LVAFATVICD IQEHGHSYLT RNVTVENGAC IFERNTLPDG ETKALHDPCV IATCYAARRE VNATLCRNFG VDPGCRFHWR NDGVYPQCCP
```

```
JAP77355.1   KHVCPSASAT S.
AEO35708.1   RQVCDRTD.. ..
AEO35710.1   RQVCDRTD.. ..
JAG91758.1   RQVCDGTD.. ..
JAG91783.1   TQVCDGTD.. ..
JAG91784.1   RQVCDGTD.. ..
JAC23742.1   RQVCDGTN.. ..
cirpt1       KHVCPSATAS S.
cirpt2       KHVCPTTS.. ..
cirpt3       KHVCPAAPAT S.
cirpt4       TQVCDGTD.. ..
```

Figure S45: Amino acid sequence alignment of sequences from *Amblyomma* and *Rhipicephalus* species grouping with C5-binding complement inhibitors CirpT1-4. The sequence block containing selected residues contained in the interaction interface with complement factor C5 are highlighted in green. CirpT1 is from *Rhipicephalus pulchellus*, CirpT2 is from *Dermacentor andersonii*, CirpT3 is from *Rhipicephalus sanguineus* and CirpT4 is from *Amblyomma americanum*.

```
JAG91563.1  ..MMASAKLL SFALLVLTTV AMIGVPSADT SSVPYGPDVI LPC.RGNYIC FIHTD..G.. RQYGCPQ... GGKCIPNGNY DQHGYAQGKC FCAHG.....
JAC19583.1  .......... .......... .......... YSGHFFDEAI YQC.YPRVDC FRHKD..G.. WQS.CPAHLP GAQCIPNRRF NERGFASGVC FCRGR.....
JAA60894.1  .......... .......... .RPGPPG... .....SAGVM LVC...GDPC YIRND..G.. VAKGCPE... ECRCVSNKFA RGIYTGEGIC WIERWRNSTP
JAG91534.1  ...MTLNKLL SFALLAVTIV MMMGRCS... ...AYHDNAF WQC.YNAGPC YRYPN..G.. TQQGCPP... YCGCVSRRYY EGIYYGLGNC VALPQYG...
JAU03243.1  ...MTLTKLL YFALFAVTIM AMMGRCS... ...AYGNDVI RQCPITPGPC YIHHN..G.. MQEGCPW... TCRCISNKYN QGIYNGWGRC FTNRRTG...
JAG91532.1  ...MTSAKRL SYPVYVVALL AMIGICSQ.. VAQGYGNDAI GLC.PESGVC FIRSD..G.. RQAGCSG... DCRCISDRYN EGIYDGWGHC YG........
     Raci1  ...MNAMLVL .FIASALFIS EHNTEEV... ..KTTPIP.N HQC..VNATC ERKLDALGNA VITKCPQ... GCLCVVRG.A SNIVPANGTC FQLATTKPPM
     Raci2  ...MNAVTVL AFTAFALIVH DCYSEEA... ..NTTPISVK DQC..ANVTC RRTVDNRGKR HIDGCPP... GCLCVLKG.P DSKDNLDGTC YLLATTPKST
     Raci3  MAALNGLVLL LLTISAMFIS ECYSSGE... ..SQS.IQRK GQC..EEVIC HRKLNHLGER VTSGCPT... GCLCVIRE.P DNVDNANGTC YALMSSTTTT
```

```
JAG91563.1  .......... ....
JAC19583.1  .......... ....
JAA60894.1  .......... ....
JAG91534.1  .......... ....
JAU03243.1  .......... ....
JAG91532.1  .......... ....
     Raci1  APGDNKDNKE EESN
     Raci2  TTSTEQSFNM EE..
     Raci3  TTTPDGTTTS EEEE
```

Figure S46: Alignment of 8kDa family members. Raci1-3 are known inhibitors of the complement pathway from *Rhipicephalus appendiculatus.* Cysteine residues are highlighted in black.

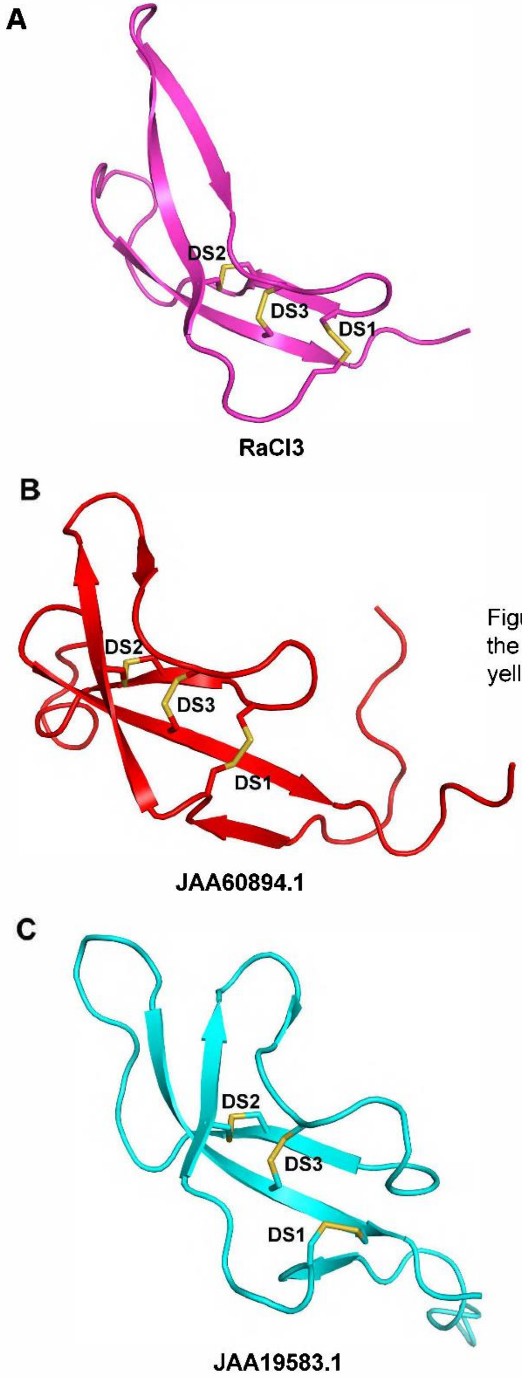

Figure S47: Alphafold2 models of two uncharacterized 8 kDa family members compared to the crystal structure of RaCI3. Cysteines are shown in stick format with sulfur colored yellow. Disulfide bonds are labeled DS1-3

```
JAG92232.1  .MNV....AL FFLLFSLGIL NLAE...... ASSSQSGTTT TKVAECHGTE CISS.ADGQT RCPPGCECYY YEEYG....G NYCV...QAG AGSGDYVYG.
JAC19098.1  .MKG....AL VCLLSVLSLI TTAE...... SSGSSP.... P.AKTVTCD. .AQC..DEQY RCQAGCECAA YDYEG..TEV YYCSLPDGTA DYANTYNGG.
JAC27161.1  .MKS....VL VCLLS.LQFL TITE...... ASGSSS.... S.AKAVTCP. .EEC..DGNY GCGDDCECVT DEDE....IE YFCTQAGGGS ..TADYNGG.
JAC27594.1  .MKS....SW ILLPFALGIL DVA....... GRIILQ.... ..SSSCGGT. ..CNPYENDG WCSEDCYCEL G.......FP ...LPMQGYC TPAEYASASV
JAC27201.1  .MKY....AL LFILSVVGSL H.KT...... ASGSHS.... ..YDACHQA. ...CHLEKGV SCPTGCSCAE YGTG.....A YRGR...GVC QLNRGWTSEQ
JAC27947.1  .MKT....AL GIVLFAVGFI LTVR...... ASGSSR.... .SPAACVGQ. .VCDAYDSQA GCPPPCFCQP YDAG...DGT VVSYCITGNG DYGDSGSGGG
JAG92094.1  .MKS....AL LLLLSVLGSL R.NE...... ASGSQS.... ..YDVCHQT. ...CNTDRGL RCEVGCSCGE Y......... YHGW...GVC QLKPGWTAER
JAC27126.1  .MKY....PL LFILSVVGSL H.KI...... ASGSHS.... ..YDACHQS. ...CHTDRKV YCPTGCSCAE YGTG.....H YQGR...GIC VLKPGWTSER
JAG92061.1  .MKA....AL LLLLSIIGSL R.KE...... ATGSQS.... ..VDVCAQR. ...CVCRLGL YCDYGCSCGE Y......... YYGE...GVC HIKPGFTQEQ
JAC27906.1  MMKI....AY YIMMSVLGFI QIAE...... GSGSQK.SAP TPKTVFTCQE SACNPYNEGP QCGPHCECAY YEEDG....Q YLGVCTTADG VDGGDYQP..
JAC27858.1  .MKA....SL ILFLFTIGFL QHAL...... GSPREQ.... ..KNTCGGT. ..CNPYEGDG WCSEECYCEM D.......FP ...LPLWGYC TRAEYASIGT
JAC19077.1  .MRS....VL LILLLLVDVL HLSKACNGCE RNAKDA.... ..YQGCLGT. ..CDPDEPFK GCSKGCLCYD GGT.....YP .DGTPWGGIC YAAPEGGVED
JAC27917.1  .MPGKWGYLY FFLLVMQHLL RVSQSCDWCD RSAKDP.... ..YQGCLGT. ..CDPYKPYE GCGSGCWCYH GGT.....HP VEGYDMGGFC YEADE.DNEP
JAG92276.1  .......... .......... .......... .SSSSS..ST TKQPTCAGQW CSRDQSTEEY SCGPGCACYY YESDGDESDG GYVCSAYAAG AANTDYSPG.
JAG92164.1  .MLR....FE YFLLHMLSLA HASH...... RNAKEP.... ..CQGCLGT. ..CNPDKPFE GCGSNCLCYD GGI.....HP VEKYEIGGIC YDAPEFGDDD

JAG92232.1  SS........ .QRVDPNVAV QASTQLISDA LQVAAYSRGS ...LPSIKKP SLKLPKMKLP KFKMPNVK.. ...LPRLRMP RIRFRGRR.. .....
JAC19098.1  PG........ .GATDPAYVA QTVSSMLTDT VQMASSLRRP ...SGLAKKP S..FLSKMKS RISLRKVR.. ...LPKVKSS DSDFQDAAED LPLTF
JAC27161.1  GG........ .GGVDPTAIV QSATSLAGEA INMASLMRRP ...KEVSR.. ........DH HVSRR..... .......... .......... .....
JAC27594.1  D......... ...VEF.... .VAKSSEHDA KQS....... ..QNYIENKN .......GTD DIA....... .......... .......... .....
JAC27201.1  VQ........ .ALLDAQDVM ETVTEVGGEL VKKVYKKLRN L....KITKK .....MSKAI KAGMKIIKK. ...LVKALKG LVRFRG.... .....
JAC27947.1  GG........ .TGIDPNTAL MSSTGLAGNL LNTVPYWKLP KIKKPSLPSL P....KLNLP KVGLSKVSFG KNLLSKIKRP KFKMPRIG.. .....
JAG92094.1  VT........ .AVIEAQAVM EAVLEASGDA VKHGMSKVSK IK.MPKIKLP .....KMKGL RFGIGRLMK. ...LLKTLSR ILRFRG.... .....
JAC27126.1  VQ........ .AEIDAQEVV ETVVEVGGQL VKKVLKN... .......... .......... .......... .......... .......... .....
JAG92061.1  VA........ .AAIDANAVI EAVTQISGDL LKKGINKMSK V....KIKLP .....KIKMT RFGVSKIMK. ...LIKIIKK LLRFRG.... .....
JAC27906.1  YS........ .SGVDPNVAA QSGTELAGSV VQALAIARPK ...SSPLKK. ......LKIP LQKMKSS... ....IKKIRP KLSIRKQK.. .....
JAC27858.1  DTMDALMDLG ELKAEMPKTP KLAGASSNTA KSPKISAPTV RFPKFTAPKM P....RLSLP KITLP..... ...K...VFT RG........ .....
JAC19077.1  IDY.....TT AHTAVAATGL ALQSAVAVSS AVRRVSFGSS AARKAVSSAL SK..VKVRMP RIKI...R.. ...IPRFKLS RG........ .....
JAC27917.1  KE......DN SQTAATAIQL TATTLQAGAA VASRIPISKV KIGRAAAKFA A....KIRMP KIKL...R.. ...IPRFKLS RG........ .....
JAG92276.1  YQ........ .SSIDPNVAL TSATSIFGDV MNAAALSGKP SFKTPSFIKN IRRVSLPKMP SIKMPKLK.. ...IPKFTRP KLRLRIRG.. .....
JAG92164.1  KR......QG GVNKEGLQKF TEEITKSGGE ITRRIPWSKT ALARATSKWA T....AVRVP RIKLPKFR.. ...IPRIRTS RG........ .....
```

Figure S48:Amino acid sequence alignment of 15 kDa basic proteins. Cysteine residues are highlighted in black.

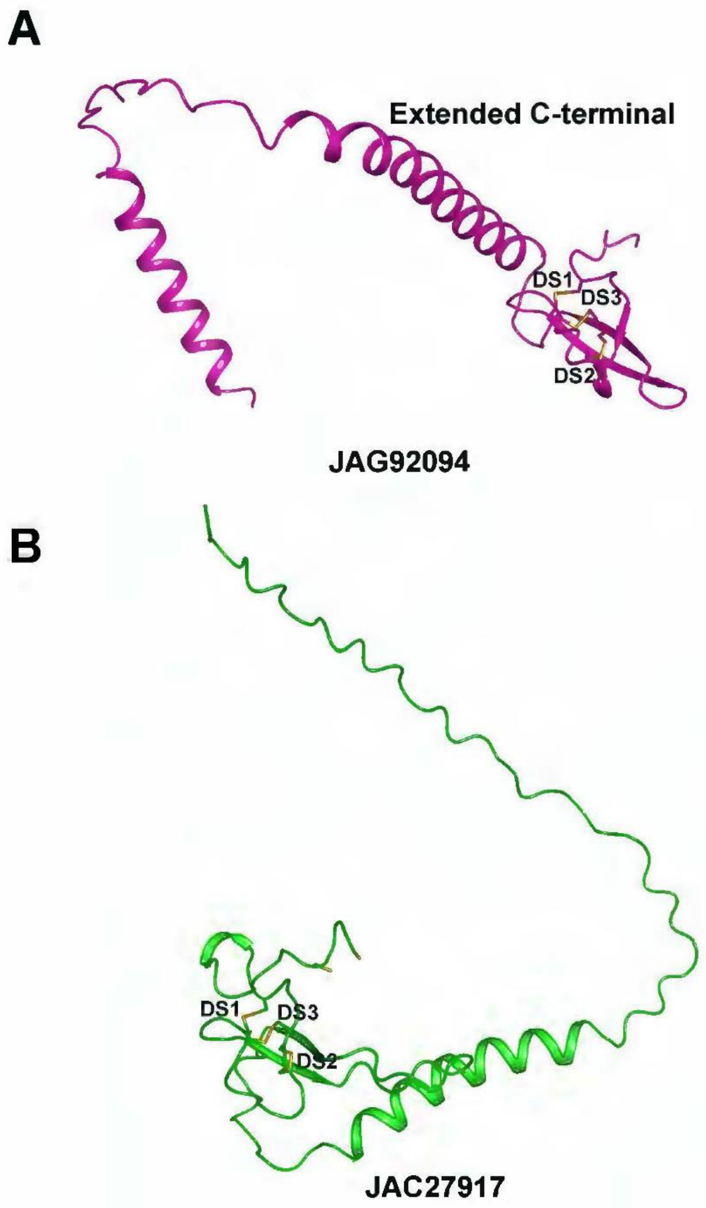

Figure S49: Alphafold2 models of 15 kDa basic proteins. The proteins contain a small domain similar to the 8 kDa proteins with a long C-terminal portion that does not fold into a distinguishable domain.

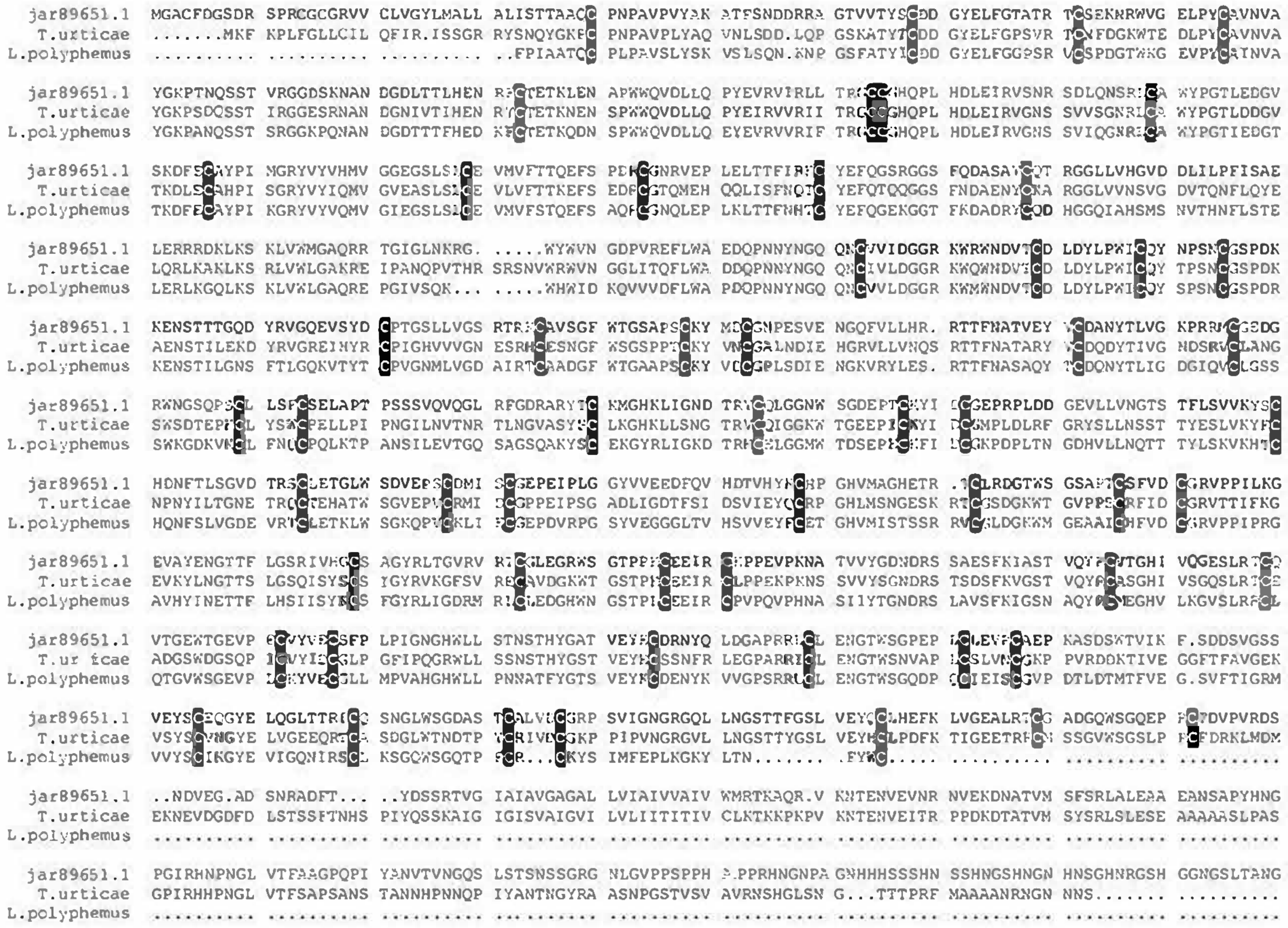

Figure S50, Amino acid sequence alignment of sushi domain-containing proteins having lectin domains at the N-terminal end from a variety of arthropod species. Numerous cysteine residues are all predicted to participate in disulfide bonds. These are highlighted in black, T.urticae: *Tetranychus urticae,* L.polyphemus: *Limulus polyphemus.*

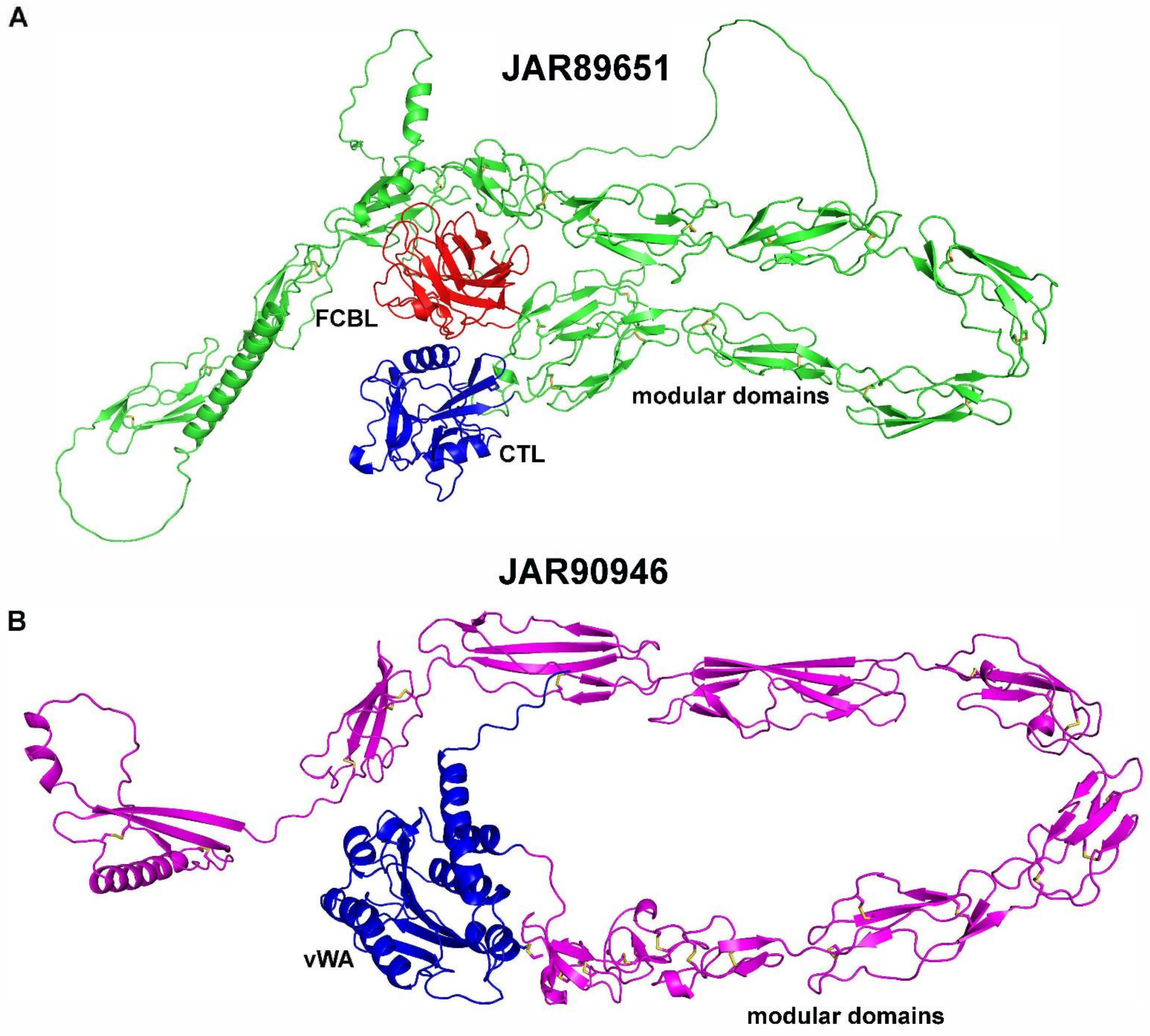

Figure S51. Structures predicted by Alphafold2 of JAR90946 containing lectin domains and JAR89651 containing a von Willebrand A domain at the N-terminal end.

```
  JAR90946.1   ........ME VLKLLCSFLS LLLVPVDVAD .......... .........A QPVKTFPEAT E...DSERMD QLGAVLKRHV AKLRSTAR.. AELVFLVDSS
L.polyphemus   ........MN IFWYLVGLLF VAAGLSQAAS SGGYRHTRNK EYEQFFNSGA KPSFTSNERL PNQLKKNKVE ELGNILKQHV AALRRTPNKQ VELVFLVDSS
V.destructor   MYFSSESATN RWVFFLAIFY IARVSSTLN. .......... .......... EIVEYPLSTI DATIDIKKLE QQSERLKSSL GALHNNYEHG IDIAFLVDSS
P.vannamei     MRAKASYPEG RRRLLMAVLS FLSLMVLAPV AVAQYKTERP PYPDFLNYGK KKFTKPGIEL ENEIIKSKVE VLGEILKSHV HQLRQAPGQR VELVFLVDAS

  JAR90946.1   ASVGAGNFLN ELRFVRKLLA DFTVS...LD ATRVALVTFS SKKRVLREVD HITRPTD... .TNHKCLLLN TQLAHVKYAG GGTFTLGAVL EAQDILKSAR
L.polyphemus   ASVGSENFFN ELKFVKKLLA DFTVS...YE ATRVAVITFS SRERVVKDVD HLSNPSE... .NNHKCALLR DQLPSINYTG GGTYTLGAVL KAQEVLKYAR
V.destructor   LSVGLANFKL ELRFVKKILA DFSISHLRKN TTRVTLVTFS SPDKVVTHVD FISPNGSSQI LNRHKCSLMA .ELAQIQYVG GLTYTLGAMK IADRVLGASN
  P.vannamei   ASVGAENFFN EIKFVKKLLA DFAVS...YN QTRVAVITFS SKSKVIRHID HVTQASP... .DNHKCTLLE TELPRIKYSG GGTYTLGAML EAQSVLALAR

  JAR90946.1   EDAVKVVFLV TDGYSNGGDP RPAAQELRDA GTLVYTFGVR NGNVRELRDM ASLPADEHCY ILNSFEEFEA LARRALHEDM HVGPHQNLNS SDCDGLCK.E
L.polyphemus   PESTKAIFLV TDGYSNGGDP RPAAKDLRDQ GVQIFTFGIK NGNVRELHDM ASEPSKEHCY ILDSFEEFEA LARRALHEDL QIGSYLPQKS EACLRLCQGE
V.destructor   RSVPQVVFLV TDGFSNGGDP KPIARKLKSR GVTIFTFGIK NGNVKELQDM AT.TRKEHCF IVNSFEEFEA LARRALHQDL SVGDFEPLDL SQCNGLCP.A
  P.vannamei   NDARKAVFLV TDGYSNGGDP RPAAAFLQAR GVTIFTFGIR NGNVKELYDM ASVPKEEHSY ILDTFEEFEA LARRALHEDL HSGFLIHQPP RTCGKLCE.E

  JAR90946.1   GSECCHSGAQ CTCGIYSGQY SCLCPKGHYG SGMRGECHPC PQGTYQPSVS SGDKGICLPC PNAHQNSAPG STSVMQCHCR RGYSAEGHS. ...CRVVHCQ
L.polyphemus   SKSCCDARAQ CTCGTHTGHY ACLCQPGYYG TGLQGNCEPC PEGTYHVGSG PGDISSCQSC PDAQHTSPLA STSPNQCTCK PGYVTNGKK. ...CEVIECP
V.destructor   GQLCCQSGAR CACGTTSGYF SCLCPRGQYG TGLKGECKPC PKGTYQPRFV HGGIEECLPC PGSHQTSPSG SHSKQQCSCK SGFTTLITGN SSVCMPVRCP
  P.vannamei   GTYCCDADAA CTCDTHTGHY ACTCRKGYYG TGILGDCRPC PPGTYRTHQG PGDVSSCSPC PDPHMVSDAA STSAQQCYCK RGFKQVGQE. ...CLMLQCQ

  JAR90946.1   KLRPPANGYL VNSDCGVVFN AACGFRCNPG YRLIGNSIRV CQQSGTWSGS DPVCEKKKCQ TLGAPFHGSI KCSTEAFDFE TVCEFECQRG YVLIGSRKRS
L.polyphemus   PLSPPEHGYF VNGICSSVIH AACGIRCDSG YKLVGSSVRL CTVDGTWSGT NVRCEKKTCS SLSAPHHGSV LCTTDSNVFE TKCHFSCDPG YKLVGSKIRT
V.destructor   ALVAPLNGFL VNEKCESVFN AACGVACETG YKLSGPSILV CNQDGRWSGE MPKCHKRKCQ ELTAPNSGWM KCTTRSYQFE TECEFGCSEG FVLVGSKRRN
  P.vannamei   VLAPPENGYF VRNTCNNVVN AACGVRCNPG YTLKGSSIRM CGENGEWTGW NARCVMKSCQ KLKPMAHGTM VCTQPTPIMD TECHFTCDPG YQLVGSKMRT

  JAR90946.1   CLSIALWDGL PTLCRPVSCP PLLAPANGIF SPAHCSETKS AFGETCYLRC NDGFASPHQA SRECVHPGLW SGNDTAIMCM DLEPPTIRNC PEDMEVDSEP
L.polyphemus   CLAIALWDGL PALCRPVDCP PLSPIFNGHV SPAKCTESKM RYGDVCIYTC KPGFVVRGPA TRRCEDQGVW GNEEKKTVCI DVEPPVLR.C PEDIVVDAEE
V.destructor   CLAVAHWDGL PATCRQIYCP ALDIPANGRI FPLSCNASRT TFGDQCRYRC QEGYRLTGPS SRECIYPGVW TDHQLVTRCV DTKPPSIE.C PDNILVPADP
  P.vannamei   CLPVAMWDGI PAYCKPIFCP RLPPLLNGRI RPGSCSSSKS KYGSRCEFAC DPGYQISGPI KTTCIDPGVW SEGHKSPRCV DITPPVIQ.C PENITTETEP

  JAR90946.1   GYAEALVDWE LLEASDNSG. ESLMLSVVPA VMPPQLFPIG T.SDITYWAE DAAGNKAFCN FSVVVRDREP PMDDSCTSPS DAFSNGSPSA VVTWDEPVFS
L.polyphemus   NERDYMVTWN VPQGIDNSG. EIPSVSVLPA VIPPRRFAIG T.ATITYTAE DRSHNTASCS FMVTVRDVQP PTIDKCVSPP KFLSREAP.V TVFWEEPIFS
V.destructor   GEPYATVDFS LPSIQDNSGF QEITLTVSPA VEPPLPFFIG DPMNITYTAT DSQENKNSCT FSVTVIDEEP PTVDRCESPA VTLSSDGQGA KVVWEEPLFS
  P.vannamei   NEVYANVTWN PPHVKDNSK. GKIKLHTVPA TSQPMKMKIG N.HTITYVAM DKLGNKASCE FSVTVVDKEP PRIDECLSPP VFLSYEEV.V DVYWEEPVFS

  JAR90946.1   DNSGIDPVVW QSHEQGTTFP VGETLVTYVA SDASGNNASC ILRVVVRDHR CIMPVAPANG DVDCRKTSSG IACRITCLQG YGLQPRVPSE YTCAFDGSWS
L.polyphemus   DNSGSPVNIR RSHSPG.LFP LGETVVTYEA VDEAGNNNTC NLTITVQEHA CSIPSDPVNG KANCTESDSG VNCSLSCEEG YAFVTSPPYN YFCAYDGVWL
V.destructor   DNSNEDVFIW SSHKPGQYFP INDTVVTYNA ADNSGNNASC TFNITVKATK CEPSFSVLNG KLNCTVDALQ TLCHIQCDRG FTLLPNLP.N YLRCIDGVWN
  P.vannamei   DNSGVDVKIT RSKEPG.QFP QGDTMVEYWA EDAAGNVATC NITITVQKHA CQMPVDPING AANCTENPDA VFCTLTCDDS YAFAMRPQQD YFCAYDGMWL

  JAR90946.1   P.HSIHSFPD CSESSPP... ...SSATVKA RLFYSA.FGI SCGDRLTQKE LREHLLRRLS MQAAR.CPDG VDCSVGDLHI ECPTVQAEPN DAESSE.LHR
L.polyphemus   P.RDRFPFPD CAVTQLS... ...NAISQTG IITISS.DGG DCDDQFLLNQ VKSHLHRKIE KKVASLCKEN MLCNVNNFAT MCDNTFEETE EETNAI.ISR
V.destructor   ISDIDLPSCT TVVTSSSGSS SKLTSSSAST PVVLPSGTMF ECTNEFLSQQ LSVVLKKRIS MKNVIKCRAK PKCYKEKLKP ICEVTQRNML PISSHA..AR
  P.vannamei   PDENPMPFPD CSVTSVS... ...NSISQYG EMAMGEEDDS ICDDIFFMGQ VENQLEKKLE DALTAMCSDD VVCEVAAVEA VCESILADAE EEFNSIGFFR

  JAR90946.1   ERRSKRAEDI EVSFDITG
L.polyphemus   KKRNAVFLTK .......T
V.destructor   KRRS.VS... ........
  P.vannamei   RRRSAEEDAL NRYWPQRH
```

Figure S52. Comparison of vWA domain containing protein JAR89651 with representatives from non-blood feeding arthropod species. Cysteine residues are highlighted in black and all are involved in disulfide bonds in modeled proteins. *L.polyphemus: Limulus polyphemus*, V.destructor: *Varroa destructor*, P.vannamei: *Penaeus vannamei.*

```
JAC30591.1  MKTVAVPSVL AFLLLLEIVS GGYICQDPRP YLSTYRVRSD GHECTSLVKN MSTVLGDYTT NSWVAGVRVR DNC.DIPRWT AIATFLGPGG KYNTGAVNDM
AEO34830.1  .......... .......... ..ISCSNPES LKGNWVIGVD GKECVALVKE KCSGMRQYST HSWRRGKHVR SNCGSIPRWT AIATFLDG.S KY........
ACX53960.1  MKGFVI.SVG LLLLGMTVVT QAIRCADPTG FKGGWVIGVD RKECVALVKE KCKGLRQYTT HRWRRGLQVR SNCRKVPRWS AIATFLDG.K SY........
JAP80515.1  MKGFVV.SLG LLLLGMAVVI QAIRCANPTS FKGRWVIGVD RKECVALVKE KCKGLRRYTT HRWRRGIRVR GNCRKVPRWS AIATFLDG.R SY........
JAC30544.1  MNGFLL.CAA LLVLGMAVGS QAISCSNPES LKGNWVIGVD GKECVALVKE KCSGMRQYST HSWRRGKHVR SNCGSIPRWT AIATFLDG.T KY........
JAA60761.1  MKGFVV.SVG LLLLGMAIVI QAIRCVNPTR FKGRWVIGVD RRECVALVKE KCNGLRRYTT HRWRRGIRVR GNCRKVPRWS AIATFLDG.R SY........
JAP66903.1  MNTSVV.FAA LLVLSMDIAI QAIRCADPSR YKGRWVIGVD GRECVALVKE KCKGLRRYTT HRWRRGLHVR KNCAKVPRLS AIATFLDGGK RY........

JAC30591.1  AKQHAAIFDT CESNGLWVYD QNRNRPVNRT FFKDLGGDDT TSNARNYRVI ELR
AEO34830.1  .RGHAAIFES CAPDGIWVYD QWNTAKVDRR KIR.YGNSKP NYNGDNFYVI EL.
ACX53960.1  .RGHAAIFES CAKDGIWVYD QWNTAPIKRR KIY.YGYRKL NYNGNNFYMI KL.
JAP80515.1  .RGHAAIFES CAKDGIWVYD QWNTAPVKRR KIH.FGYRMP NYNGNNFYMI KL.
JAC30544.1  .RGHAAIFES CAPDGIWVYD QWNTAKVDRR KIR.YGNSKP NYNGDNFYVI EL.
JAA60761.1  .RGHAAIFES CAKDGIWVYD QWNTAPVQRR KIR.FGYRMP NYNGNNFYMI KL.
JAP66903.1  .RGHAAIFLS CASDGIWVYD QWNTAPLKRR KIR.YGYKAP NYNGNNFYMI KL.
```

Figure S53. Amino acid sequence alignment of Dae-2 protein family members. The catalytic cysteine and histidine residues are highlighted in green. Disulfide bonding cysteines are highlighted in black.

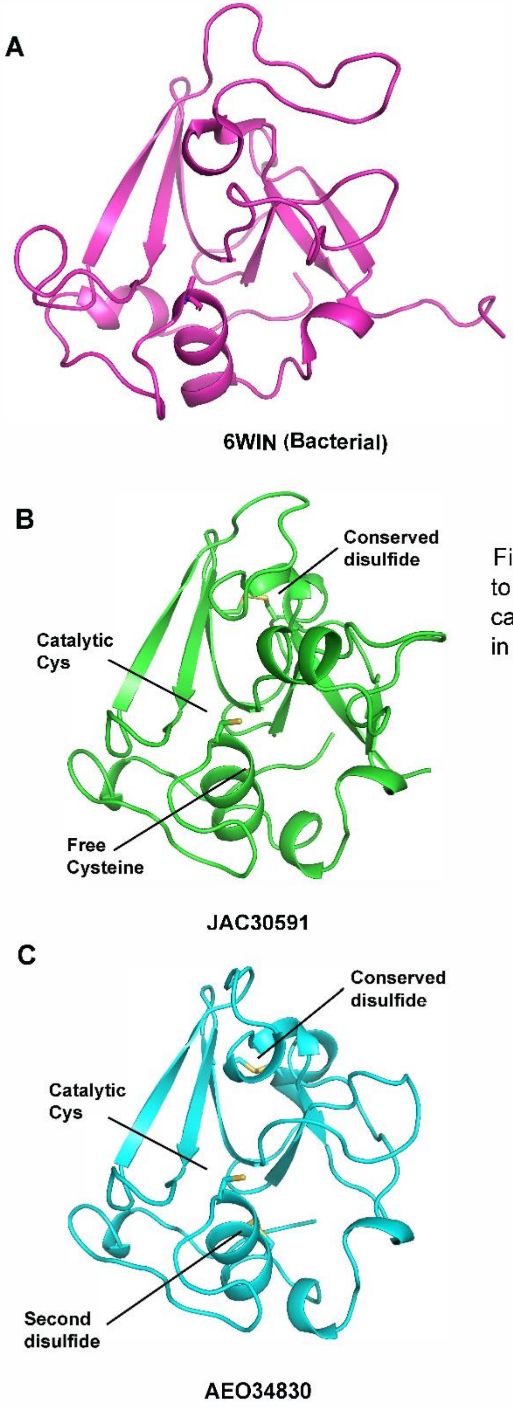

Figure S54. Structure of bacterial Tae-2 (PDB 6WIN) compared to Alphafold2 models of tick salivary proteins (Dae-2). The catalytic cysteine and additional conserved cysteines are shown in stick representation and sulfur is colored in yellow
