# Supplementary material for: A Deeper Insight into the Tick Salivary Protein Families under the Light of Alphafold2 and Dali: Introducing the TickSialoFam 2.0 Database
Source: Int J Mol Sci. 2022 Dec 9;23(24):15613. doi: 10.3390/ijms232415613 (PMC9779611; doi:10.3390/ijms232415613)
Supplement: Supplementary file 1 [file ijms-23-15613-s001.zip › Supplemental file S1.pdf]

Supplemental file S1: Disintegrin motifs in prosite format used to scan tick salivary proteins using the program ps_scan.pl (https://github.com/ebi-pf-team/interproscan/blob/master/core/jms-implementation/support-mini-x86-32/bin/prosite/ps_scan.pl)

ID   RGD_cys disintegrin; PATTERN.

PA   C-x(0,16)-R-G-D-x(0,16)-C.

/

ID   MLD_cys disintegrin; PATTERN.

PA   C-x(0,16)-M-L-D-x(0,16)-C.

/

ID   KGD_cys disintegrin; PATTERN.

PA   C-x(0,16)-K-G-D-x(0,16)-C.

/

ID   VGD_cys disintegrin; PATTERN.

PA   C-x(0,16)-V-G-D-x(0,16)-C.

/

ID   KTS_cys disintegrin; PATTERN.

PA   C-x(0,16)-K-T-S-x(0,16)-C.

/

ID   RTS_cys disintegrin; PATTERN.

PA   C-x(0,16)-R-T-S-x(0,16)-C.

/

ID   ECD_cys disintegrin; PATTERN.

PA   C-x(0,16)-E-C-D-x(0,16)-C.

/

ID   WGD_cys disintegrin; PATTERN.

PA   C-x(0,16)-W-G-D-x(0,16)-C.

/

ID   RED_cys disintegrin; PATTERN.

PA   C-x(0,16)-R-E-D-x(0,16)-C.

/
