# Supplementary material for: A Deeper Insight into the Tick Salivary Protein Families under the Light of Alphafold2 and Dali: Introducing the TickSialoFam 2.0 Database
Source: Int J Mol Sci. 2022 Dec 9;23(24):15613. doi: 10.3390/ijms232415613 (PMC9779611; doi:10.3390/ijms232415613)
Supplement: Supplementary file 1 [file ijms-23-15613-s001.zip › Supplemental materials header.pdf]

Supplemental Material:

1) Supplemental Figures: PowerPoint file with manuscript figures and movies.

2) Supplemental File S1 - Disintegrin motifs in prosite format used to scan tick salivary proteins using the program ps_scan.pl (https://github.com/ebi-pf-team/interproscan/blob/master/core/jms-implementation/support-mini-x86-32/bin/prosite/ps_scan.pl).

3) Supplemental spreadsheet S1 – Hyperlinked spreadsheet containing putative tick salivary proteins linked to comparisons to several databases and AlphaFold predicted structures. Clusterization of the proteins allowed for extraction of reversed-position specific motifs collected into the TSFam 2.0 database. The spreadsheet has links to pdb files, which need programs that are able to open them. We suggest the use of ChimeraX (https://www.cgl.ucsf.edu/chimerax/download.html) or Swiss-PDBViewer (https://spdbv.unil.ch/).

4) Supplementary TickSialoFam 2.0 database – Include rps models and a formatted database which should be used to query protein sequences by means of the rpsblast program from the NCBI Blast suite of programs (https://ftp.ncbi.nlm.nih.gov/blast/executables/blast+/LATEST/).
